# Extended analysis of benchmark datasets for Agilent two-color microarrays

**DOI:** 10.1186/1471-2105-8-371

**Published:** 2007-10-03

**Authors:** Kathleen F Kerr

**Affiliations:** 1 Department of Biostatistics, University of Washington, Seattle, Washington, USA

## Abstract

**Background:**

As part of its broad and ambitious mission, the MicroArray Quality Control (MAQC) project reported the results of experiments using External RNA Controls (ERCs) on five microarray platforms. For most platforms, several different methods of data processing were considered. However, there was no similar consideration of different methods for processing the data from the Agilent two-color platform. While this omission is understandable given the scale of the project, it can create the false impression that there is consensus about the best way to process Agilent two-color data. It is also important to consider whether ERCs are representative of all the probes on a microarray.

**Results:**

A comparison of different methods of processing Agilent two-color data shows substantial differences among methods for low-intensity genes. The sensitivity and specificity for detecting differentially expressed genes varies substantially for different methods. Analysis also reveals that the ERCs in the MAQC data only span the upper half of the intensity range, and therefore cannot be representative of all genes on the microarray.

**Conclusion:**

Although ERCs demonstrate good agreement between observed and expected log-ratios on the Agilent two-color platform, such an analysis is incomplete. Simple loess normalization outperformed data processing with Agilent's Feature Extraction software for accurate identification of differentially expressed genes. Results from studies using ERCs should not be over-generalized when ERCs are not representative of all probes on a microarray.

## Background

Recently, the MicroArray Quality Control (MAQC) Consortium published a series of papers on an important effort to address ongoing issues concerning the reliability of microarray data [1-6]. Some specific goals of the MAQC project include generating reference datasets using multiple microarray platforms produced across multiple laboratories; establishing reference RNA samples for the scientific community; measuring the reproducibility of microarray data; and evaluating the advantages and disadvantages of various data analysis methods. For the complete list of MAQC project goals see [4]. The article by Tong et al [6] addressed the goal of evaluating data analysis methods for microarrays. This particular study examined datasets from hybridizations that contained External RNA Controls (ERCs), elsewhere referred to as "spikes" or "spike-ins." Tong et al [6] reported results for five different microarray platforms.

ERCs are extremely valuable for quality control because their true concentrations are known by design. Since one knows what the microarray measurement should be, one can examine how well the microarray gives the right answer. One aspect of the study reported by Tong et al [6] was to leverage ERCs to compare the performance of different methods of processing array data. For example, for the Affymetrix platform, Tong et al [6] process the data with five different methodologies for Affymetrix data: PLIER [7], MAS5 [8], dChip [9], gcRMA [10], and RMA [11]. Tong et al evaluated characteristics of the concentration-response curves corresponding to each of these methods.

Unfortunately, no similar evaluation of data processing methods was presented for the Agilent two-color data in [6]. While this is understandable given the broad and ambitious scope of the project, it can create the false impression that the community of researchers using this platform has reached consensus about the best way to process Agilent two-color data. Experimentalists using this platform need to be aware of the various data processing choices available. Indeed, further analysis of the MAQC Agilent two-color data reveals important differences among common choices for data processing. Additional analysis also reveals some important caveats to the interpretation of the results for these ERC datasets. These additional analyses of the MAQC Agilent data extend the good work in the previous report [6].

This paper examines six Agilent two-color MACQ datasets. Datasets were produced by three sites (1, 2, and 3) with two different RNAs (A and B).

## Results

### Comments on concentration-response curves

ERCs in the MAQC datasets have true log-ratio equal to ± log_2_(10) ≈ ± 3.32; ± log_2_(3) ≈ ± 1.59; or log_2_(1) = 0. Tong et al present a figure (Figure 4 of reference [6]) that shows the relationship between the observed log-ratios of the ERCs compared to the expected (true) log-ratios for the Agilent two-color arrays. Other than four arrays that clearly failed, the relationship is near identity. This tempts one to conclude that the data processing was completely successful. However, further analysis of the data reveals that the behavior of ERCs may not be representative of other spots on the array because the ERCs do not span the range of intensities

**Figure 1:**
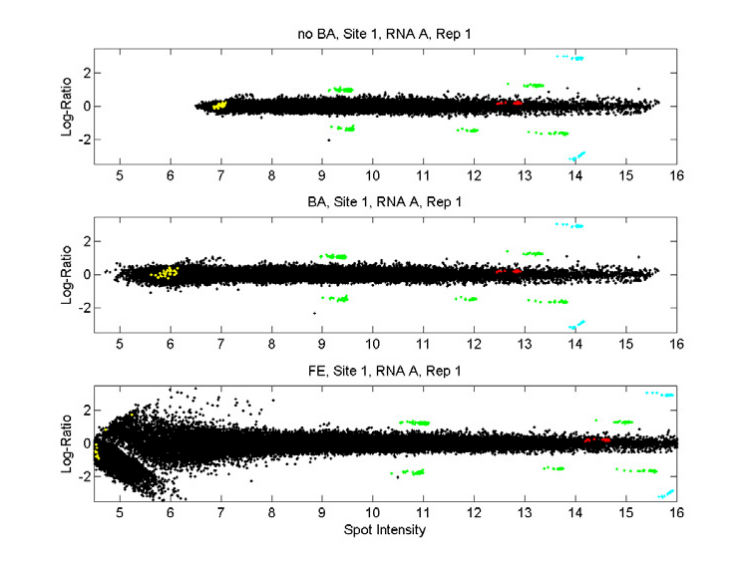
**Ratio-intensity plots for three methods of data processing**. Horizontal axes represent the average log_2_(red) and log_2_(green) signal as a measure of spot intensity. The vertical axes represent the log-ratio of red and green signal. These ratio-intensity plots are for replicate 1 from Site 1, RNA A (AGL_1_A1 in the nomenclature of [6]). Blue points are ERCs with true log-ratio = ± log_2_(10) ≈ ± 3.32; green points are ERCs with true log-ratio = ± log_2_(3) ≈ ± 1.59; red and yellow points are ERCs with true log-ratio = log_2_(1) = 0; black points are non-ERCs and have true log-ratio = 0. Top panel: noBA data (loess normalization, no background adjustment). Middle panel: BA data (loess normalization, with background adjustment). Bottom panel: FE data (data processing by Feature Extraction).

Figure 1 shows ratio-intensity plots (RI plots; also known as MA plots) of the data from one array in the MAQC study. The colored points represent the ERCs and the black points represent other genes on the arrays. The horizontal axes represent spot intensity. Note that the ERCs span only the middle to high end of the intensity range on the log scale. (The ERC represented by the yellow points in Figure 1 was apparently not used in Figure 4 of [6].) The nice behavior of the ERCs at medium and high intensities should not be expected to represent the behavior of genes in the lower half of the intensity range. See Additional file 1 for ratio-intensity plots of all arrays.

### Variability of non-ERC probes varies substantially with data processing method

The datasets considered here have the same RNA in the red and green channels. That is, other than ERCs, all spots have true log-ratio = 0. The true log-ratio is therefore known for every probe on the array, so these arrays are informative about the effectiveness of data processing methods. The bottom panel of Figure 1 represents the data as produced by the built-in normalization from the Feature Extraction software. This report will refer to this version of the data as the "FE-data." The top two panels of Figure 1 are two alternative versions of the data. In both cases, intensity-dependent normalization of log-ratios was carried out with a loess smooth [12] on the ratio-intensity plot. The top panel shows the data without any background adjustment ("noBA data") and the middle panel shows the data with local background subtraction ("BA data"). The variability of observed log-ratios is clearly larger for the FE version of the data than the BA or noBA versions, especially at lower intensities.

### Data processing and detection of differentially expressed genes

One of the most common uses for microarray data is to detect differentially expressed genes. In the MAQC datasets, one hopes that the ERCs with true log-ratio 10, 3, 1/3, or 1/10 can be detected among the remaining genes with true-log-ratio 0. When detection is the scientific goal of a study, the most appropriate way to judge accuracy is with the sensitivity and specificity of detection. Similar to [13] and [14], three different metrics, or "ranking statistics," for gauging the evidence for differential expression were applied: the mean, the t-statistic, and the modified t-statistic used in the popular SAM software [15]. For the noBA, BA, and FE versions of the data and for each ranking statistic, ROC curves describe the sensitivity and specificity of detection [see Additional file 2]. Table 1 summarizes the ROC curves with the AUC measure (a perfect AUC is 1.0). Recall that there are six different datasets because 3 sites produced data using two different RNAs. Each dataset has 4 or 5 replicate arrays (the failed assays identified by Tong et al [6] were removed).

**Table 1:** AUC values for ROC curves

			No BA			BA			FE		
	Site	RNA	mean	t-statistic	SAM-statistic	mean	t-statistic	SAM-statistic	mean	t-statistic	SAM-statistic

Agilent Dataset	1	A	**0.998**	**0.995**	**0.998**	**0.998**	**0.995**	**0.998**	0.971	**0.992**	**0.996**
	1	B	**0.998**	**0.994**	**0.998**	**0.998**	**0.994**	**0.998**	0.901	**0.995**	**0.998**
	2	A	**0.993**	0.974	**0.992**	0.990	0.975	**0.990**	0.907	0.984	0.970
	2	B	**0.995**	**0.993**	**0.996**	**0.992**	**0.994**	**0.996**	0.810	**0.996**	**0.995**
	3	A	**0.999**	0.976	**0.995**	**0.997**	0.976	**0.995**	0.816	0.976	**0.991**
	3	B	**0.997**	**0.991**	**0.997**	**0.997**	**0.992**	**0.997**	0.760	0.983	0.840

AUC values for ROC curves summarizing the sensitivity and specificity of detection for the six datasets from sites 1,2,3 and RNAs A and B. A perfect AUC is 1.0. AUC over 0.99 bold; AUC under 0.95 underlined.

Detection was superior using the mean or the SAM-statistic compared to the t-statistic, corroborating the finding of [13] for another two-color platform. For the SAM-statistic and especially for the mean, detection was superior for the noBA and BA versions of the data compared to the FE data.

**Figure 2:**
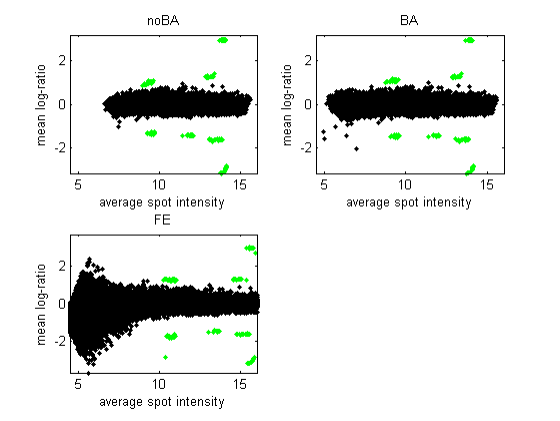
**Average log-ratios calculated from five replicate arrays**. The average log-ratio is plotted against the average spot intensity for the three versions of the data from Site 1 and RNA A (five arrays). Green points are the ERCs with non-zero true log-ratio.

**Figure 3:**
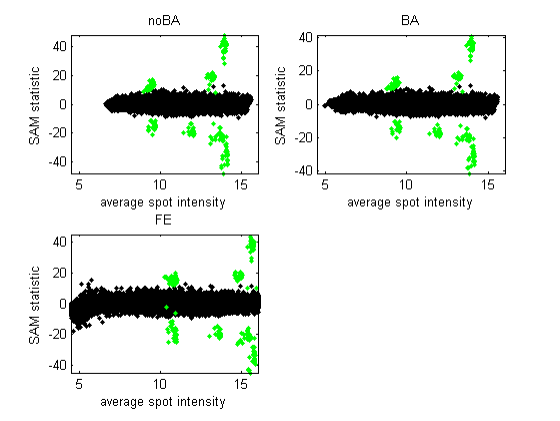
**SAM statistics calculated from five replicate arrays**. The SAM statistic is plotted against the average spot intensity for the three versions of the data from Site 1 and RNA A (five arrays). Green points are the ERCs with non-zero true log-ratio.

Figure 2 is similar to a ratio-intensity plot but summarizes the data from all five arrays in one dataset (Site 1, RNA A). Figure 3 is similar to Figure 2 but the vertical axis represents the SAM-statistic instead of the mean log-ratio. An effective ranking statistic will separate, vertically, the green points, representing the ERCs with non-zero log-ratio, from the black points, representing other genes or ERCs with 0 log-ratio. When the mean is used as the ranking statistic (Figure 2), many low-intensity genes exhibit a large average log-ratio in the FE version of the data. This is the case even though the average is over five replicates. The issue with the FE data is similar with the SAM statistic, although less pronounced in Figure 3 than with the other five datasets. [See Additional file 3 for the corresponding figures for all datasets.]

## Discussion

The analysis methods and findings here are very similar to the study by Zahurak et al [14]. The contribution of this article is to point out the omission in [6] with respect to the analysis of Agilent data, provide a more comprehensive analysis of those data, and to confirm that the findings on the MAQC data largely corroborate the findings in [14].

The three different ways of processing two-color data that were considered here (noBA, BA, and FE) produce nearly identical curves for the observed log ratios plotted against expected log ratios [see Figure 4 from [6] and Additional file 4]. That is, the behavior of these high intensity probes is nearly the same for the noBA, BA, or FE versions of the data. On the other hand, ratio-intensity plots and the ROC curves demonstrate that these data processing methods produce markedly different results for low-intensity genes. This is not news to those familiar with microarray data. However, it is not apparent in [6] that the ERCs only represent higher-intensity genes.

Tong et al [6] are careful to point out that the design of their ERC experiments was not ideal and make some recommendations for the use of ERCs in future studies. There is a current effort by the External RNA Control Consortium to develop a set of ERCs for the scientific community [16,17]. Given the importance of signal intensity for the behaviour of measurement, it seems crucial that an effective set of ERCs span the entire intensity range.

Microarray data with ERCs are extremely valuable for understanding the behaviour of the microarray signal and the operating characteristics of data processing methodologies. However, ERC probes may not be representative of all probes on a microarray, as seen here. Moreover, a single ERC experiment cannot be representative of all real microarray experiments, since different experiments will exhibit different patterns of differential expression. As a specific but important example, datasets in which only a handful of genes, the ERCs, are differentially expressed are extremely well-suited to the assumptions of loess normalization. Therefore, such datasets cannot be used to evaluate the effectiveness of loess normalization for data with lots of differential expression.

Clearly, the major difference among processing methods is the behavior of low intensity genes. One method for handling highly-variable low intensity genes is to simply discard them. However, Kerr et al [18] showed that microarray measurements on low-intensity genes are less reliable, but they are not unreliable. In [18], some measurements on low-intensity genes suggested genes that were differentially expressed between two RNAs, and these measurement were reproduced on "indirect" comparisons of the RNAs via reference RNAs. Therefore, the expedient option of simply discarding data on low-intensity genes can discard potentially valuable information on differentially expressed genes. It is desirable to identify methods of data analysis that are effective for low intensity genes rather than simply discarding these data. At a minimum, it should be acknowledged clearly when methods have been validated only for high intensity genes.

The results here show an advantage for alternative processing of the data over processing by the Feature Extraction software. Clearly, the FE data have greater variability at low intensities. This leads to worsened specificity of detection because some low-intensity genes with true log-ratio equal to zero exhibit large log-ratios. Zahurak et al [14] offer some ideas about the aspects of Feature Extraction that might cause exaggerated low-intensity variability.

In the alternative methods of data processing, which out-performed FE, there was no compelling evidence to favor or disfavor background adjustment (BA). However, Zahurak et al [14] identified a modest detrimental effect of background adjustment in processing Agilent data. Qin et al [13] found a dramatic detrimental effect of background adjustment on another two-color platform. For studies to identify differentially expressed genes, foregoing background subtraction seems the best course of action based on the limited current evidence.

## Conclusion

Choosing a data processing method is an important step in the analysis of microarray data. The MAQC datasets considered together with previous spike-in datasets [14] disfavour the Feature Extraction method for processing Agilent two-color array data. Ideally, future studies will use positive controls that span the intensity range of the data.

## Methods

There were six datasets from Sites 1, 2, 3 and RNAs A and B. All datasets had 5 replicates except (Site 1, RNA B) and (Site 2, RNA A) had 4 replicates due to failed assays. For each dataset, spots with any measurement in any replicate that were flagged as saturated were removed from further analysis. The median pixel intensity was used as the spot signal. For the BA data, the median background intensity was used as the local measurement of background and subtracted from spot signal. For loess normalization, the span was 4000 datapoints, or about 10% of the data. Note that each ERC was represented by 30 spots on the arrays and these were treated as separate "genes" in ROC analysis. The SAM-statistic is the classical t-statistic with a constant δ added to the denominator. In this analysis δ was set equal to the 90^th ^percentile of t-statistic denominators. Scripts for ROC curves and AUC calculation were downloaded from [19].

## Competing interests

The author(s) declares that there are no competing interests. 

## Authors' contributions

KFK analyzed the data and wrote this report. The author read and approved the final manuscript.

## Supplementary Material

Additional file 1 Ratio-intensity plots for all arrays. The plots show log-ratios compared to signal intensity for three versions of the data. Click here for file

Additional file 2 ROC curves for the mean, t-statistic, and SAM statistic. The plots summarize the sensitivity and specificity for detecting differentially expressed genes using three different test statistics applied to three versions of the data. Click here for file

Additional file 3 Test statistic values plotted against average spot intensity. The plots show the behavior of test statistics as a function of signal intensity for three versions of the data. Click here for file

Additional file 4 Observed log-ratios compared to expected log-ratios. The plots show observed log-ratios compared to expected log-ratios for three versions of every array. Click here for file
