# Supplementary material for: Extended analysis of benchmark datasets for Agilent two-color microarrays
Source: BMC Bioinformatics. 2007 Oct 3;8:371. doi: 10.1186/1471-2105-8-371 (PMC2174956; doi:10.1186/1471-2105-8-371)

Supplement 1: Ratio-Intensity plots for all arrays.

On each plot the horizontal axes represents the average log_2_(red) and log_2_(green) signal as a measure of spot intensity. The vertical axis represents the log-ratio of red and green signal. Blue points are ERCs with true log-ratio = ±log_2_(10) ≈ ±3.32; green points are ERCs with true log-ratio = ±log_2_(3) ≈ ±1.59; red and yellow points are ERCs with true log-ratio=log_2_(1)=0; black points are non-ERCs and have true log-ratio=0. In each trio of plots, the top panel (noBA data) is the data with loess normalization and no background adjustment, the middle panel (BA data) is the data with loess normalization and location background subtraction; the bottom panel (FE data) is the data as processed by the Feature Extraction software.


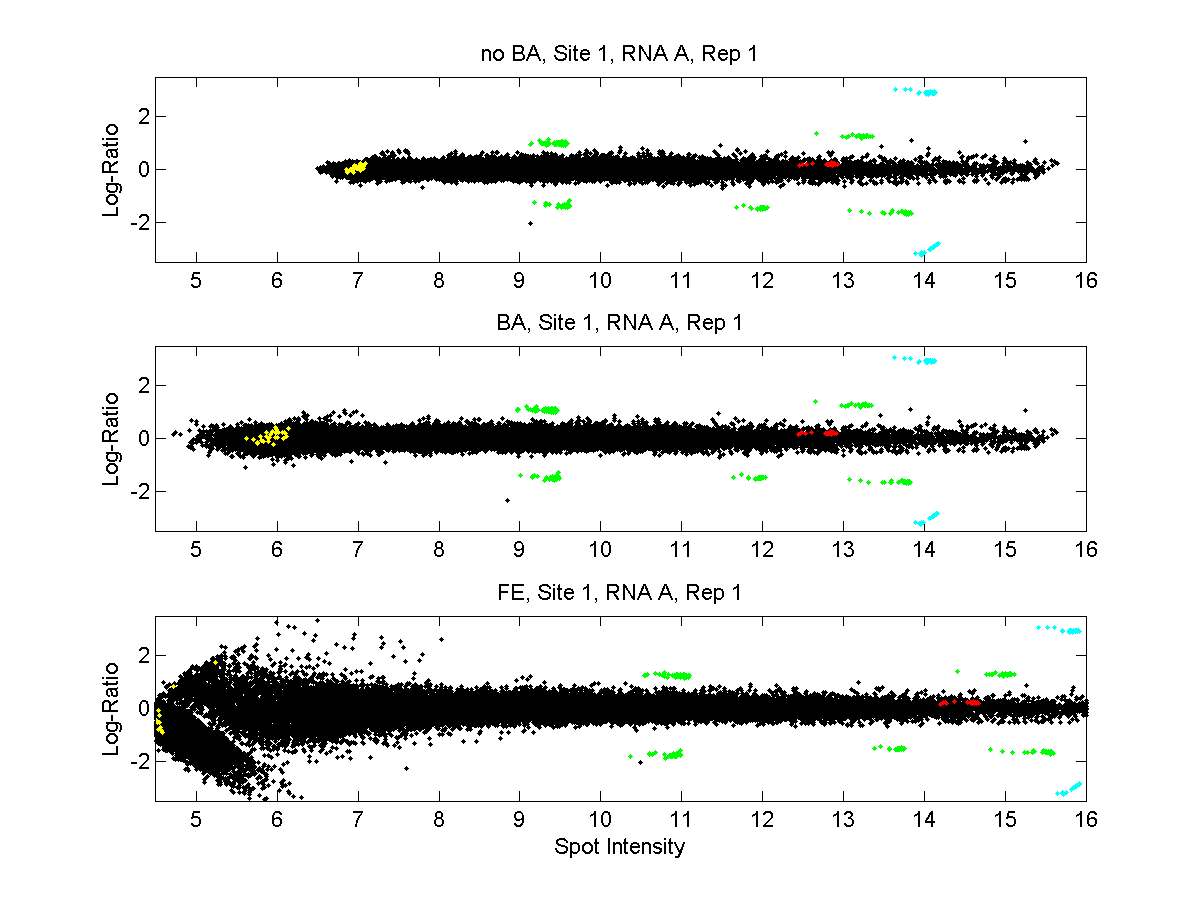

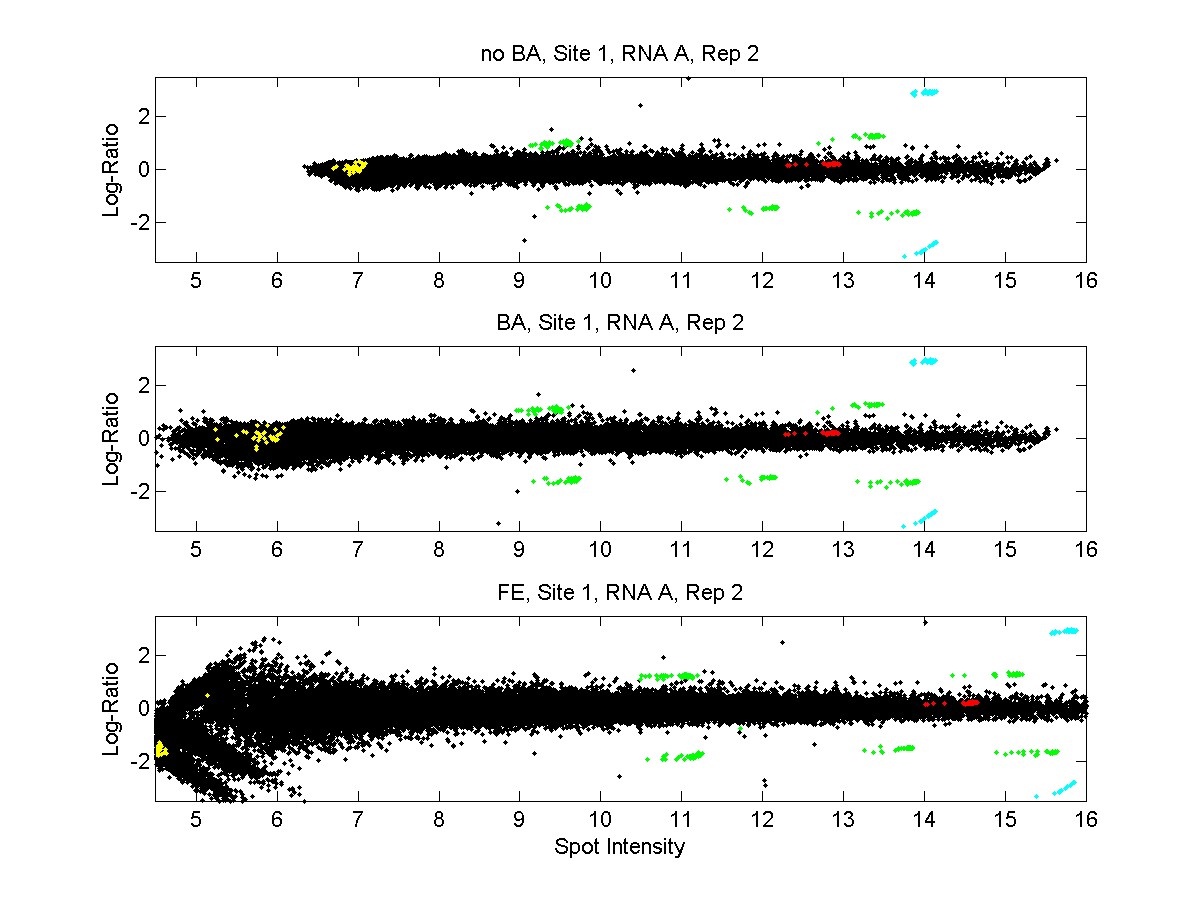


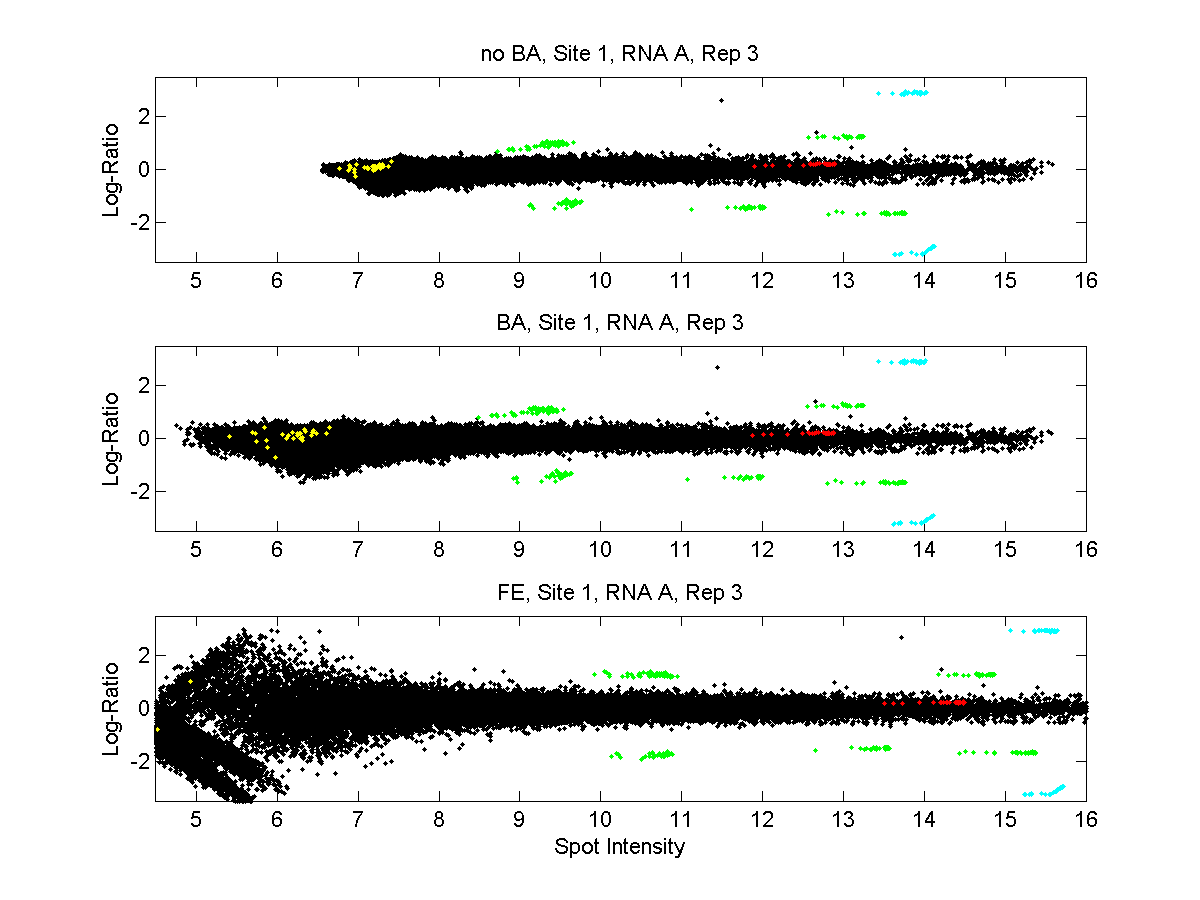

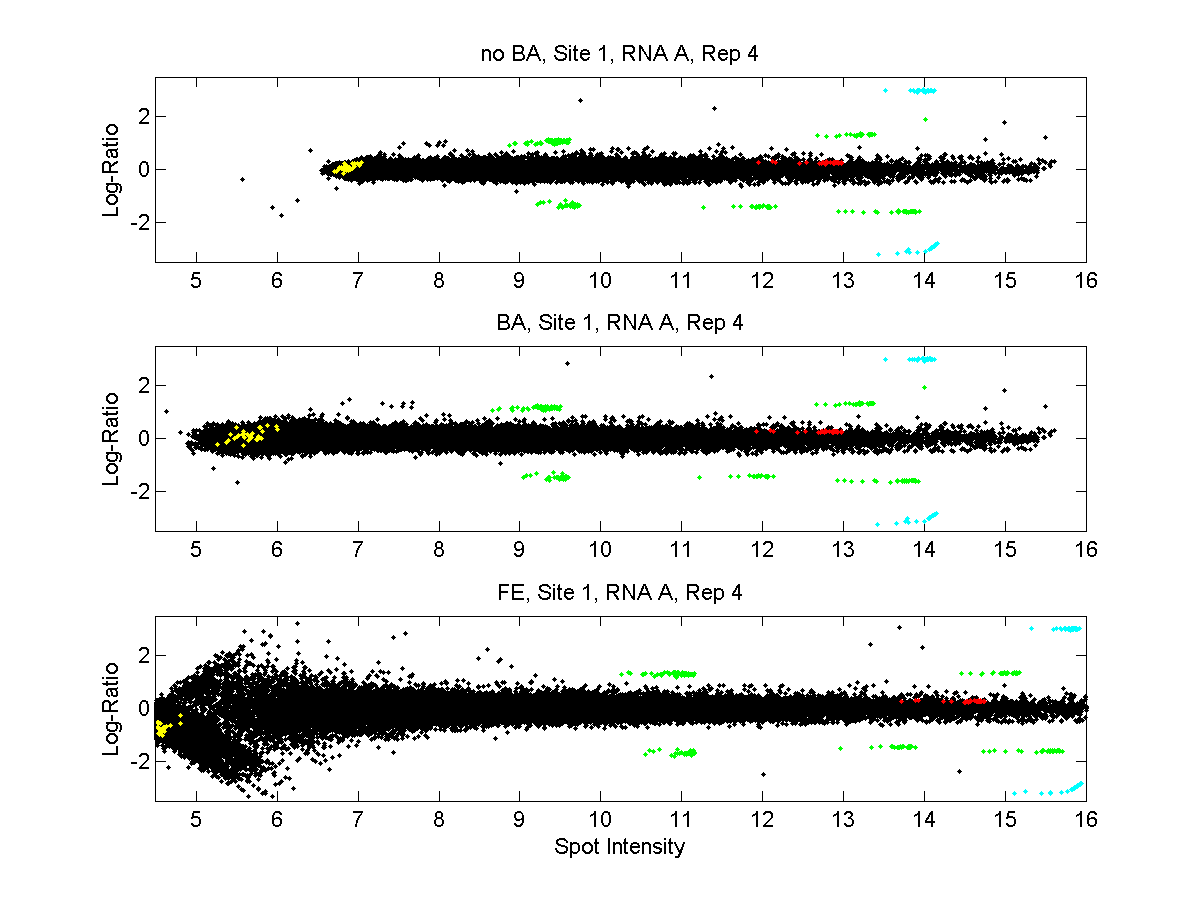


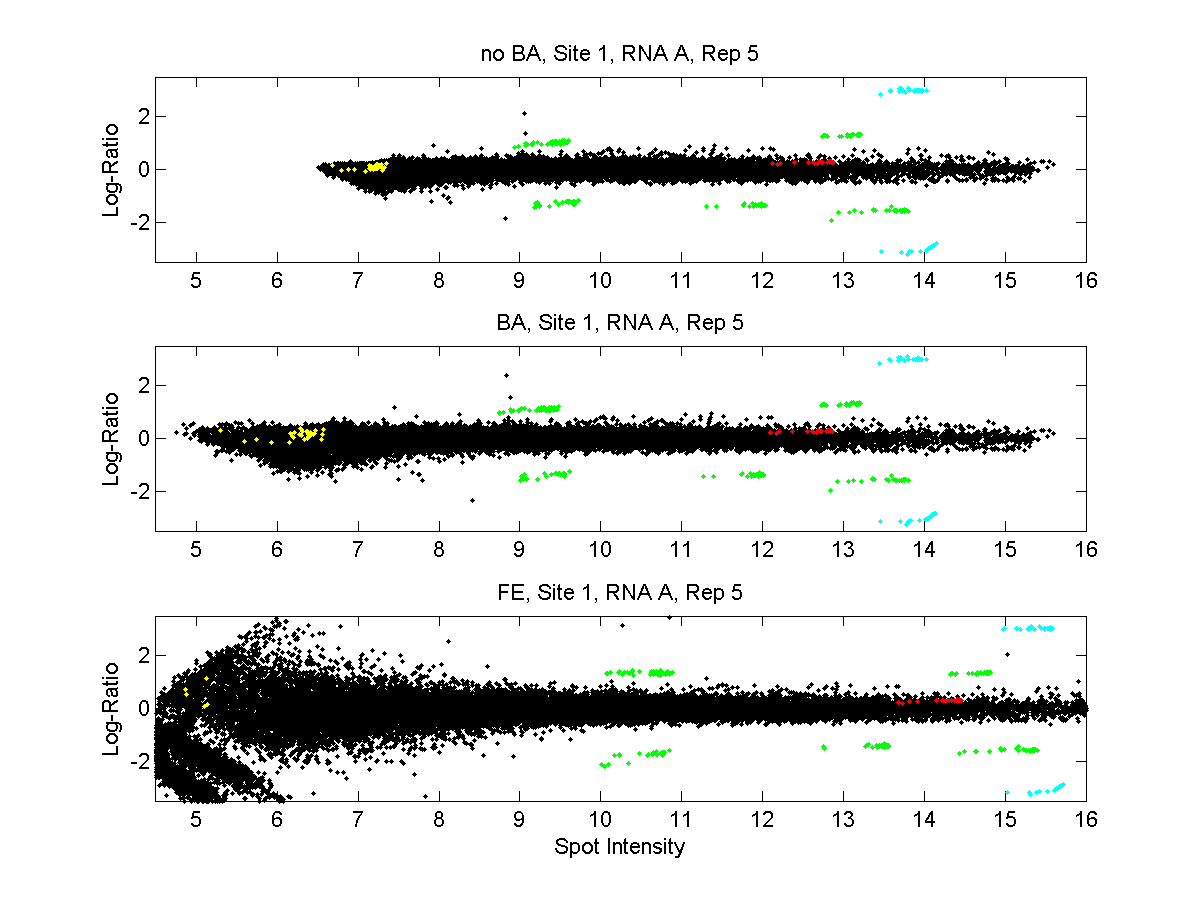


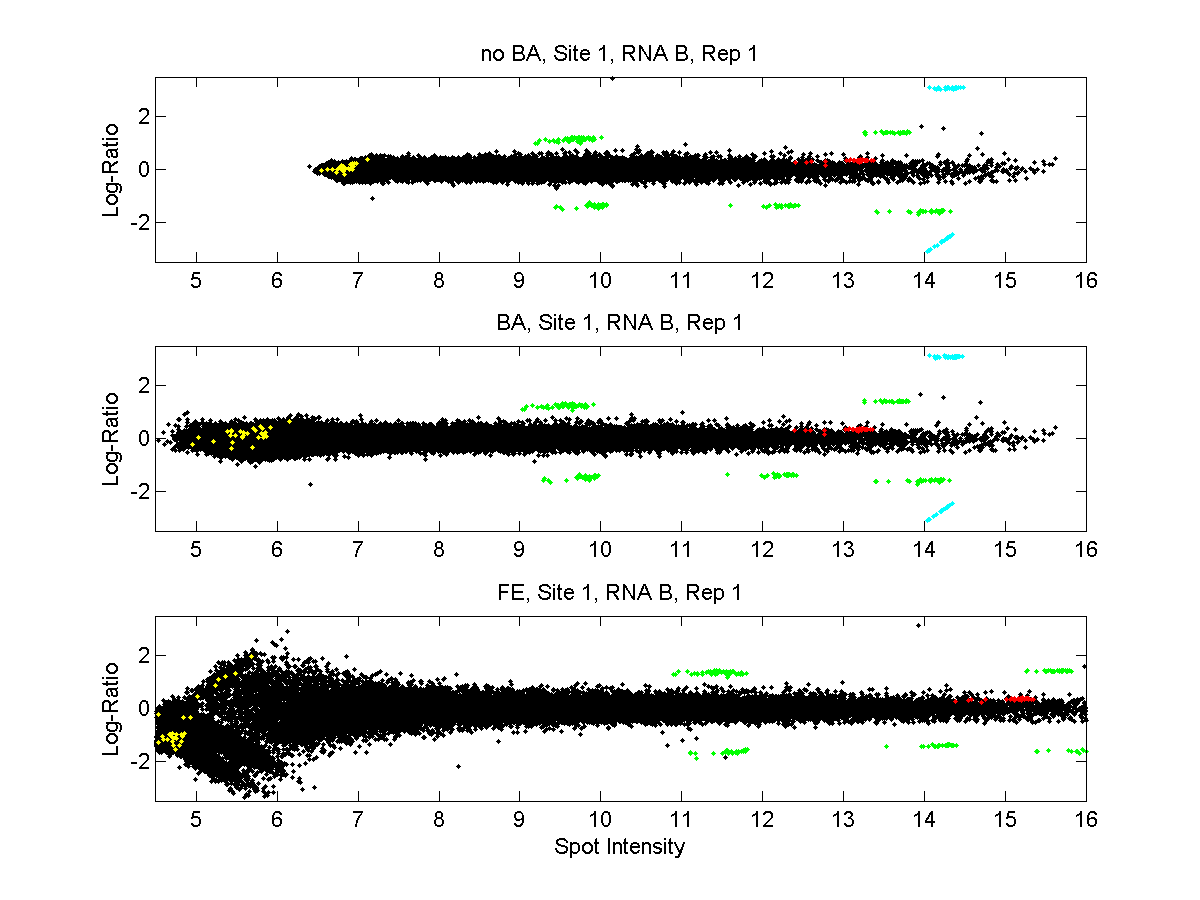

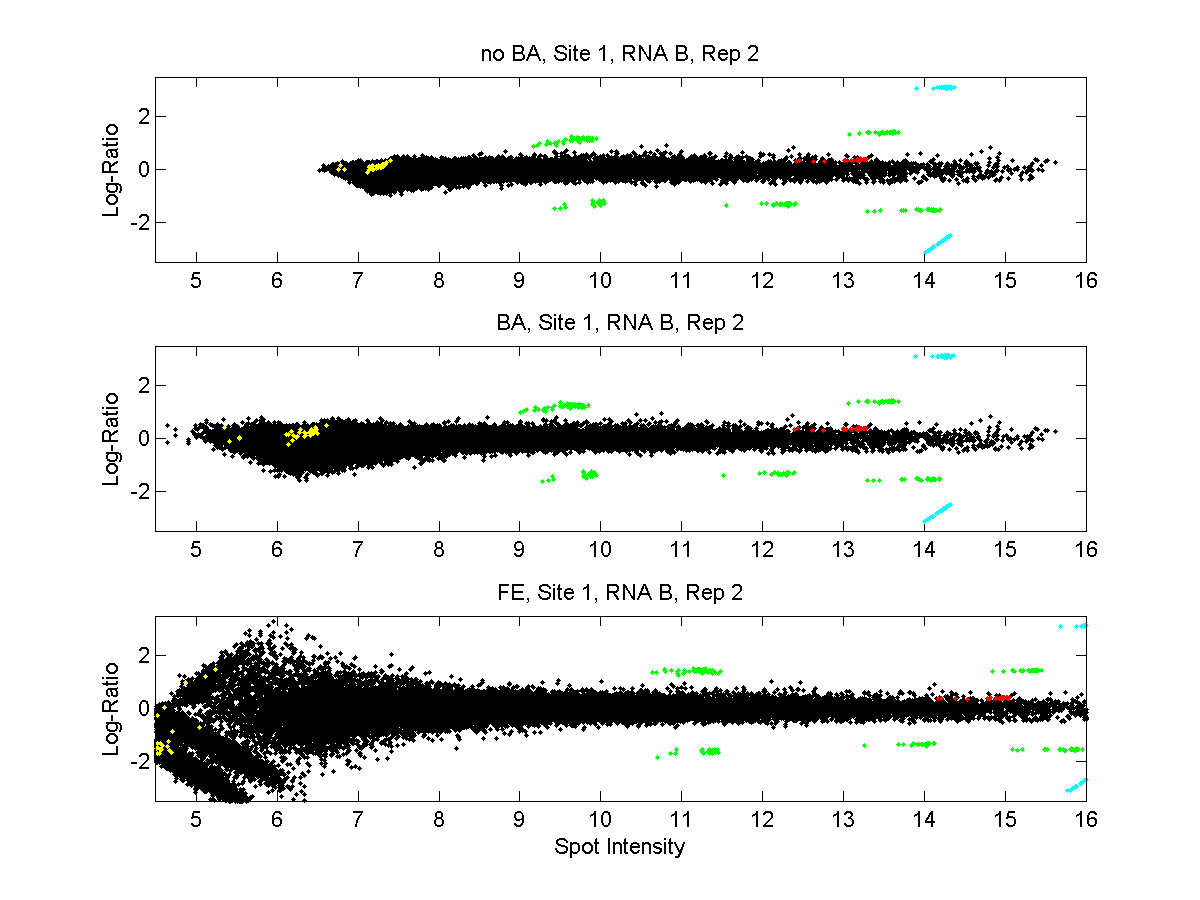

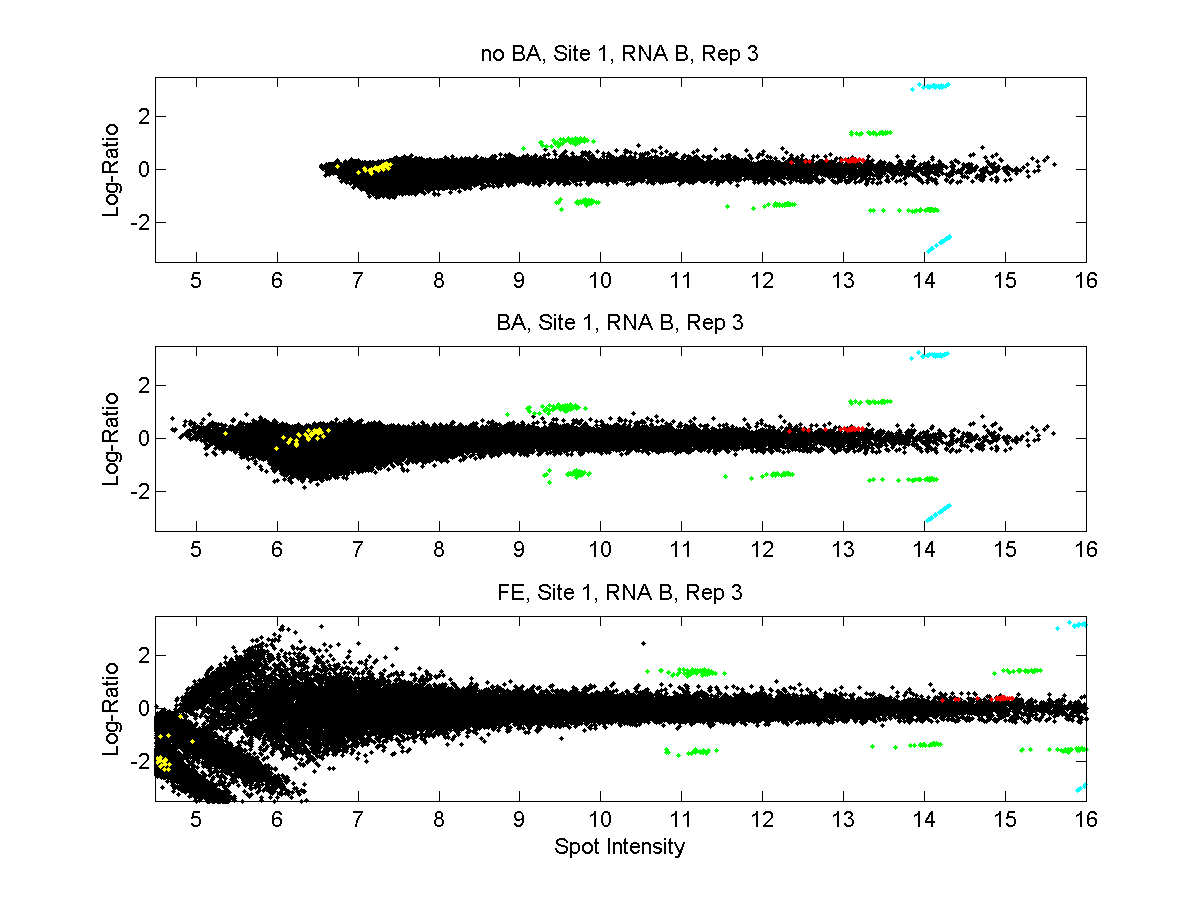


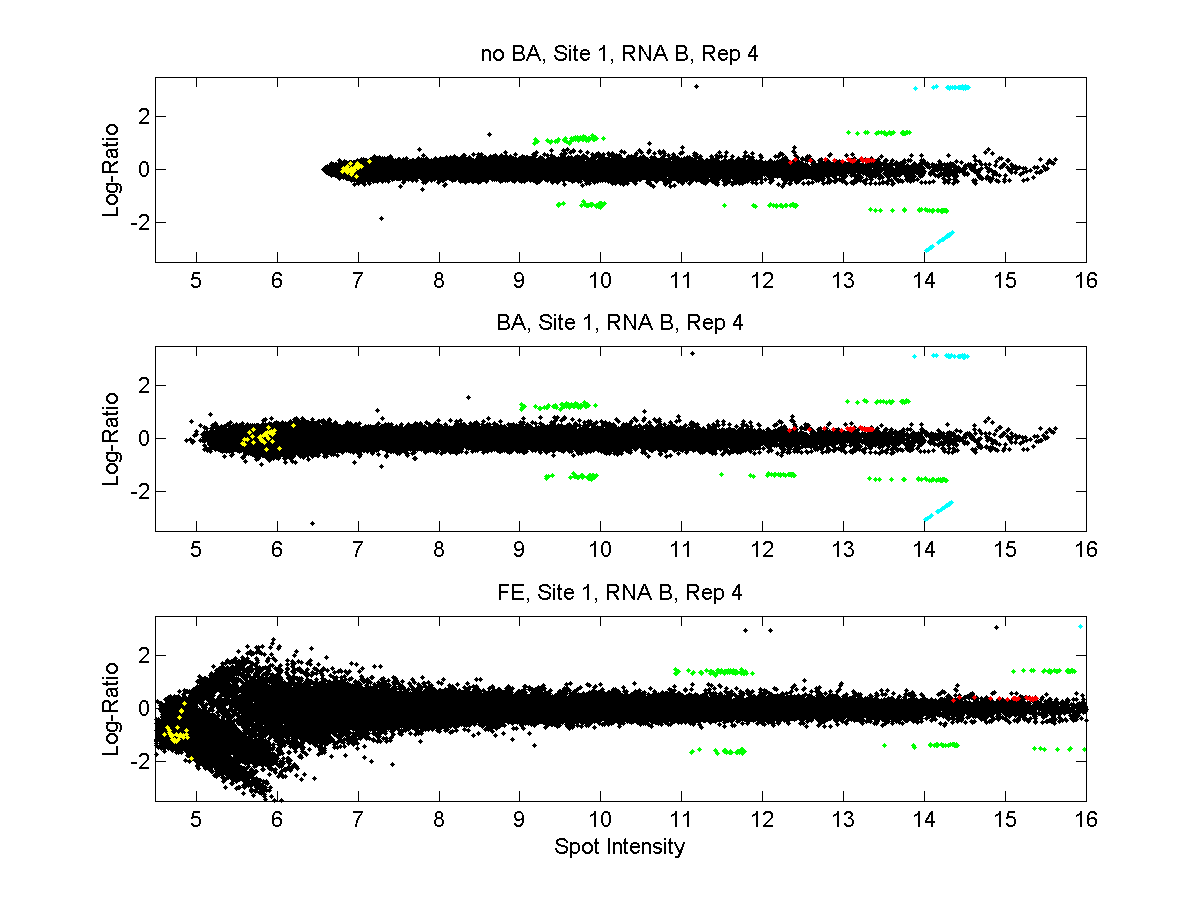


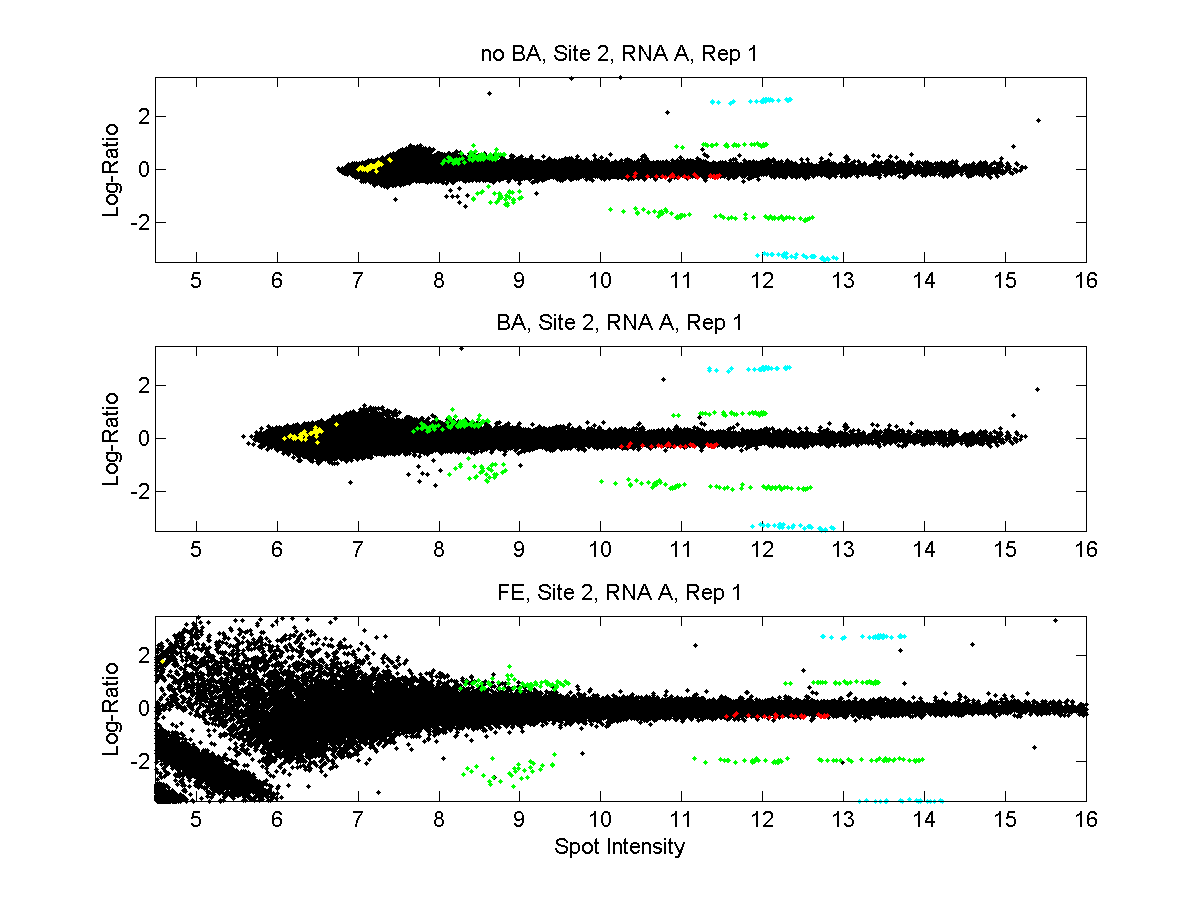

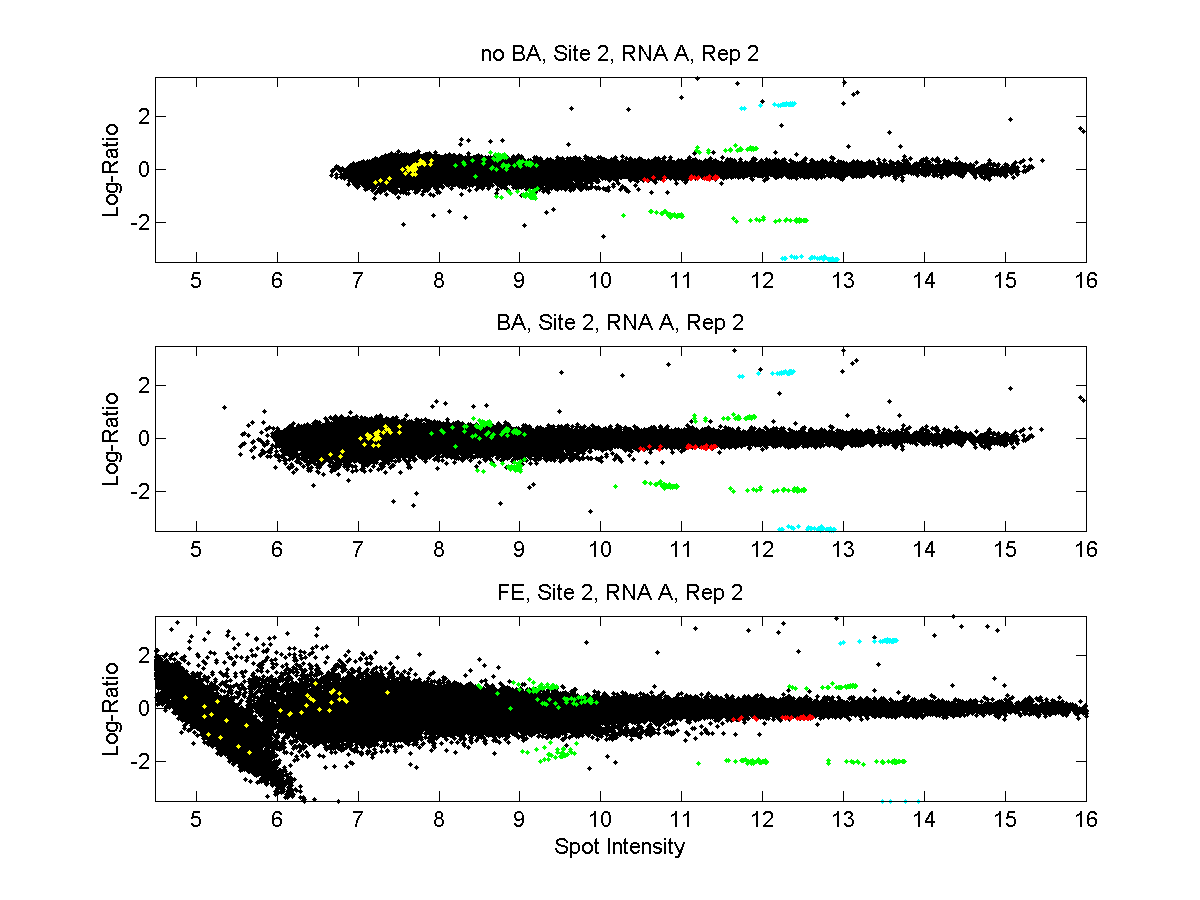

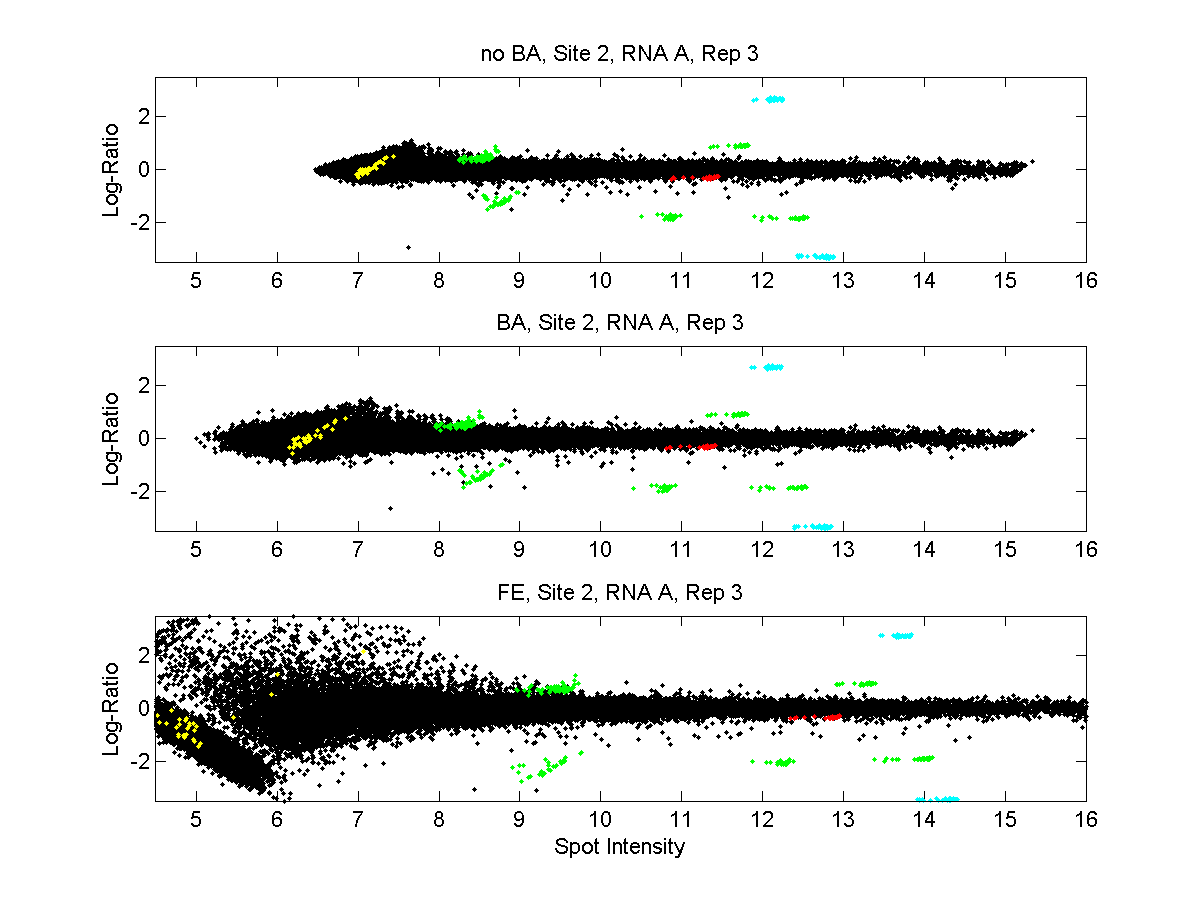


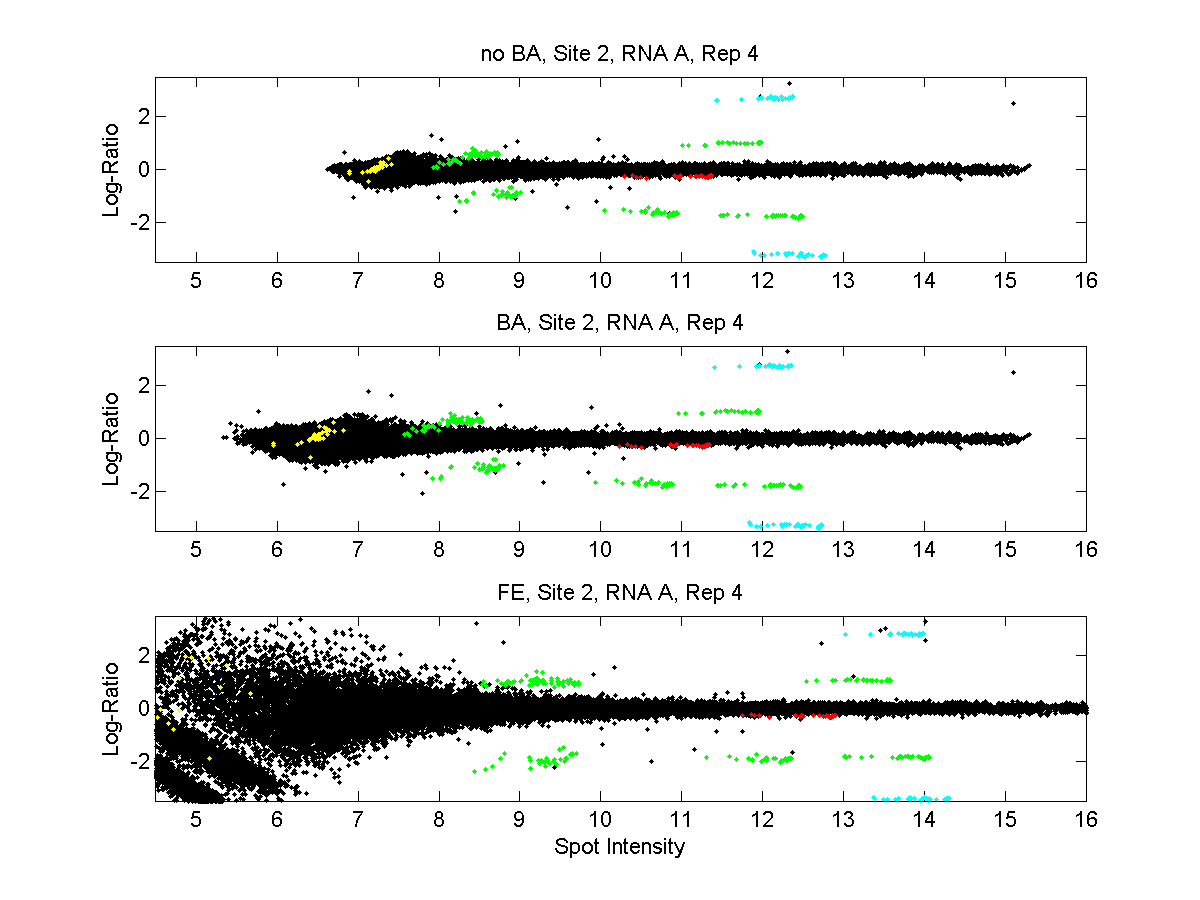


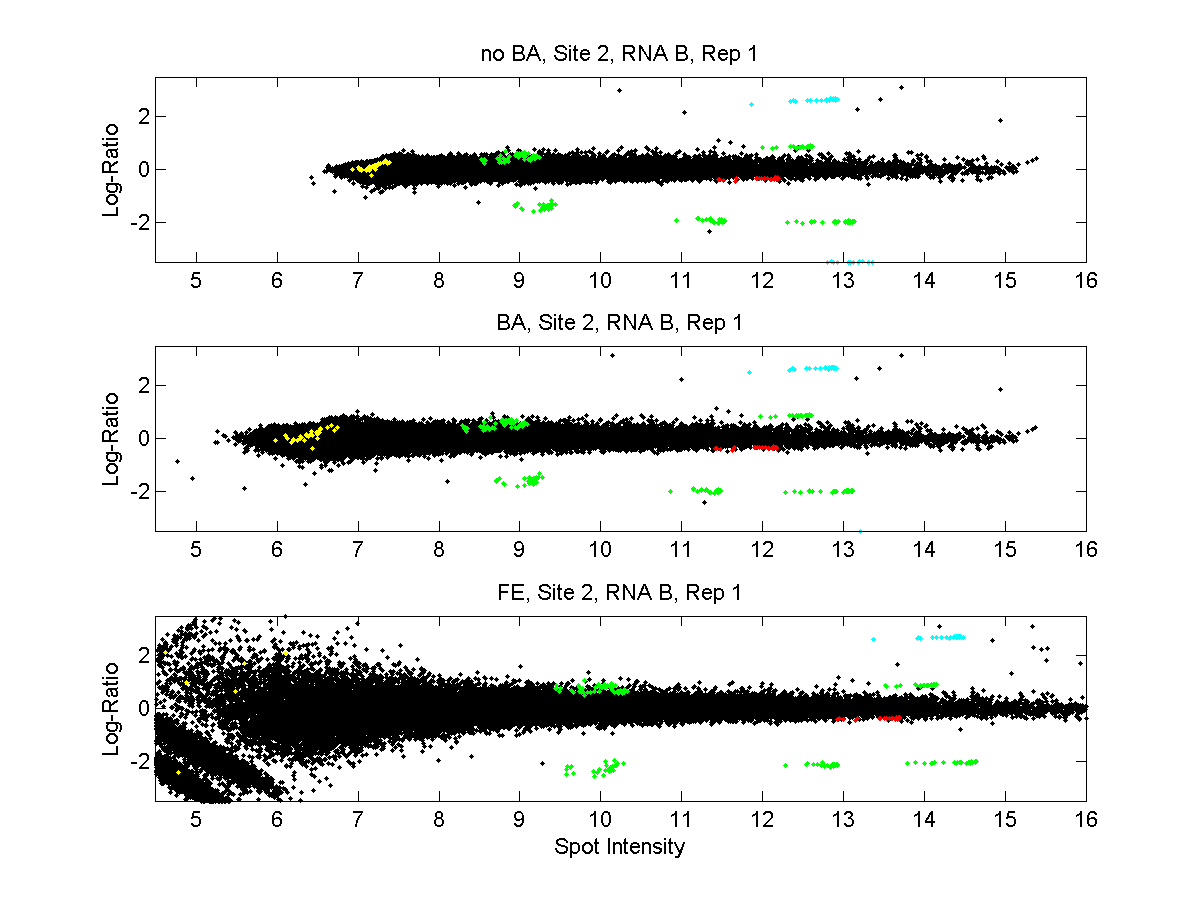

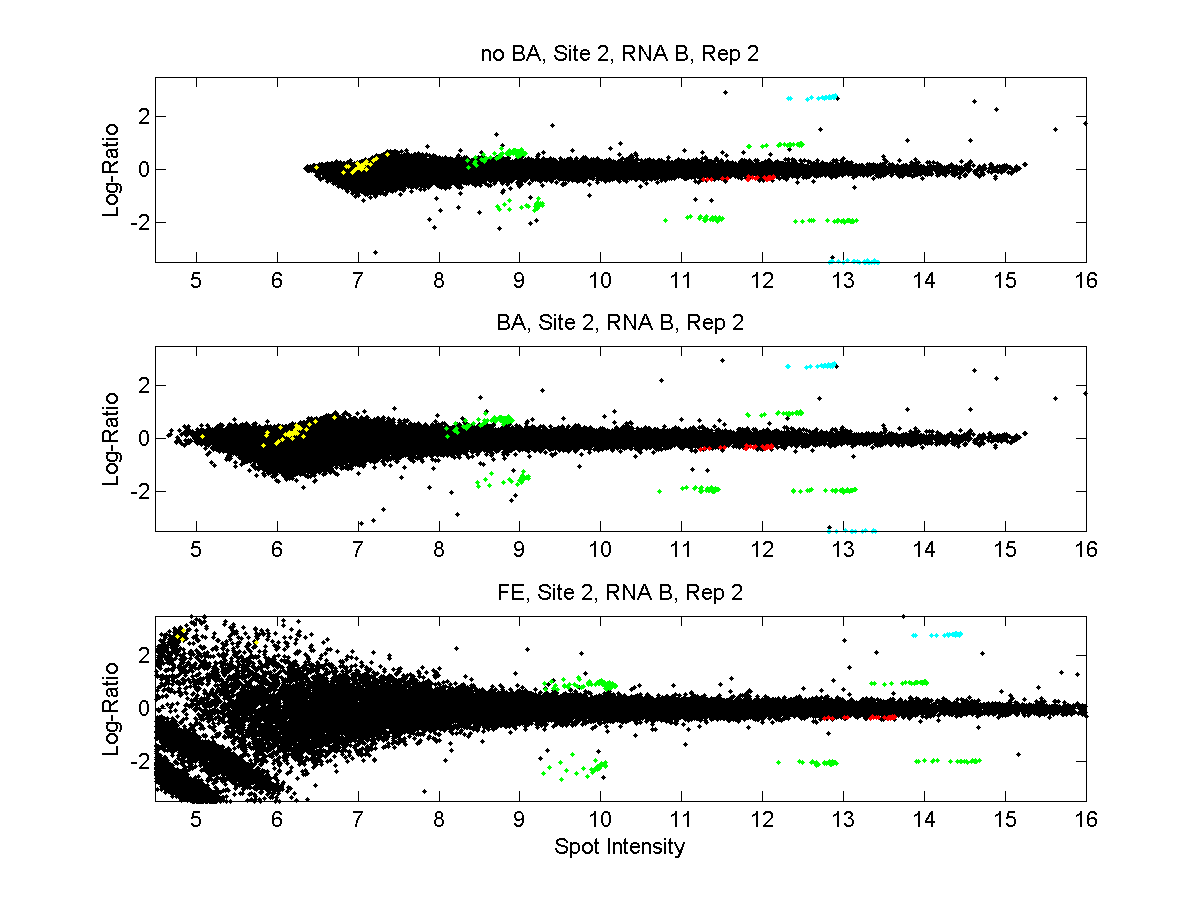

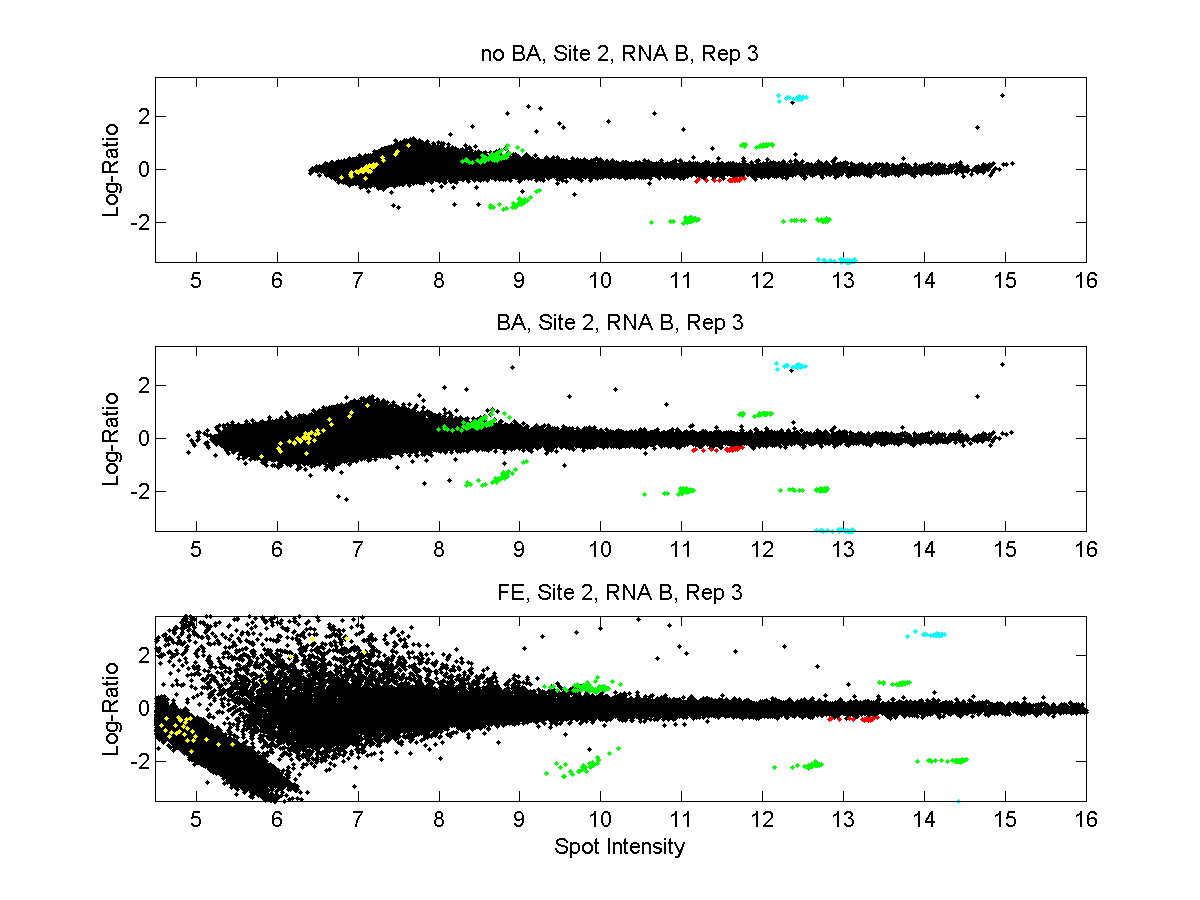

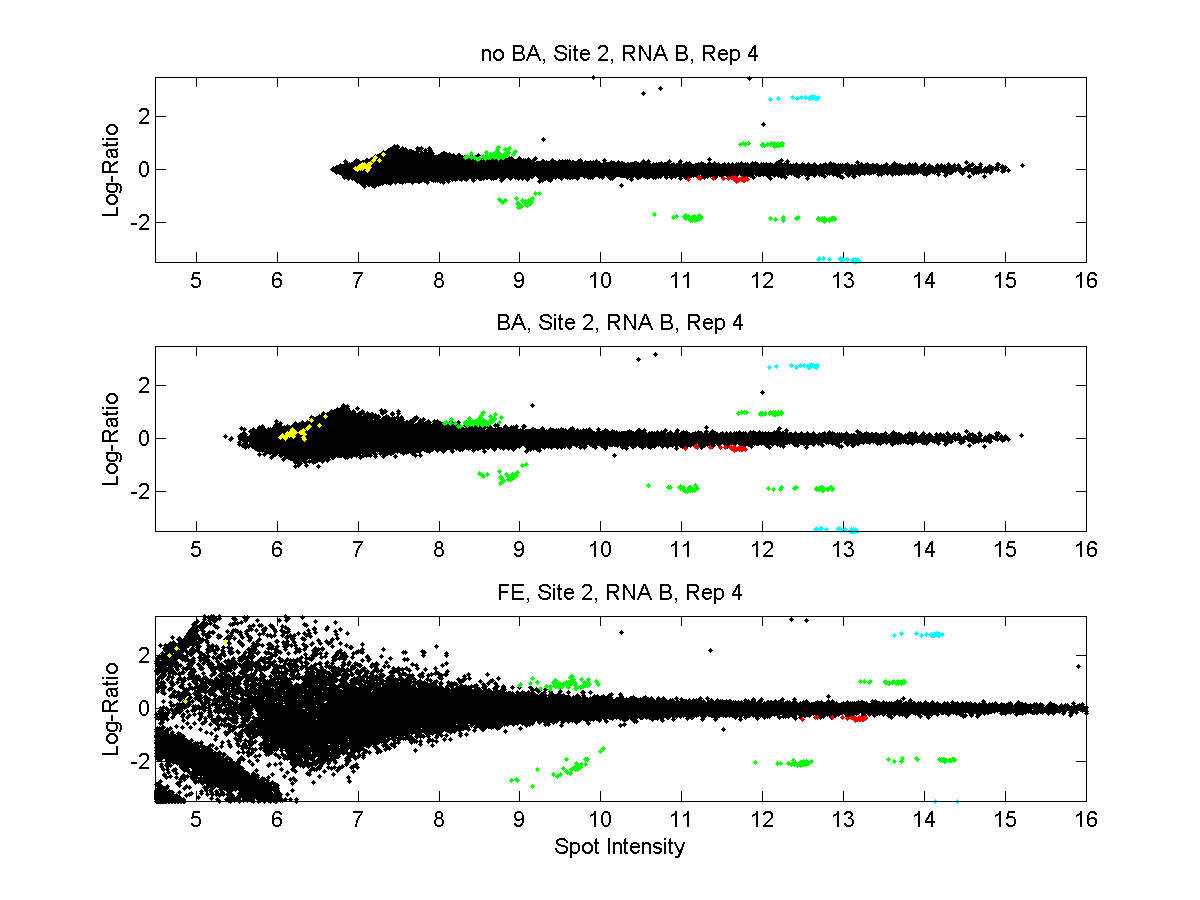


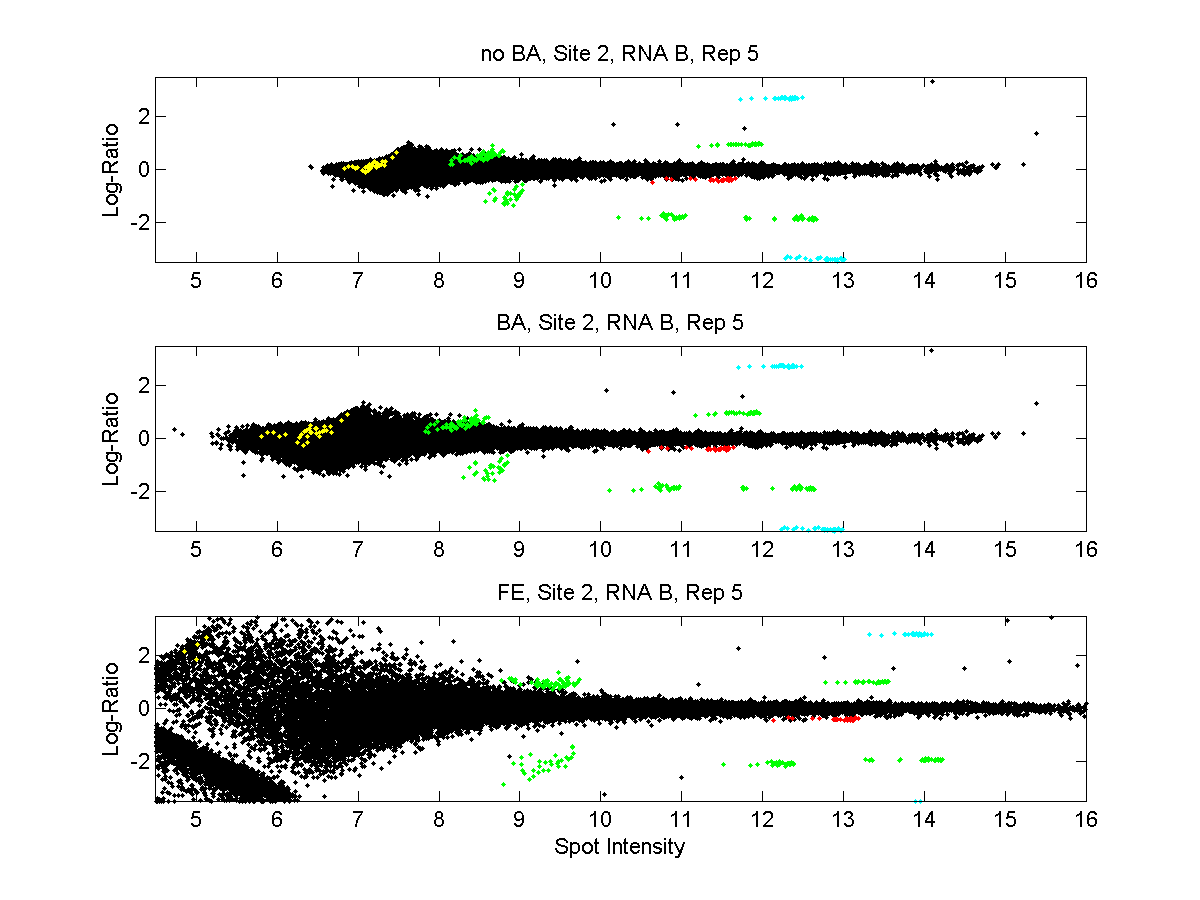


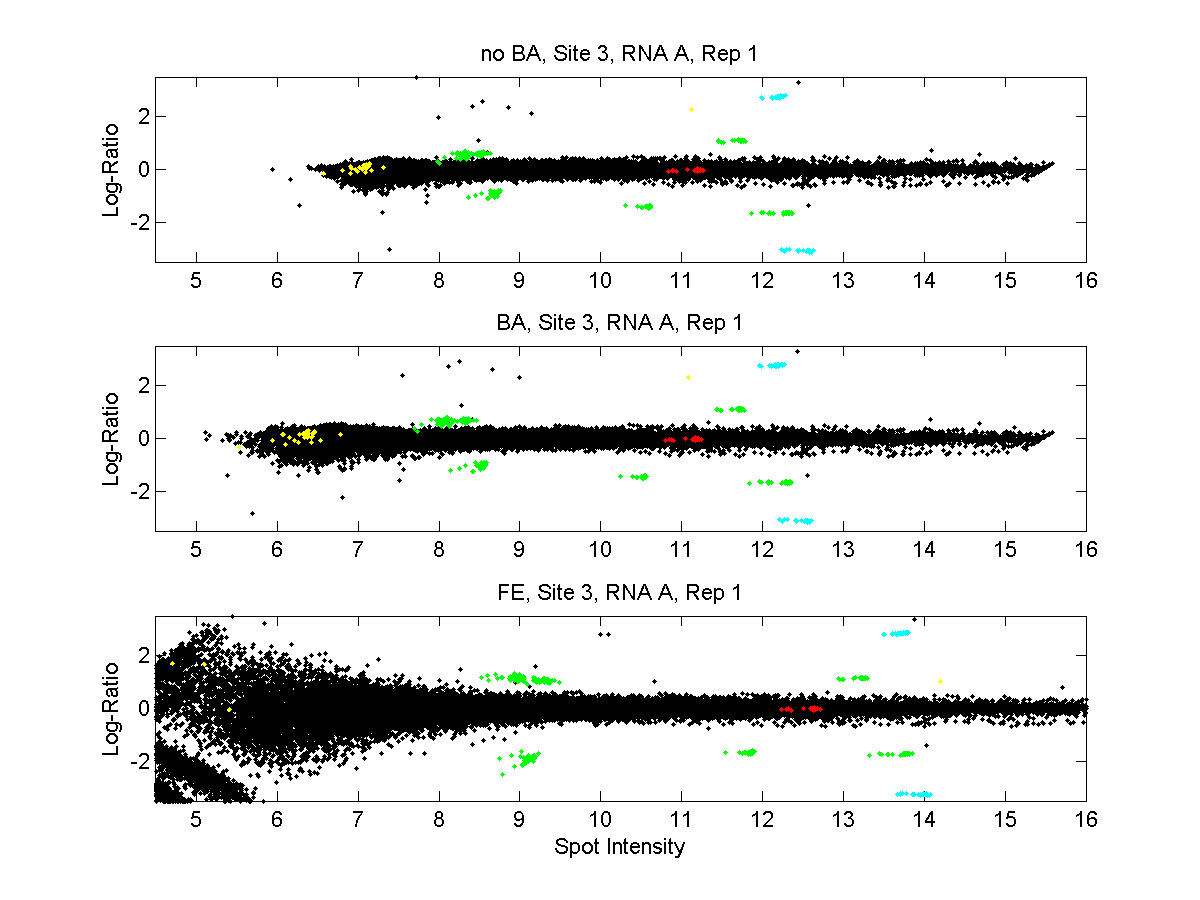

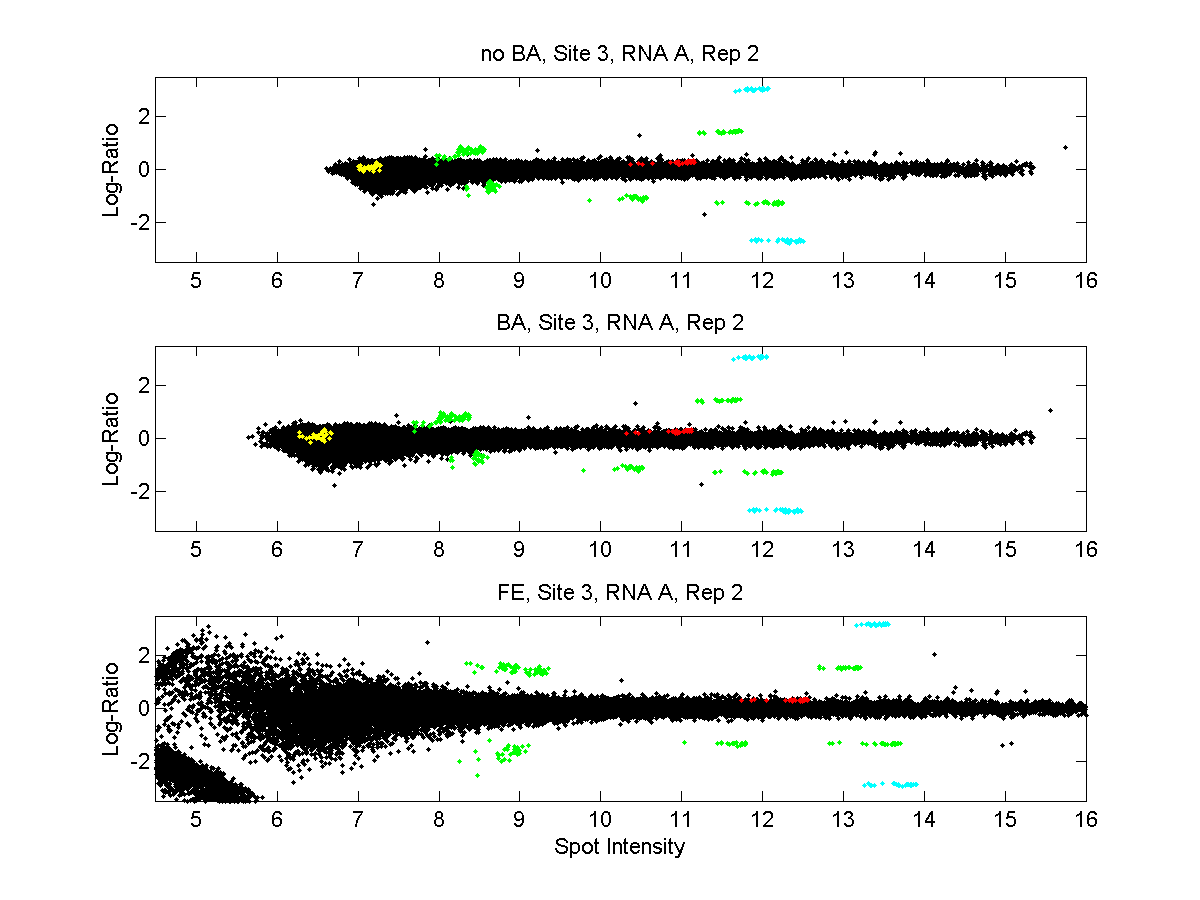

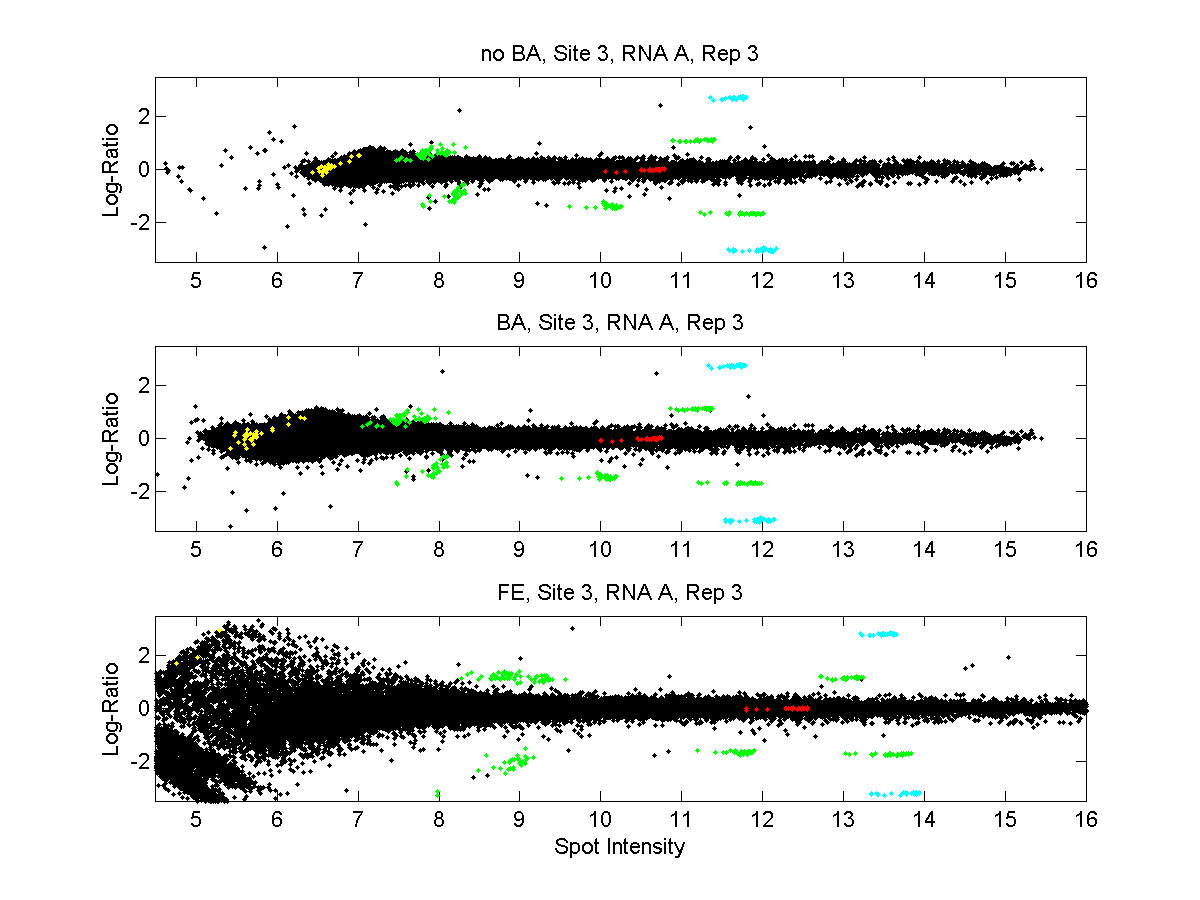

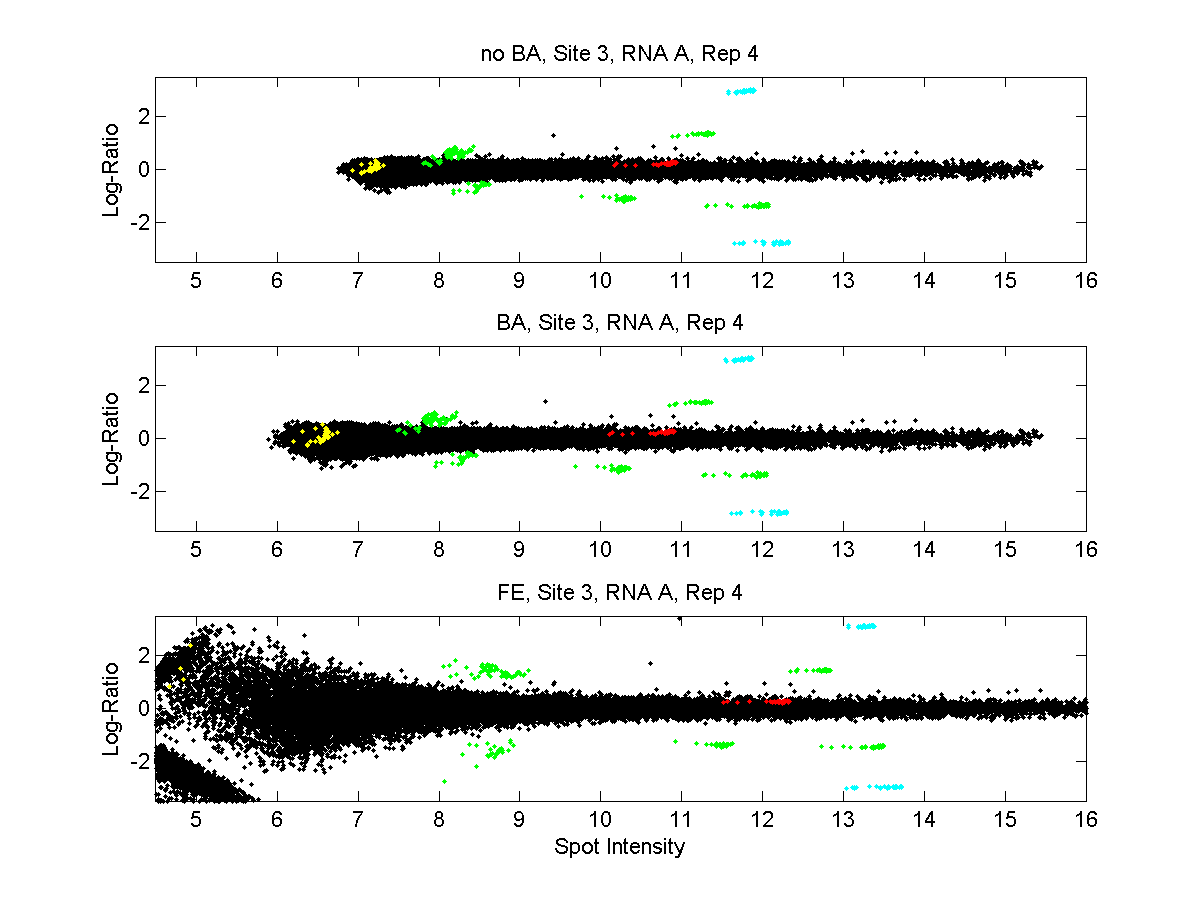

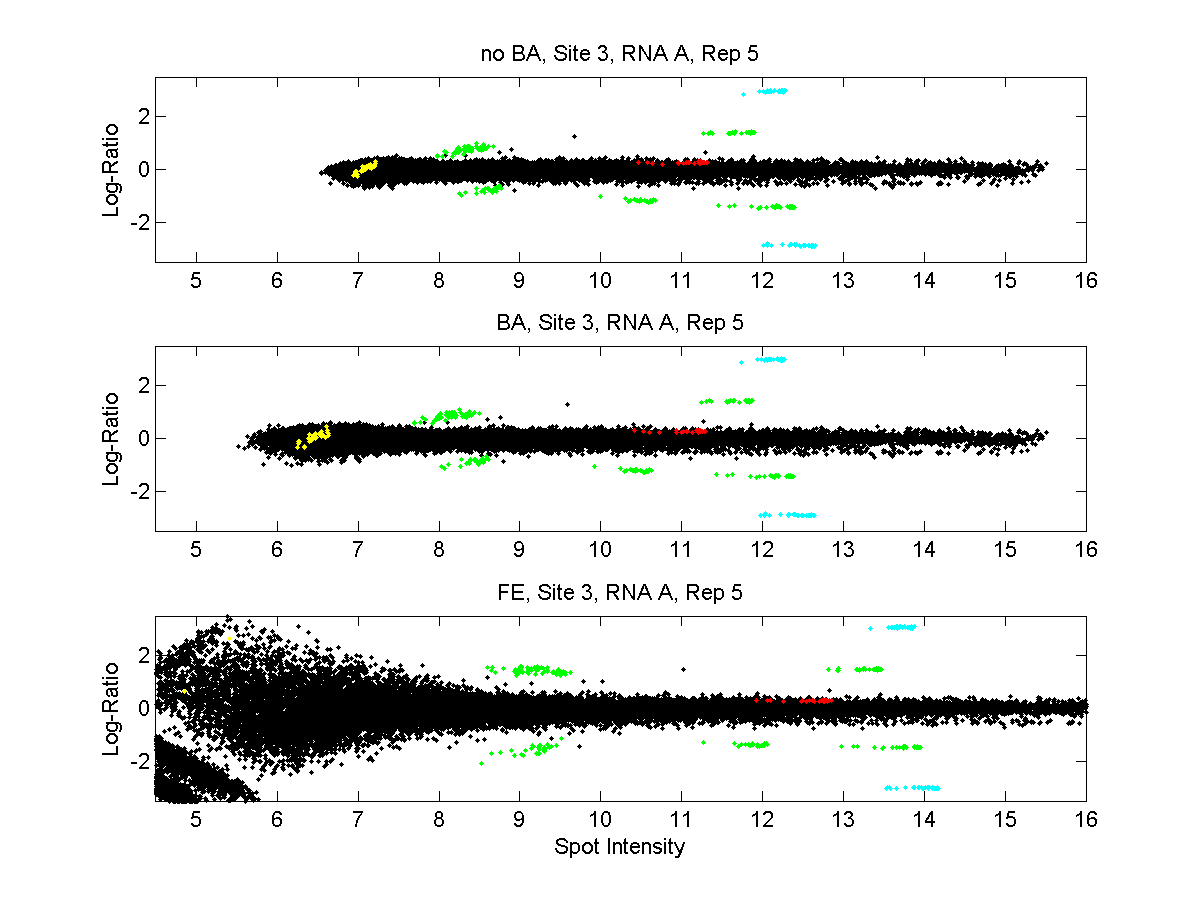

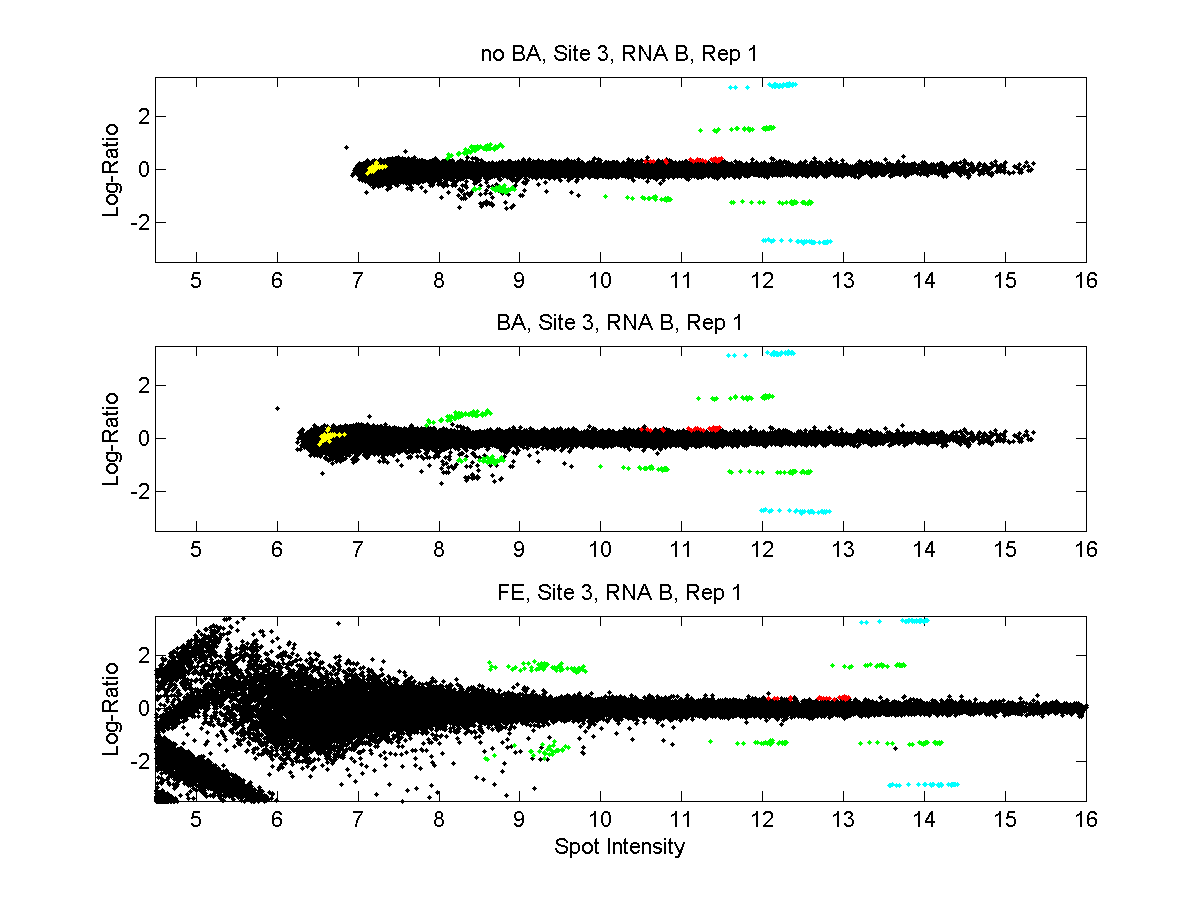

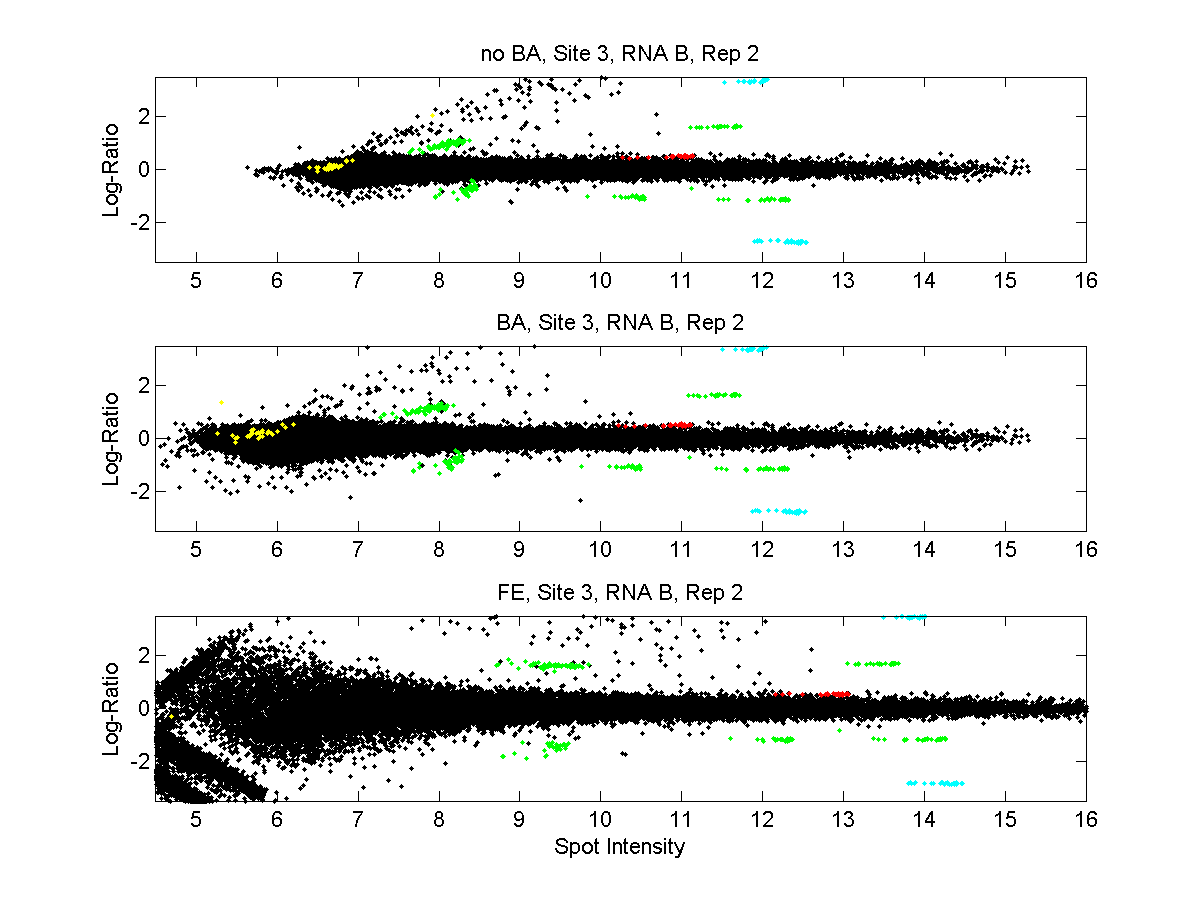

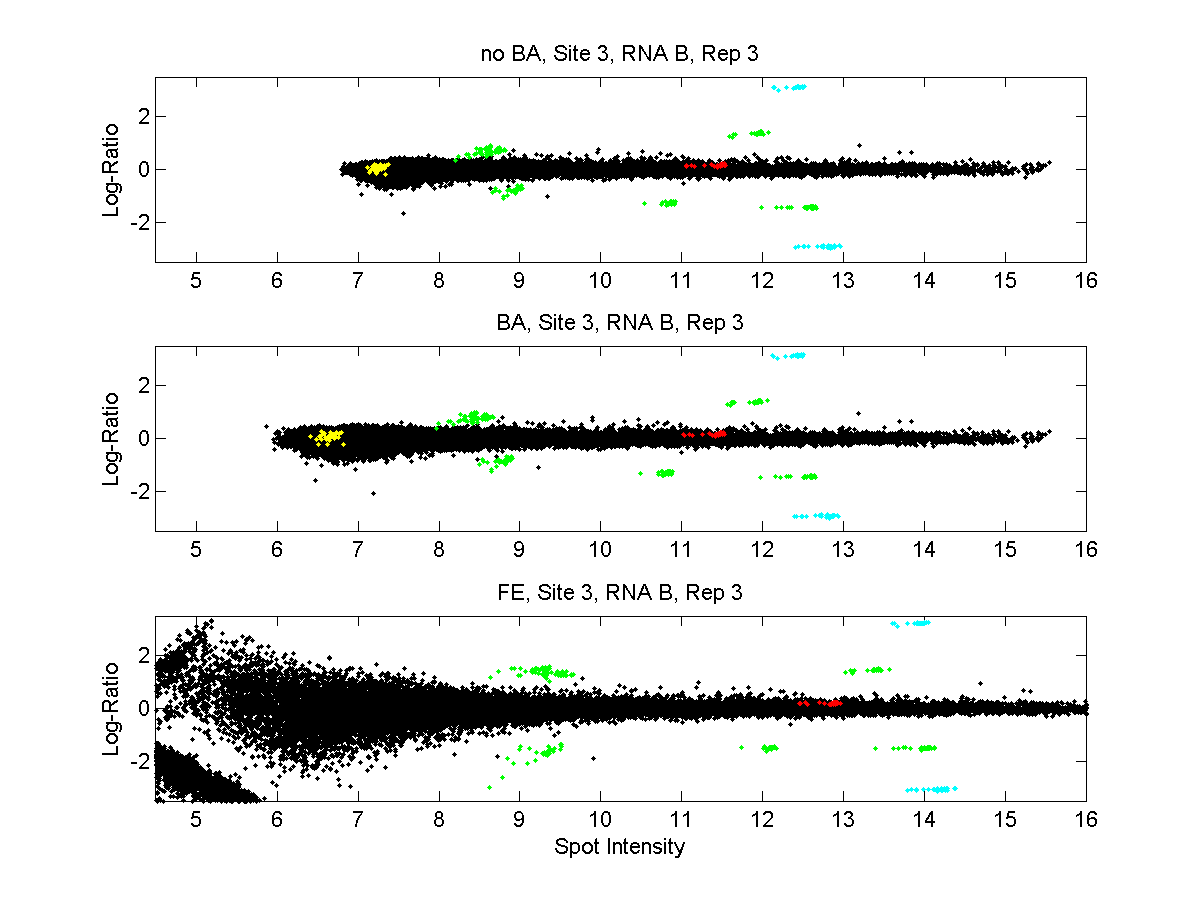

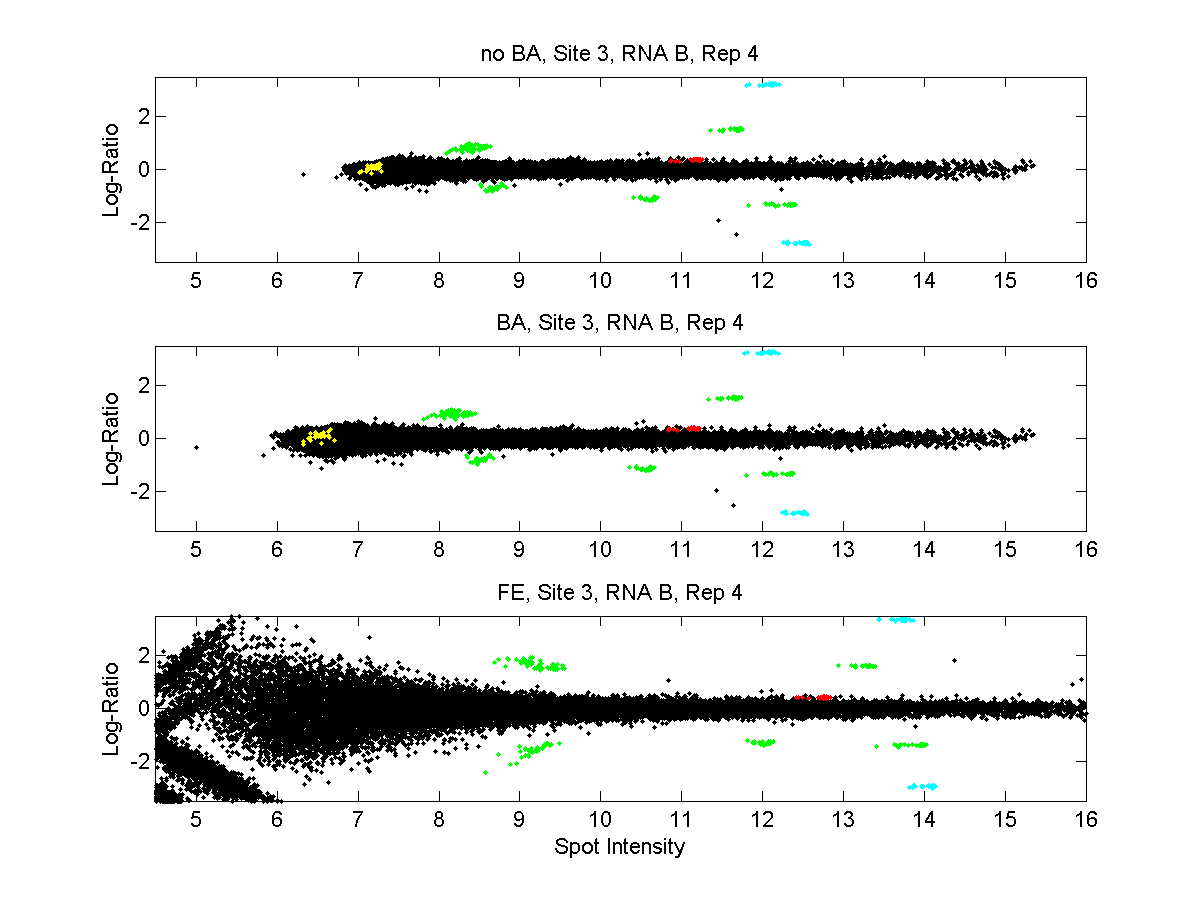


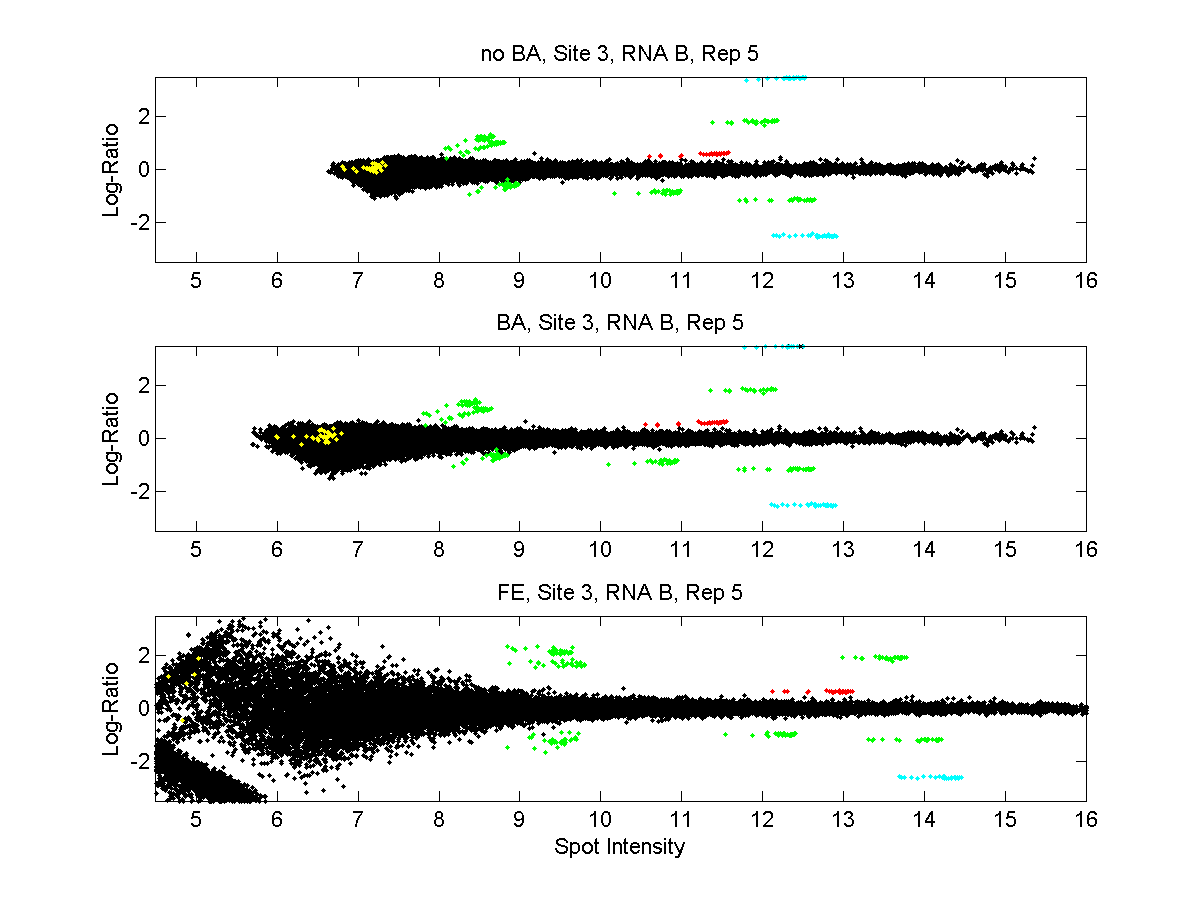

Supplement: Additional file 1 — Ratio-intensity plots for all arrays. The plots show log-ratios compared to signal intensity for three versions of the data. [file 1471-2105-8-371-S1.doc]
