# Supplementary figures and images for: Extended analysis of benchmark datasets for Agilent two-color microarrays
Source: BMC Bioinformatics. 2007 Oct 3;8:371. doi: 10.1186/1471-2105-8-371 (PMC2174956; doi:10.1186/1471-2105-8-371)

Supplement 2. ROC curves for the mean, t-statistic, and SAM statistic applied to six datasets.


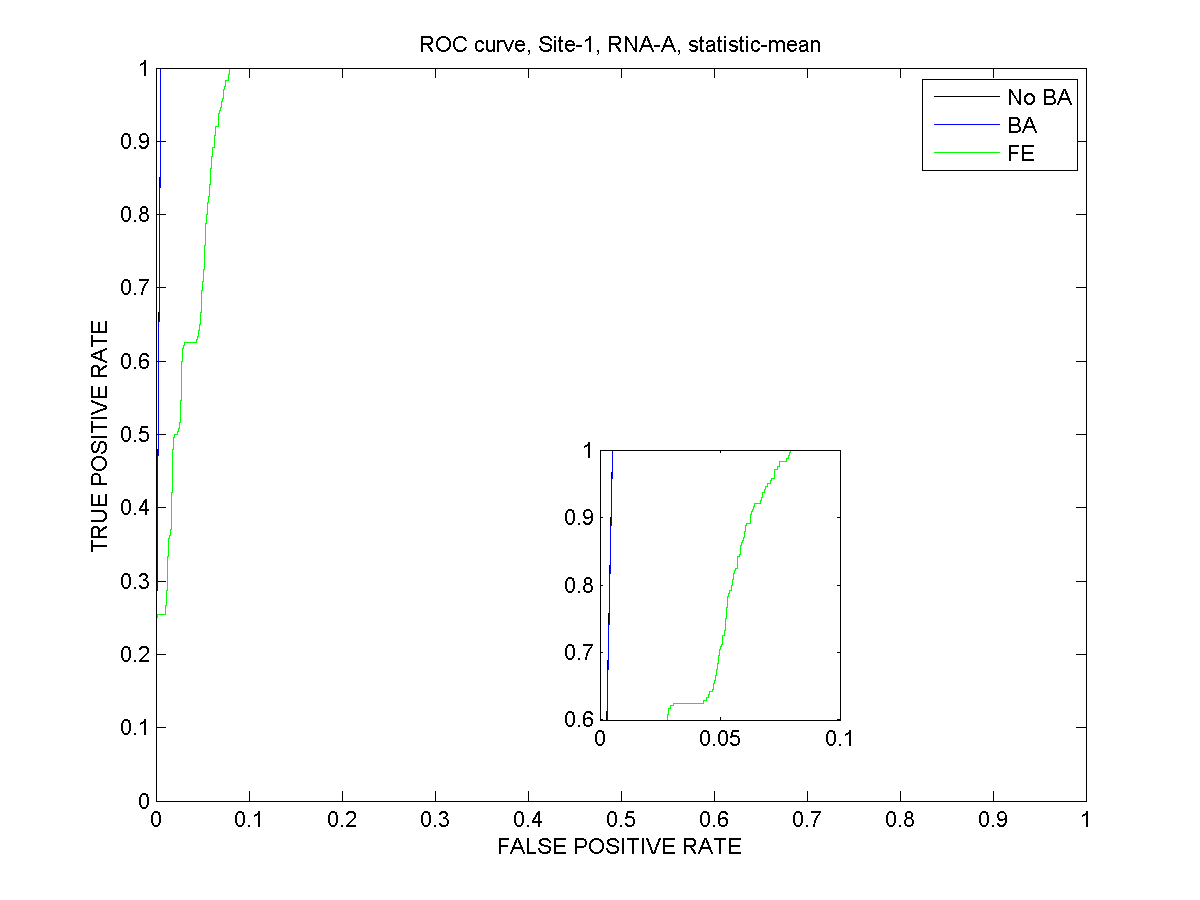

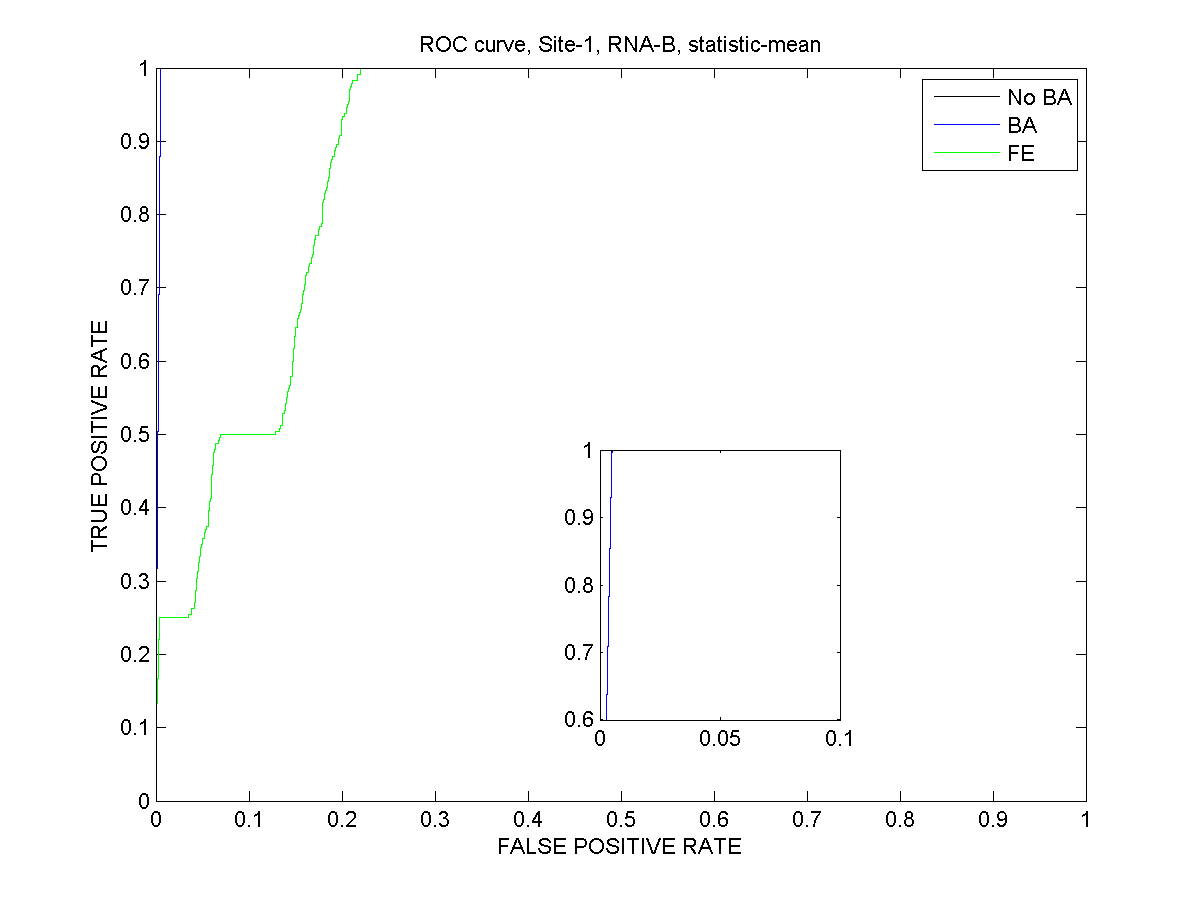

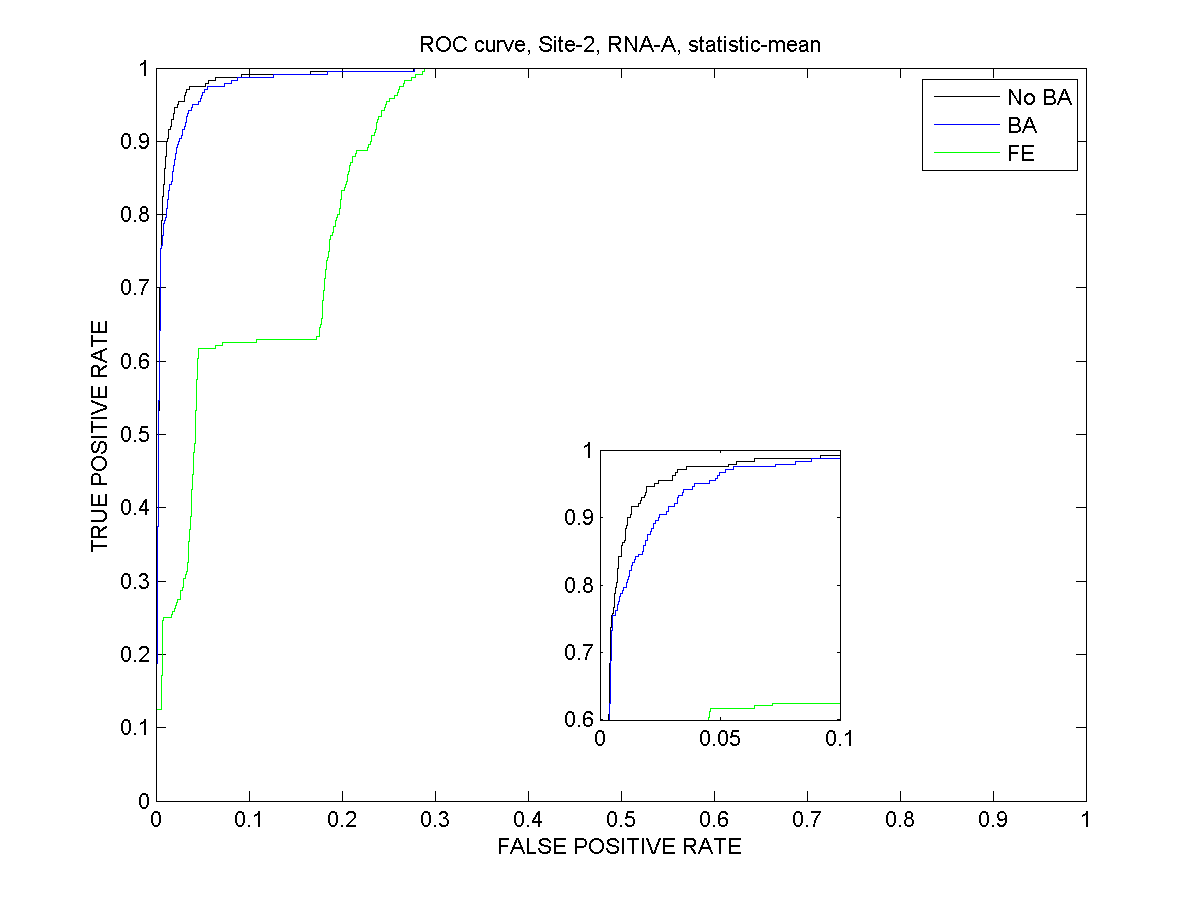

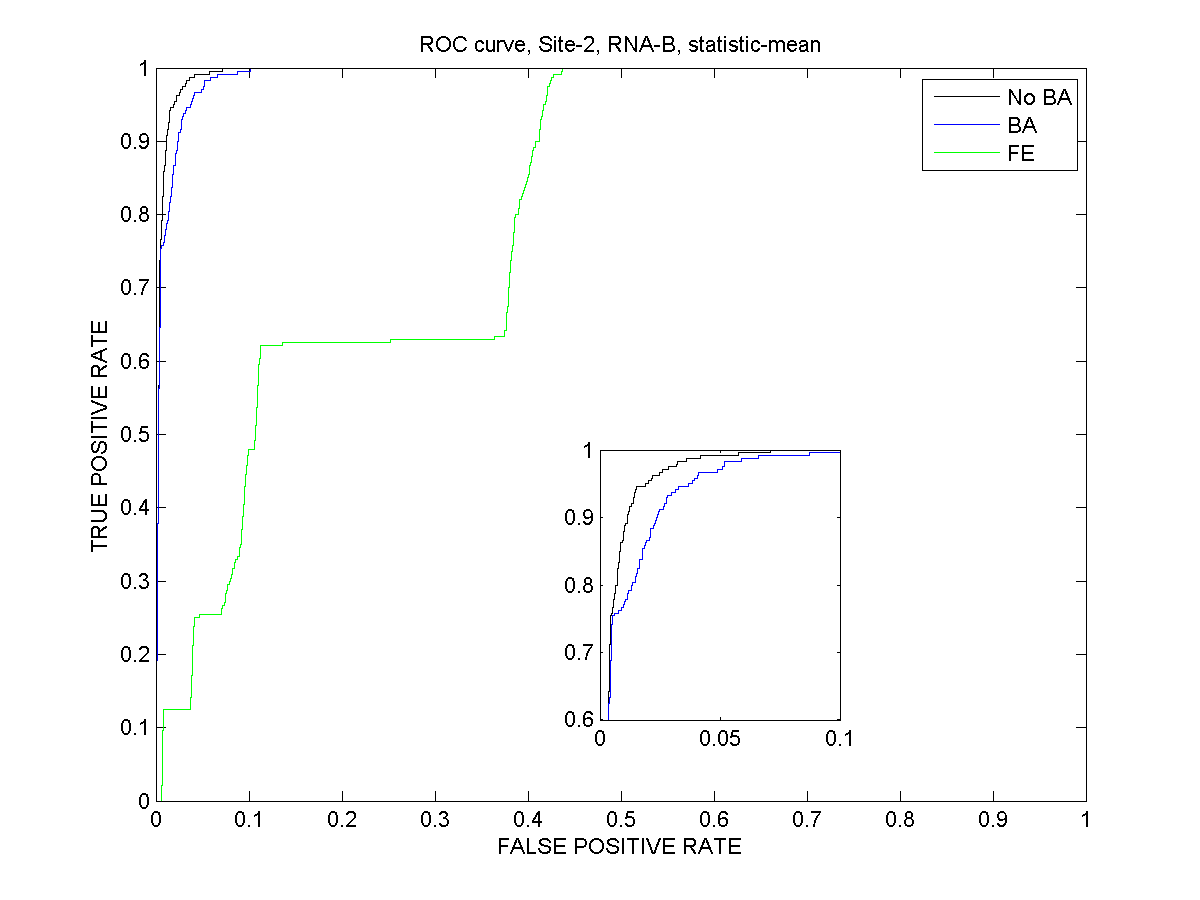

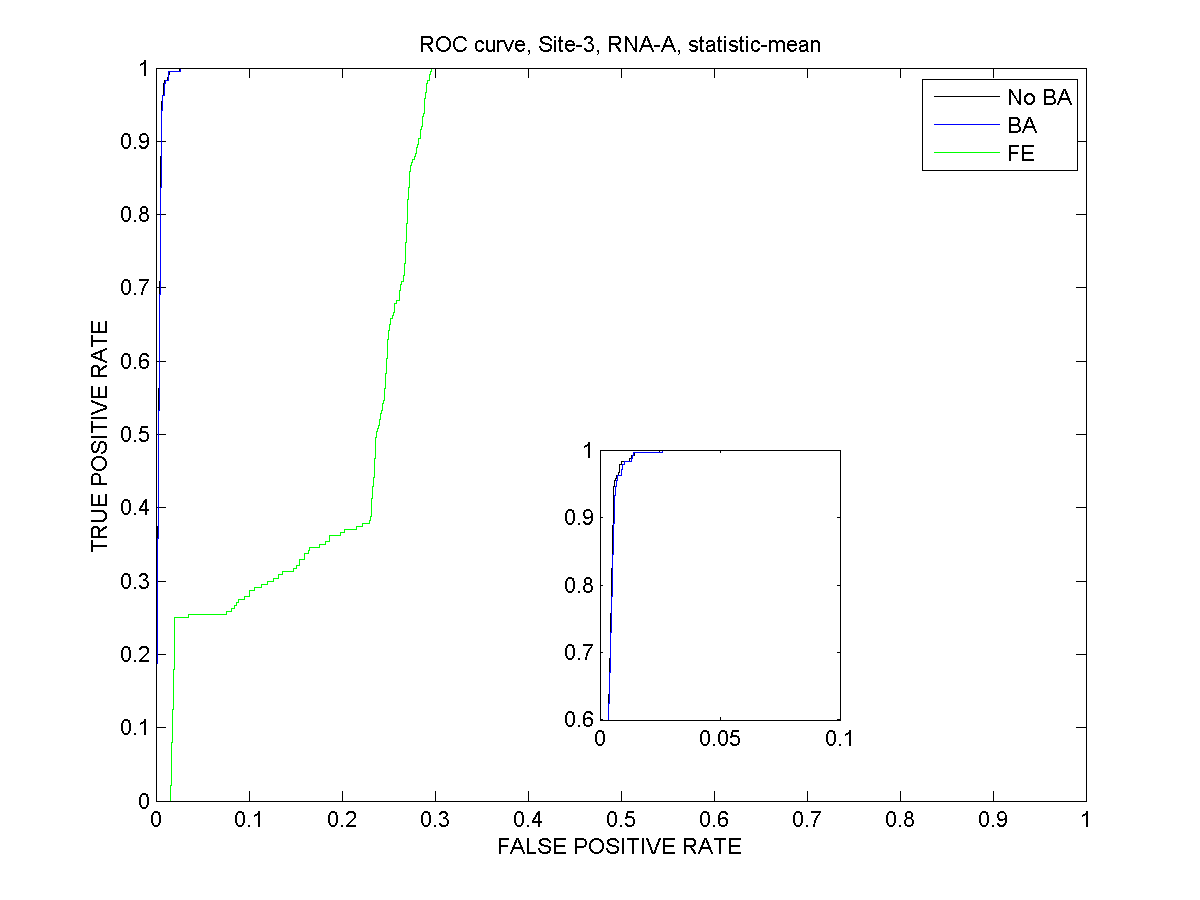

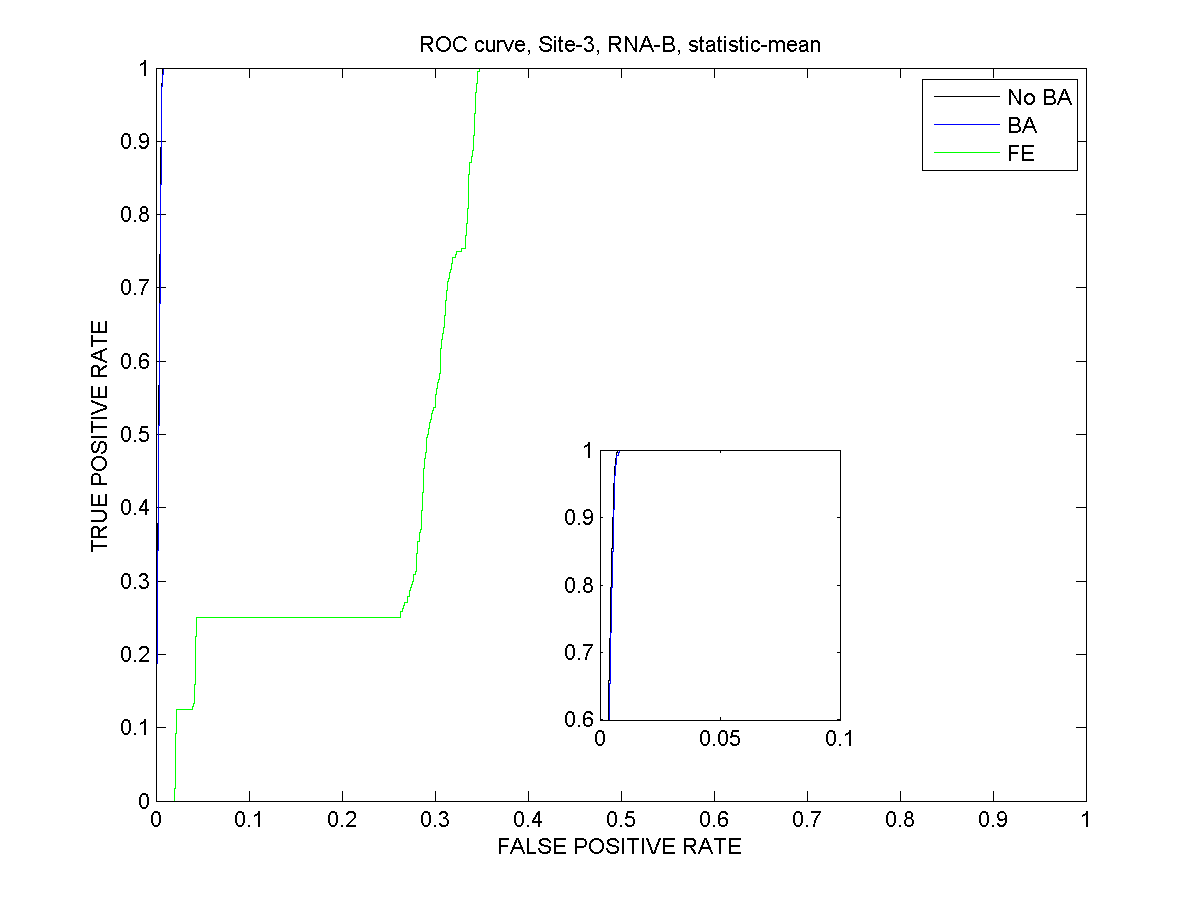

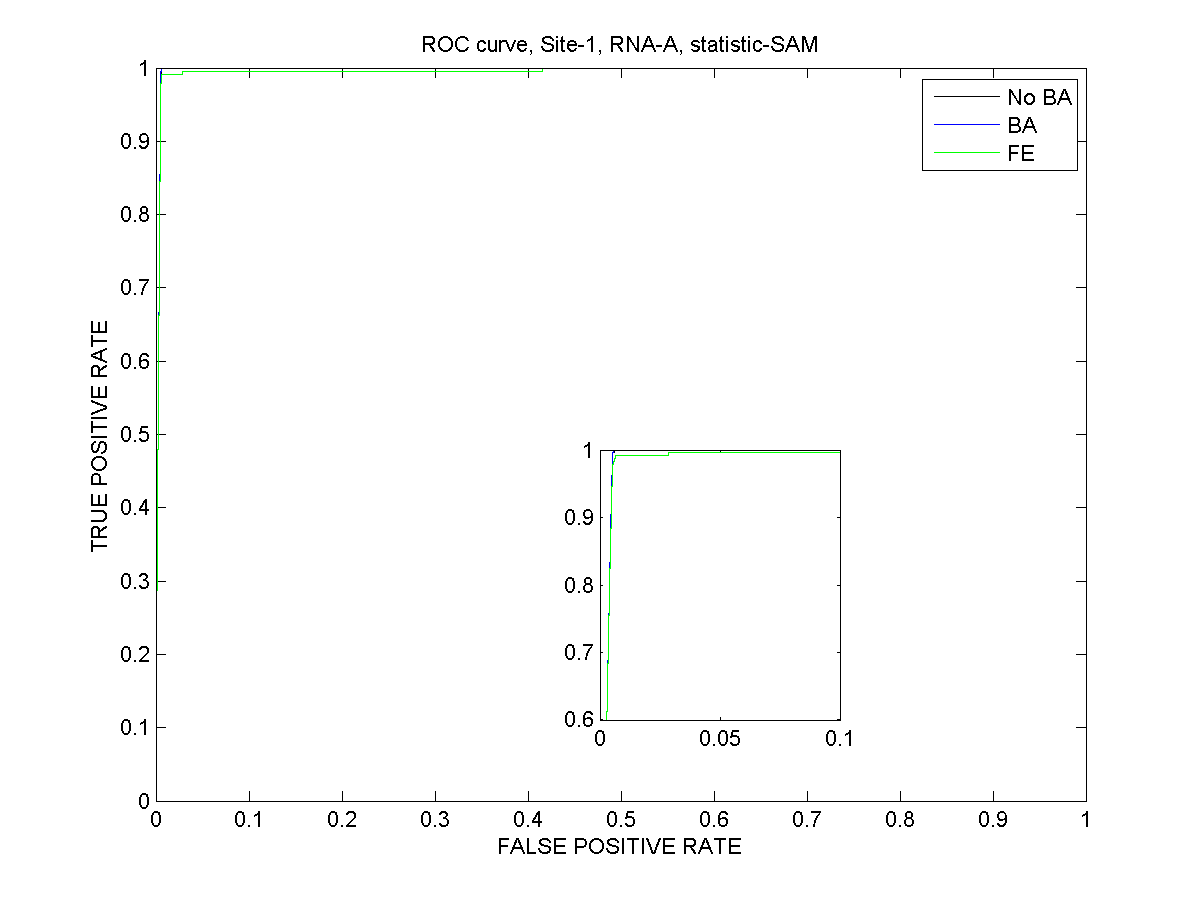

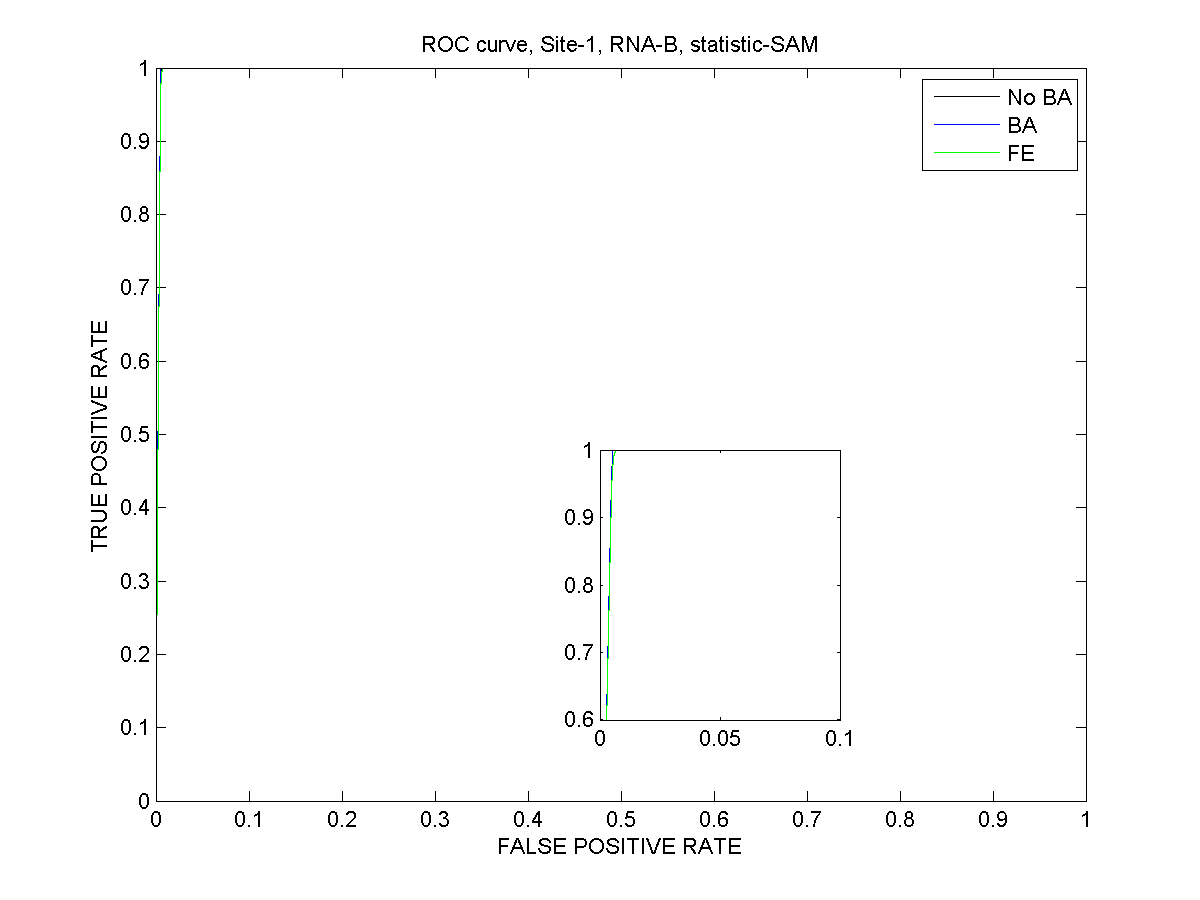

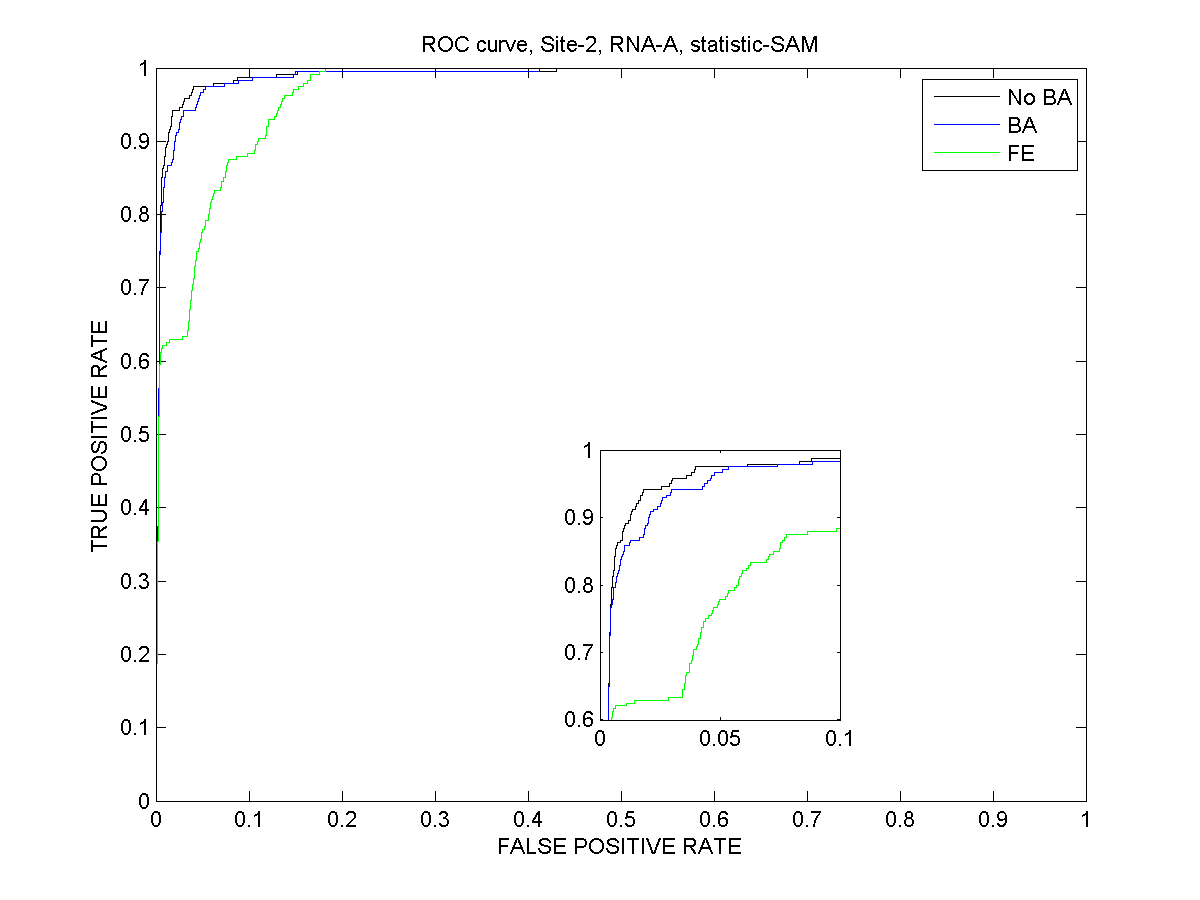

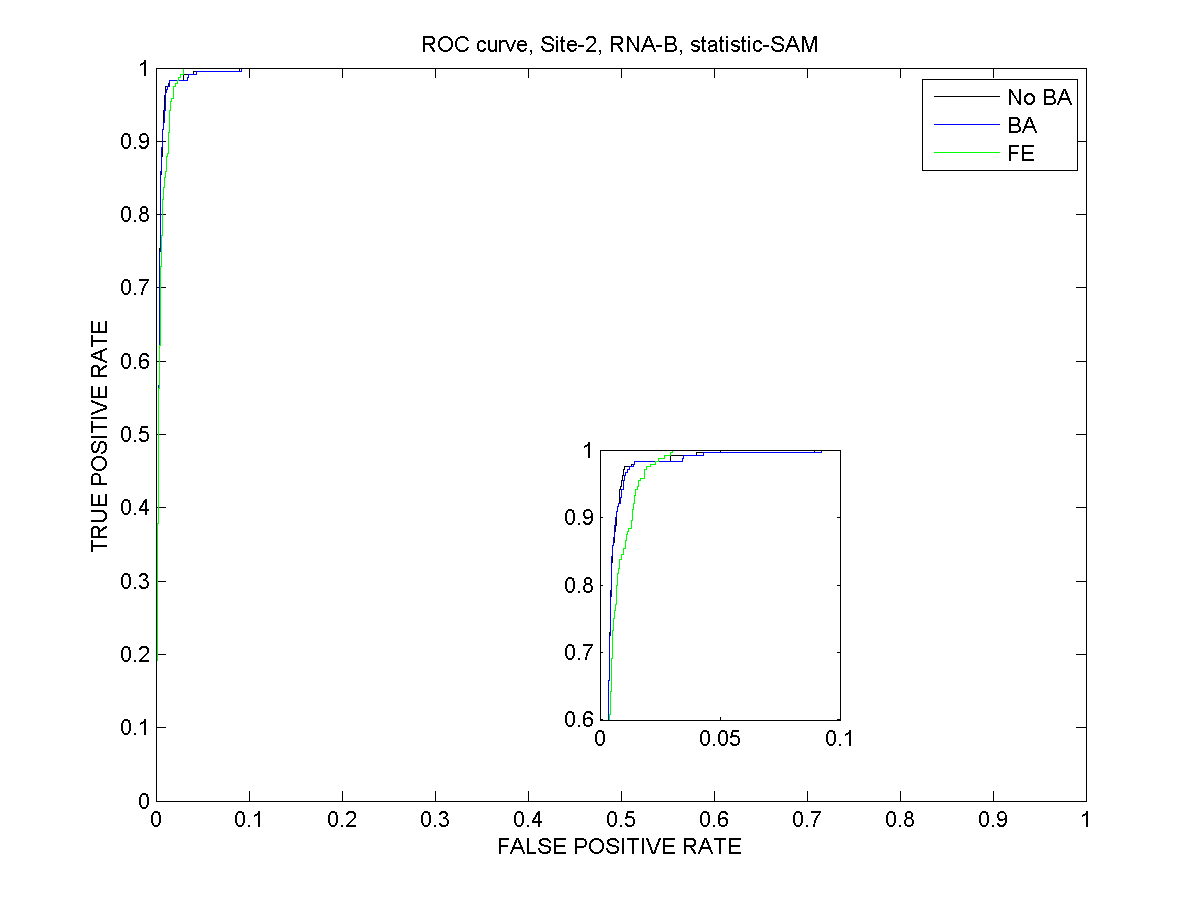

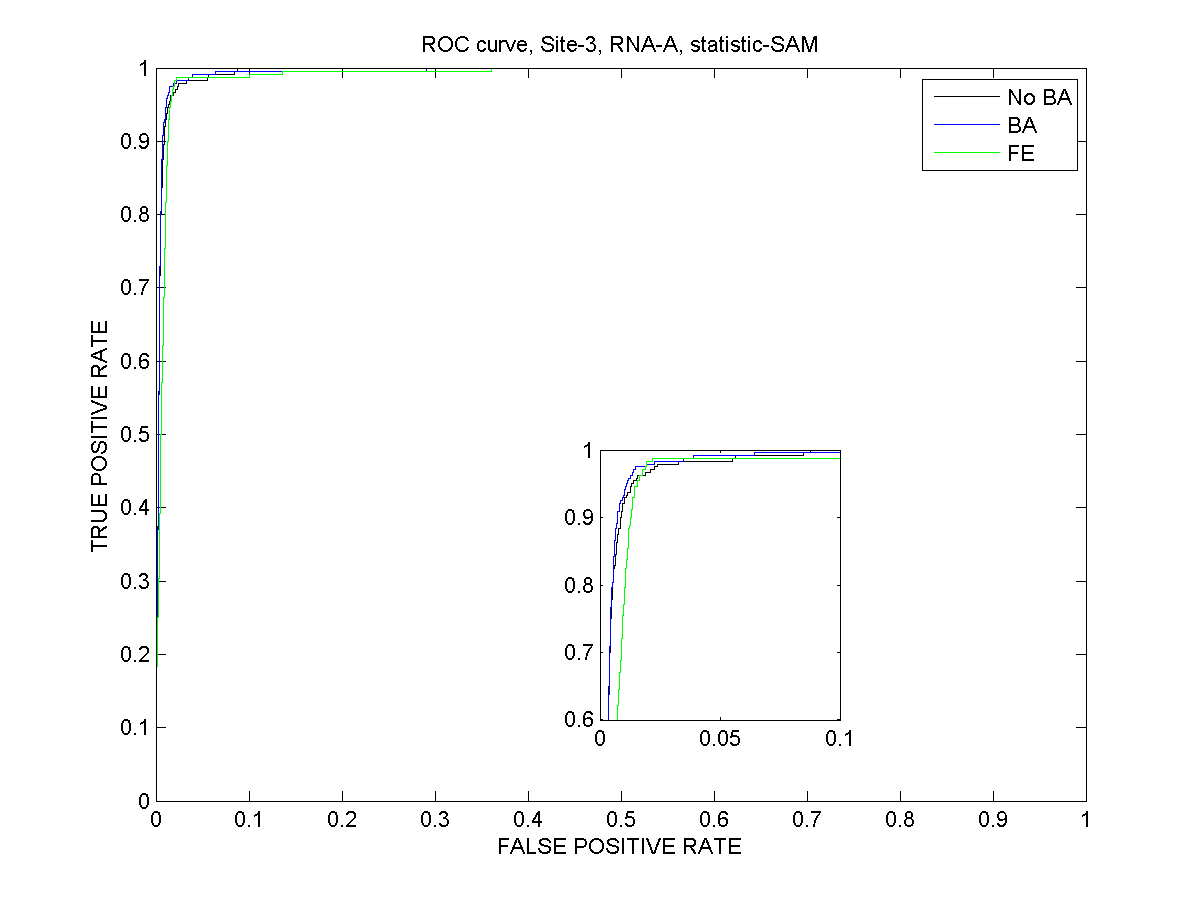

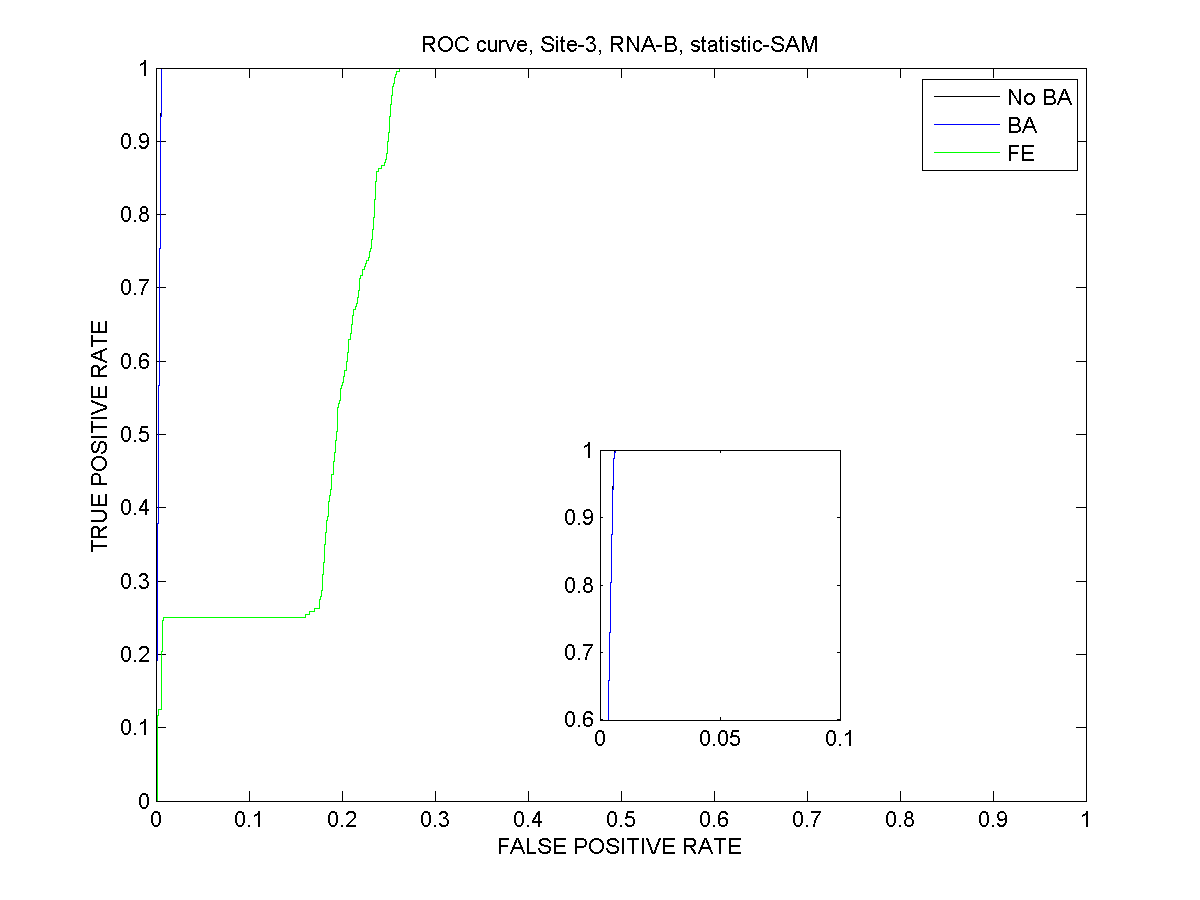

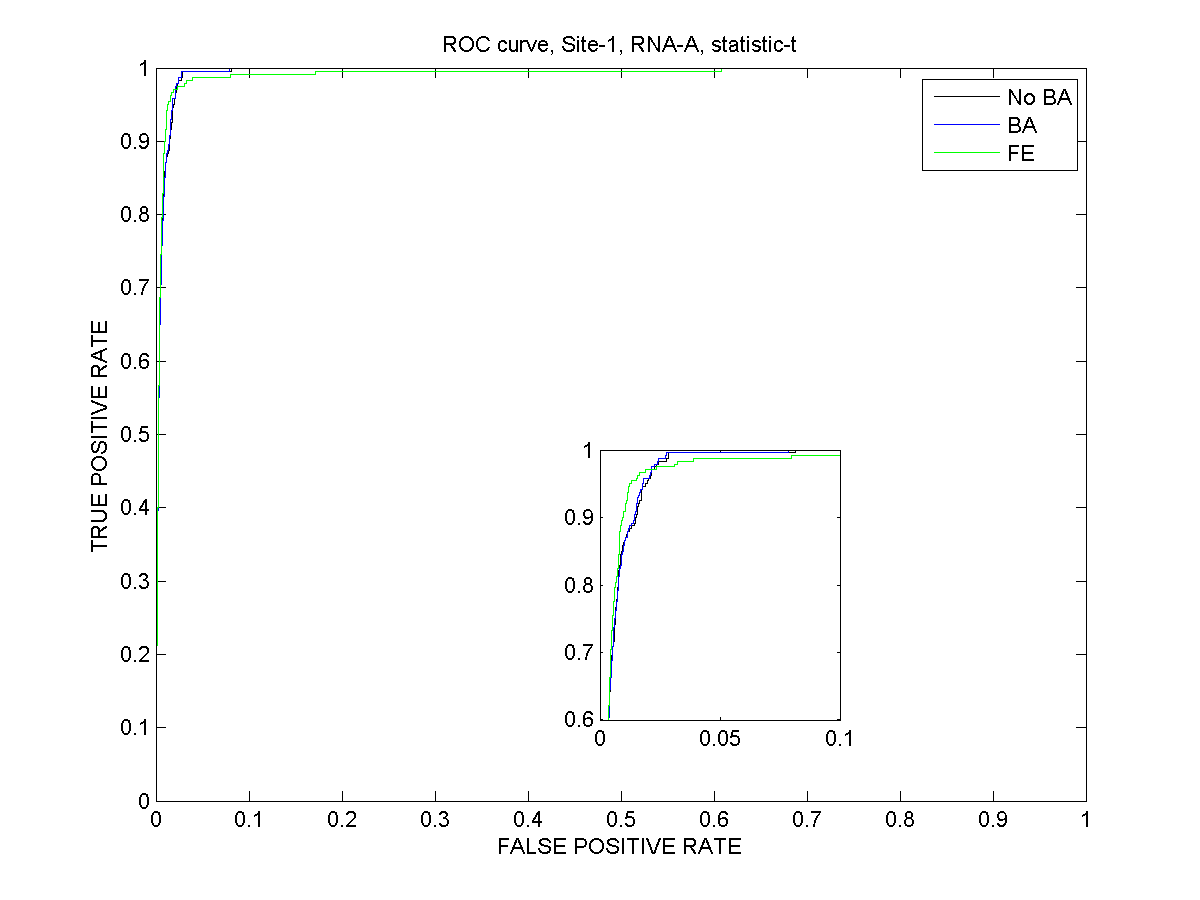

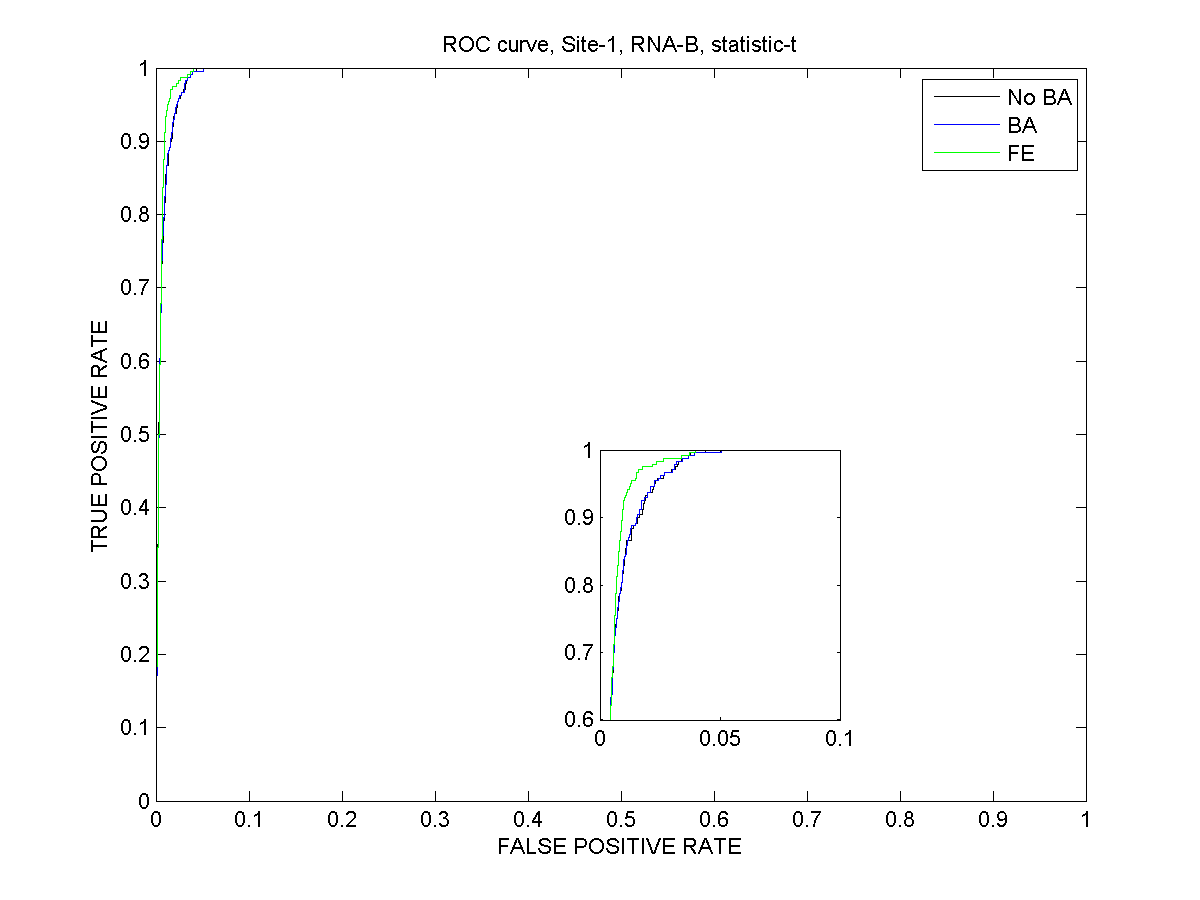

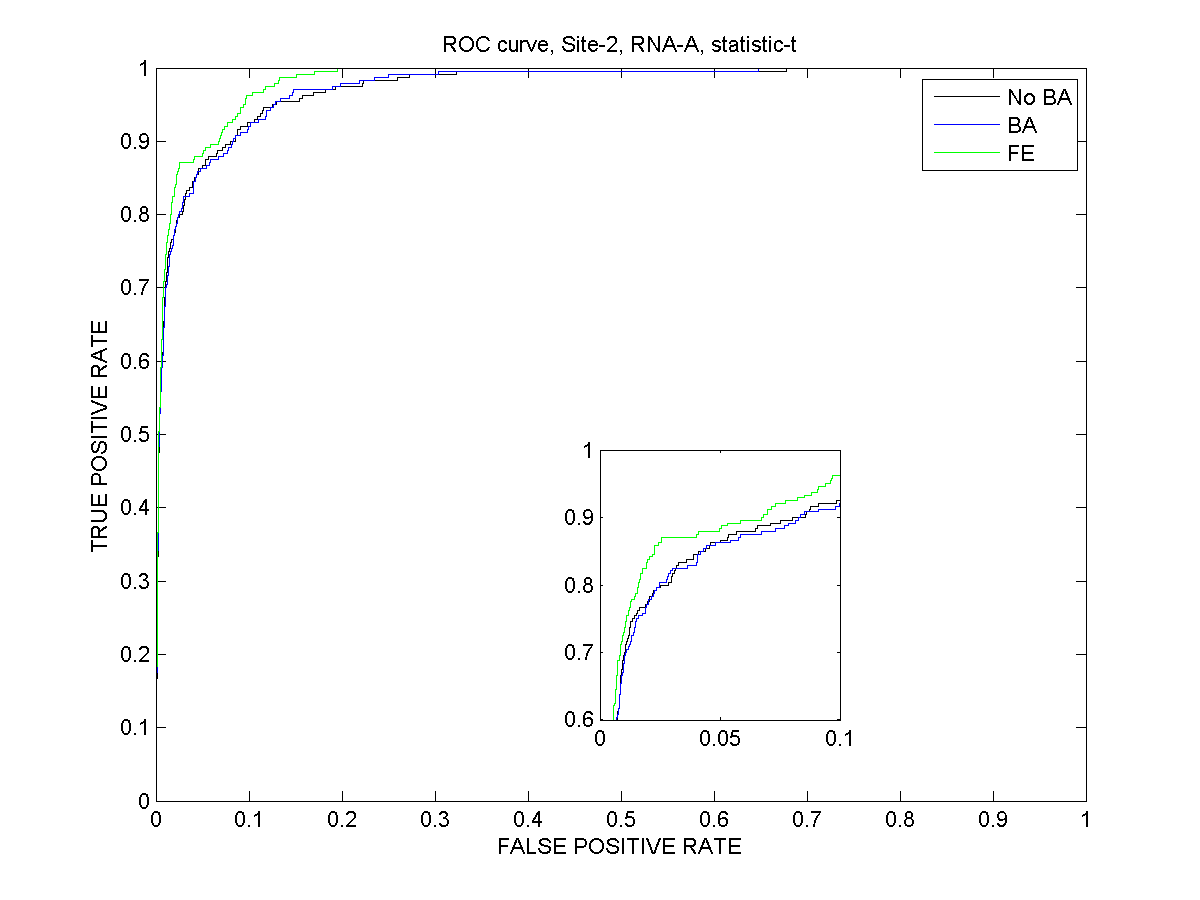

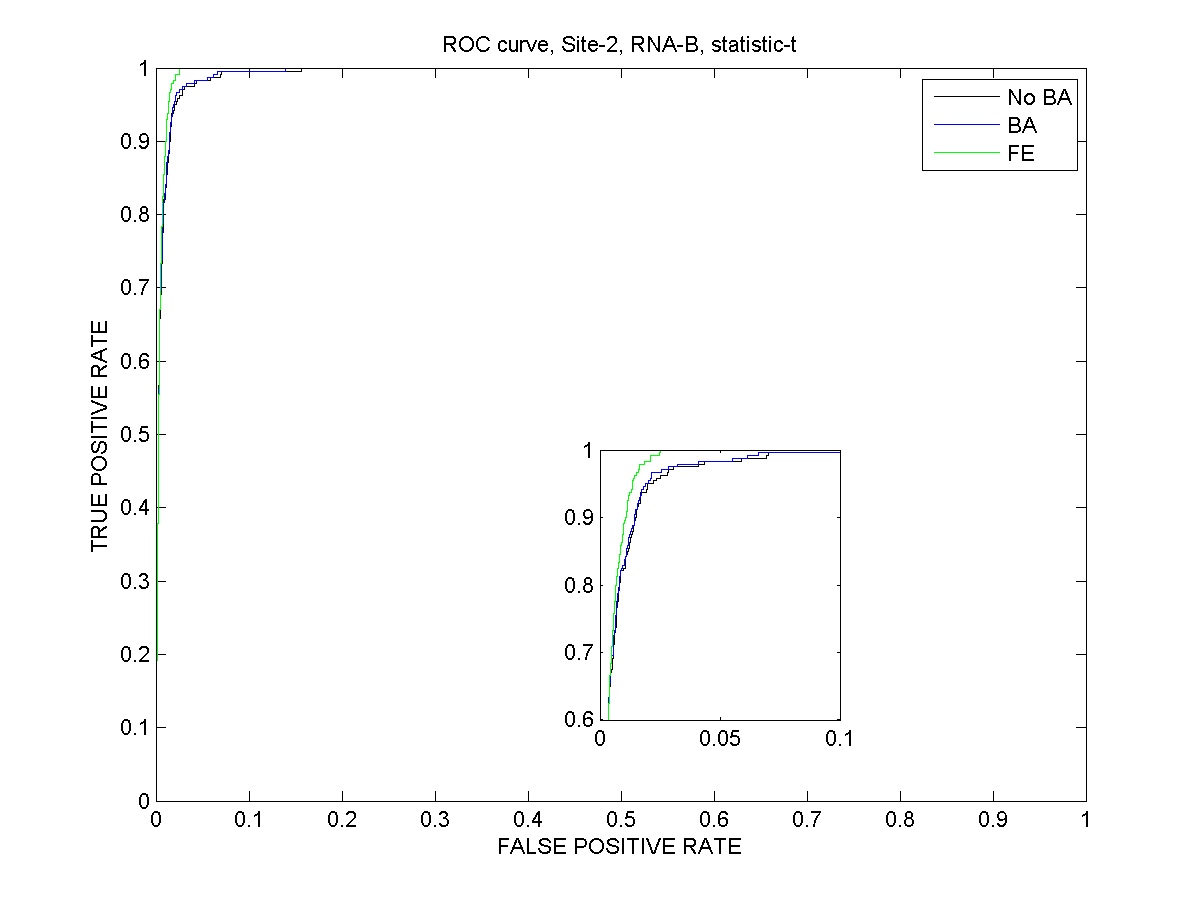

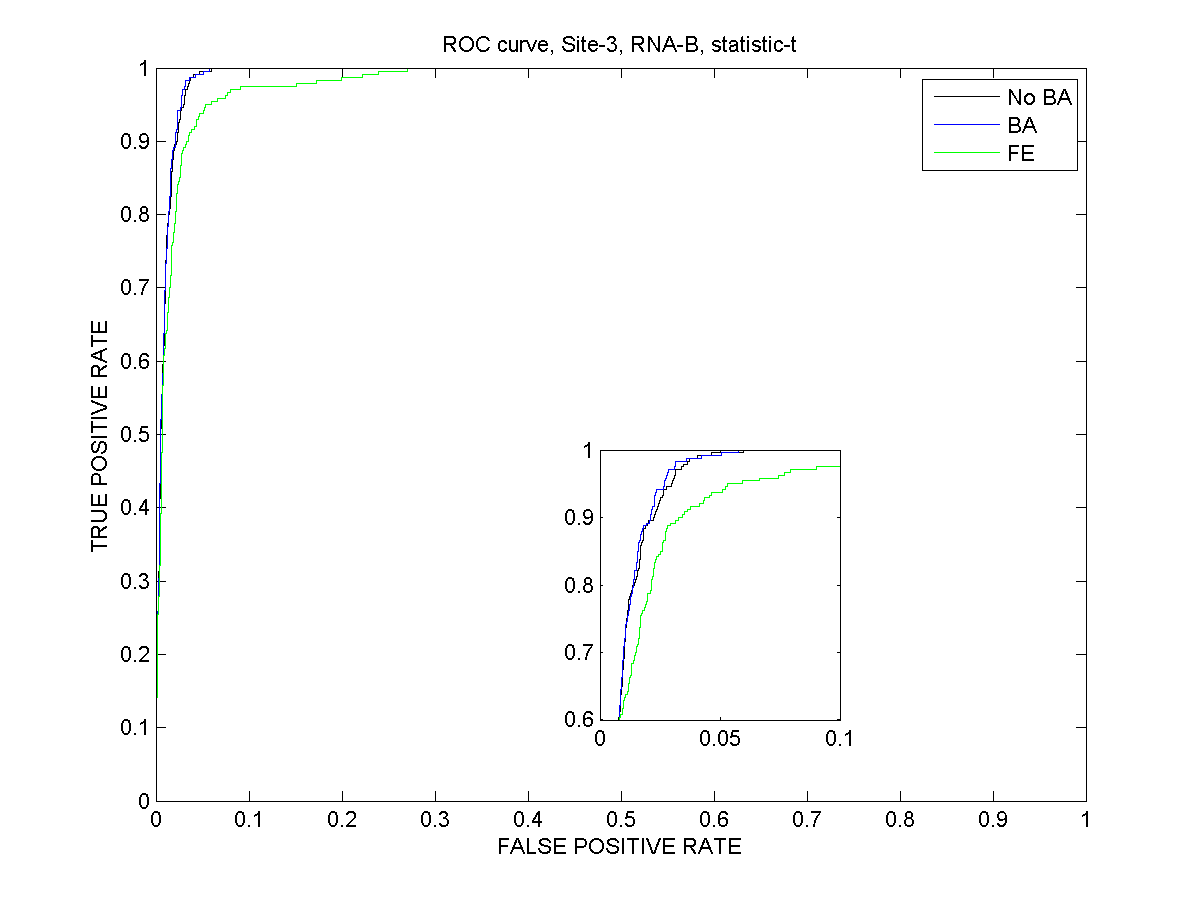

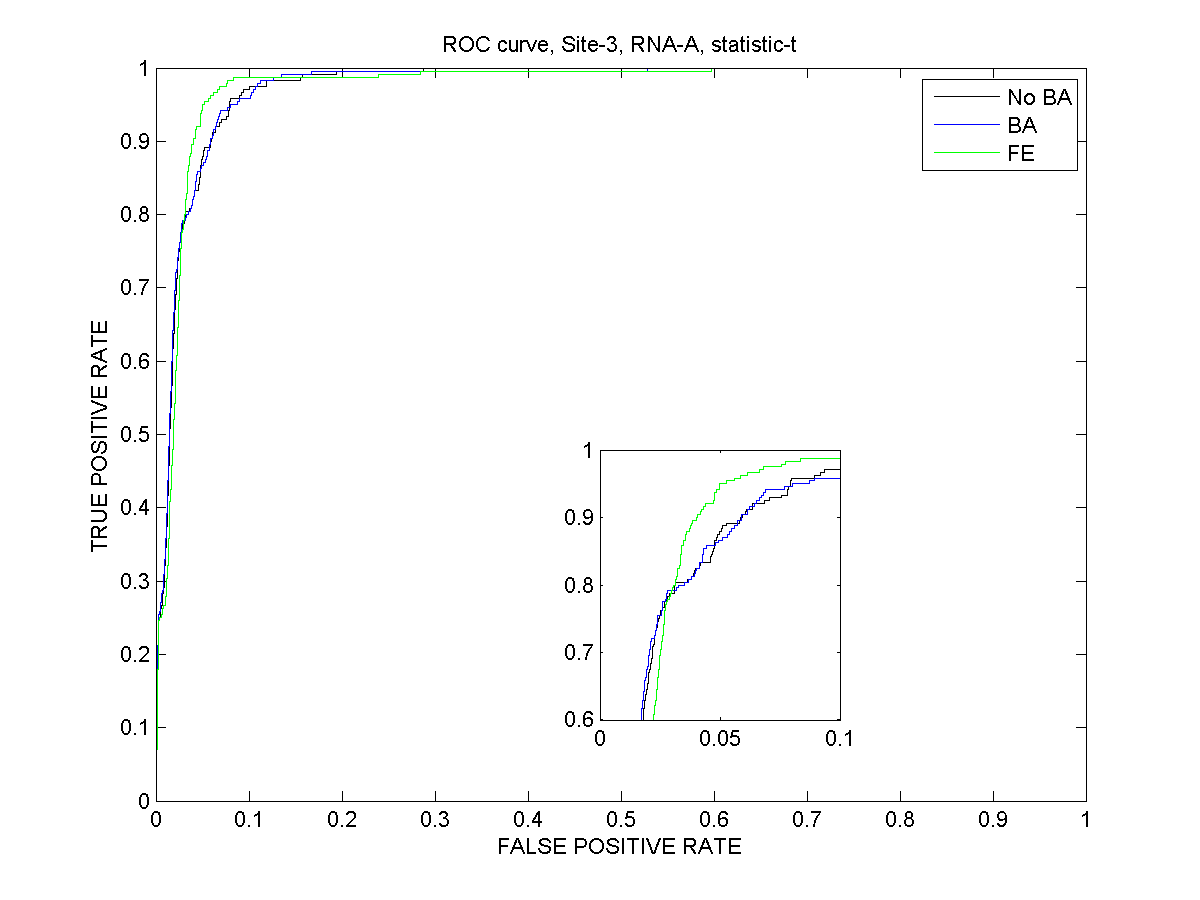

Supplement: Additional file 2 — ROC curves for the mean, t-statistic, and SAM statistic. The plots summarize the sensitivity and specificity for detecting differentially expressed genes using three different test statistics applied to three versions of the data. [file 1471-2105-8-371-S2.doc]
