# Supplementary material for: Extended analysis of benchmark datasets for Agilent two-color microarrays
Source: BMC Bioinformatics. 2007 Oct 3;8:371. doi: 10.1186/1471-2105-8-371 (PMC2174956; doi:10.1186/1471-2105-8-371)

Supplement 3. Test statistic values plotted against average spot intensity. Green points are ERCs with non-zero log-ratios. The vertical axis represents a test statistic (mean, t-statistic, or SAM statistic). The horizontal axis is a measure of spot intensity. Green points are ERCs with non-zero log-ratios. Ideally, the green points in the plot are separated, vertically, from the black points.


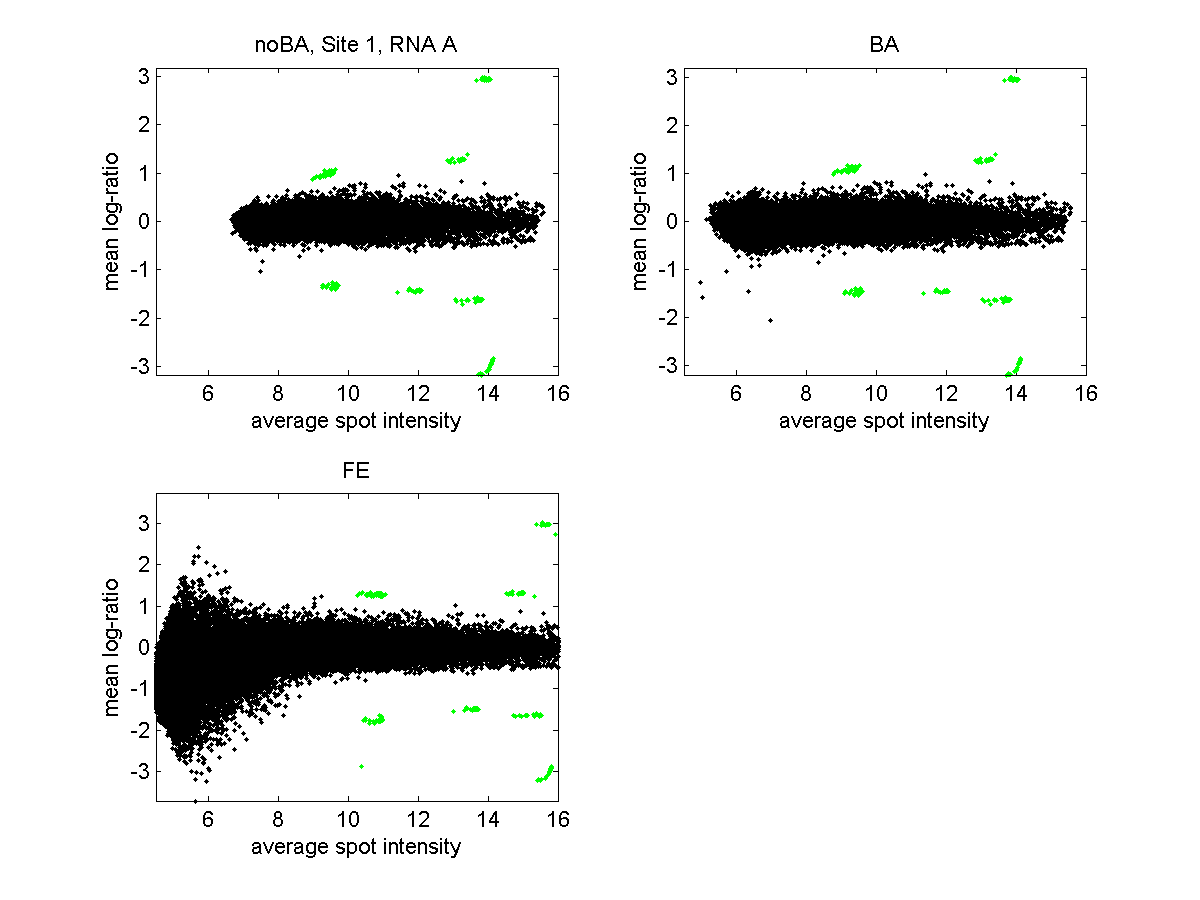

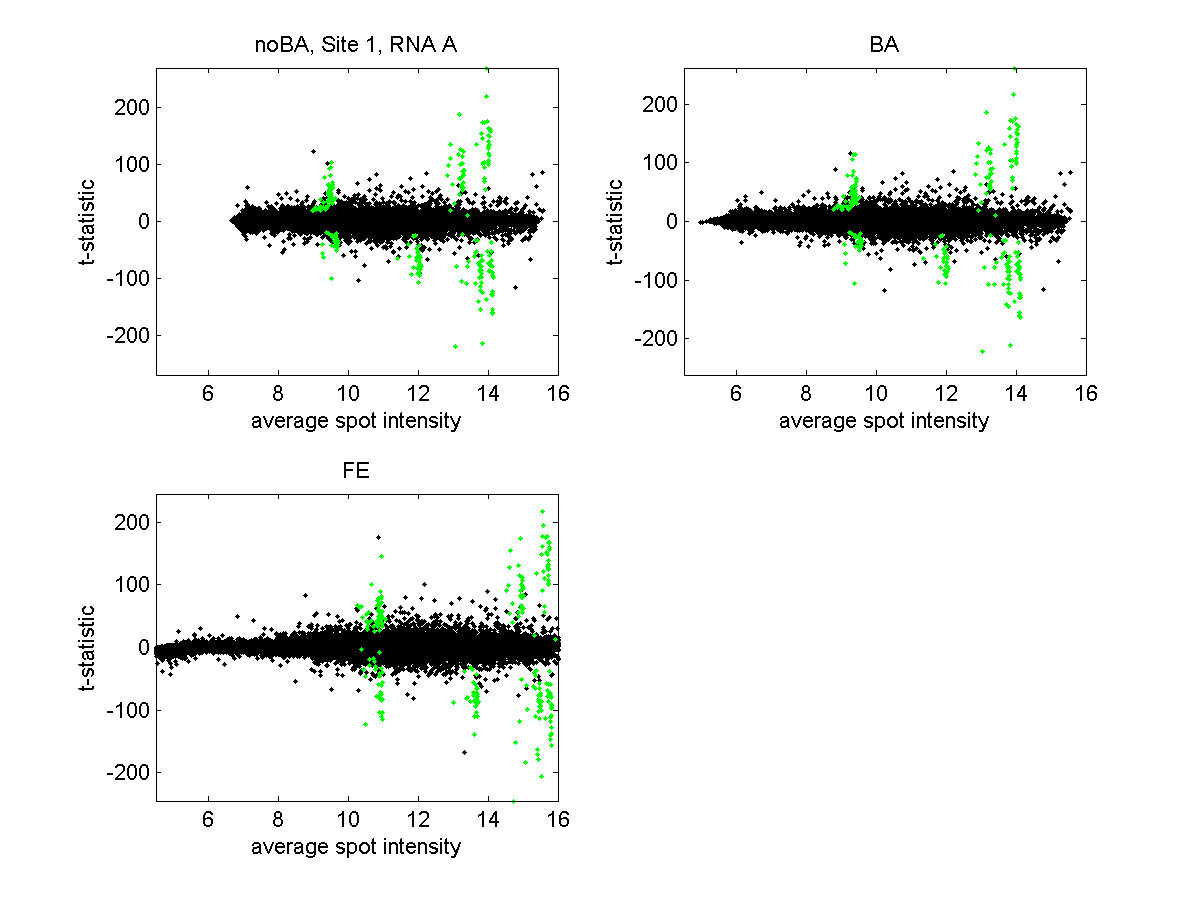


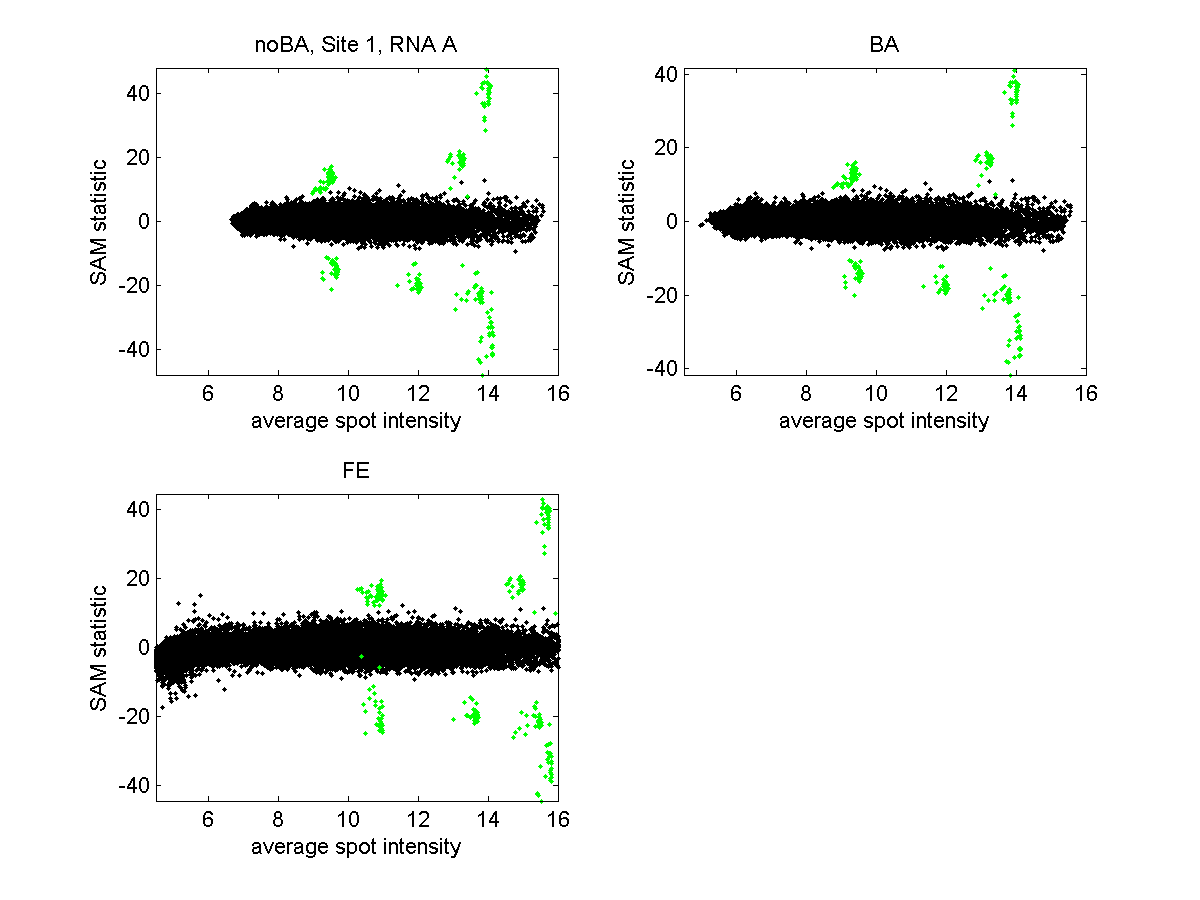


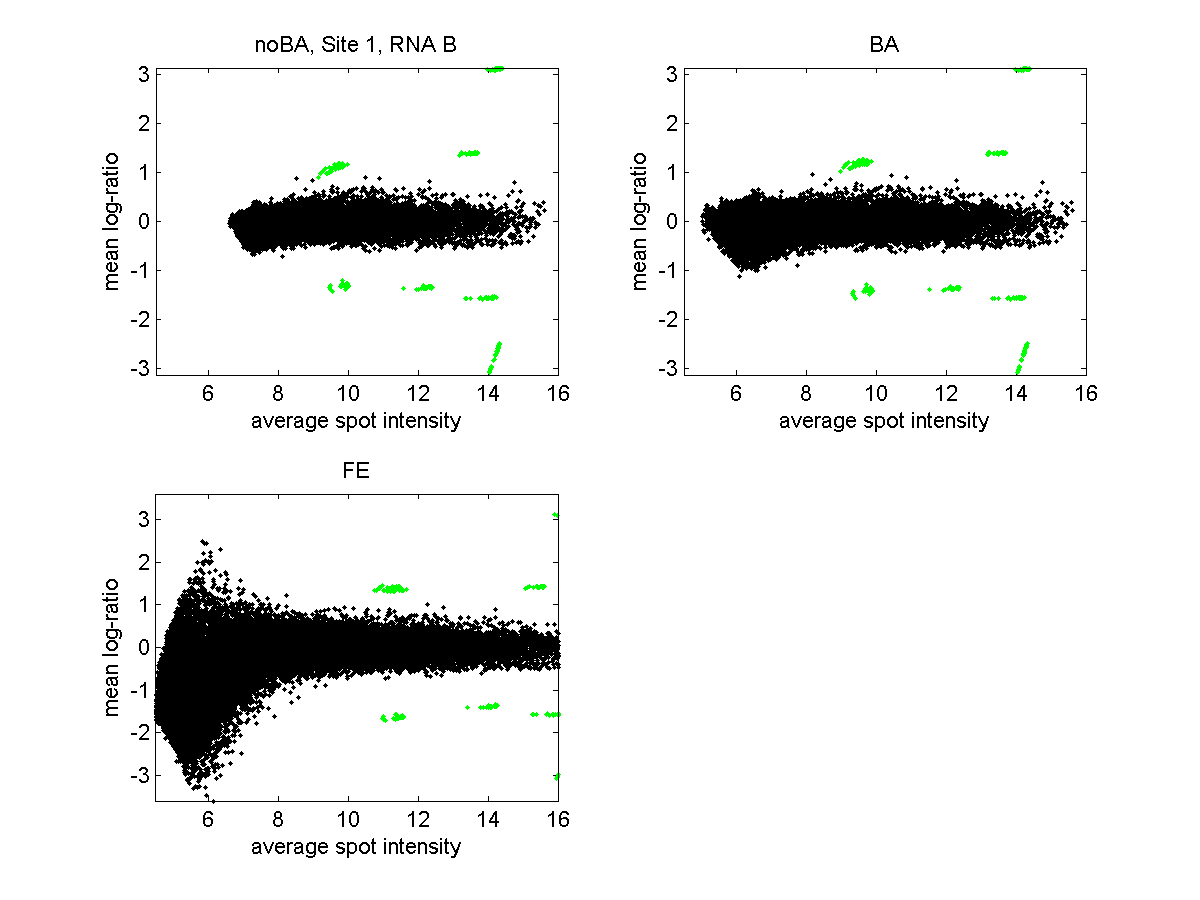

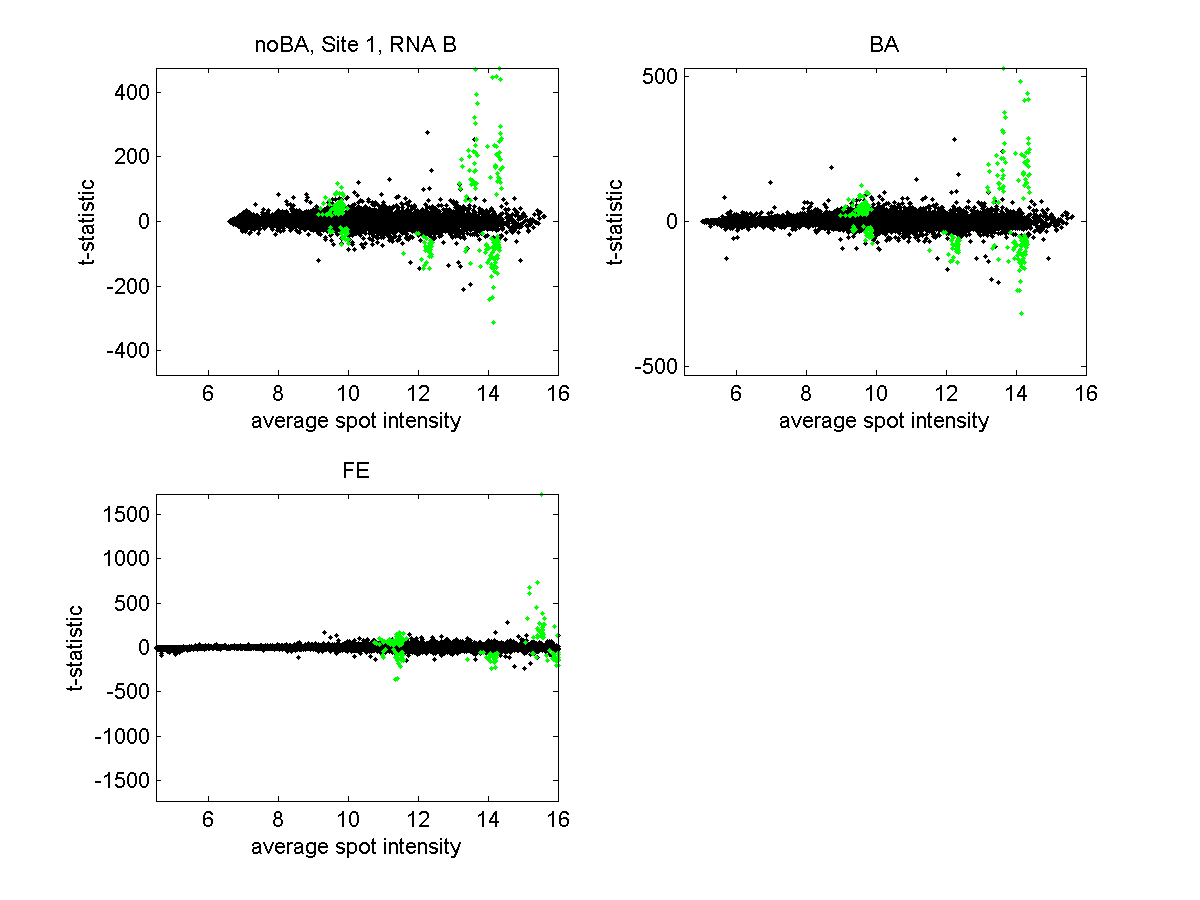


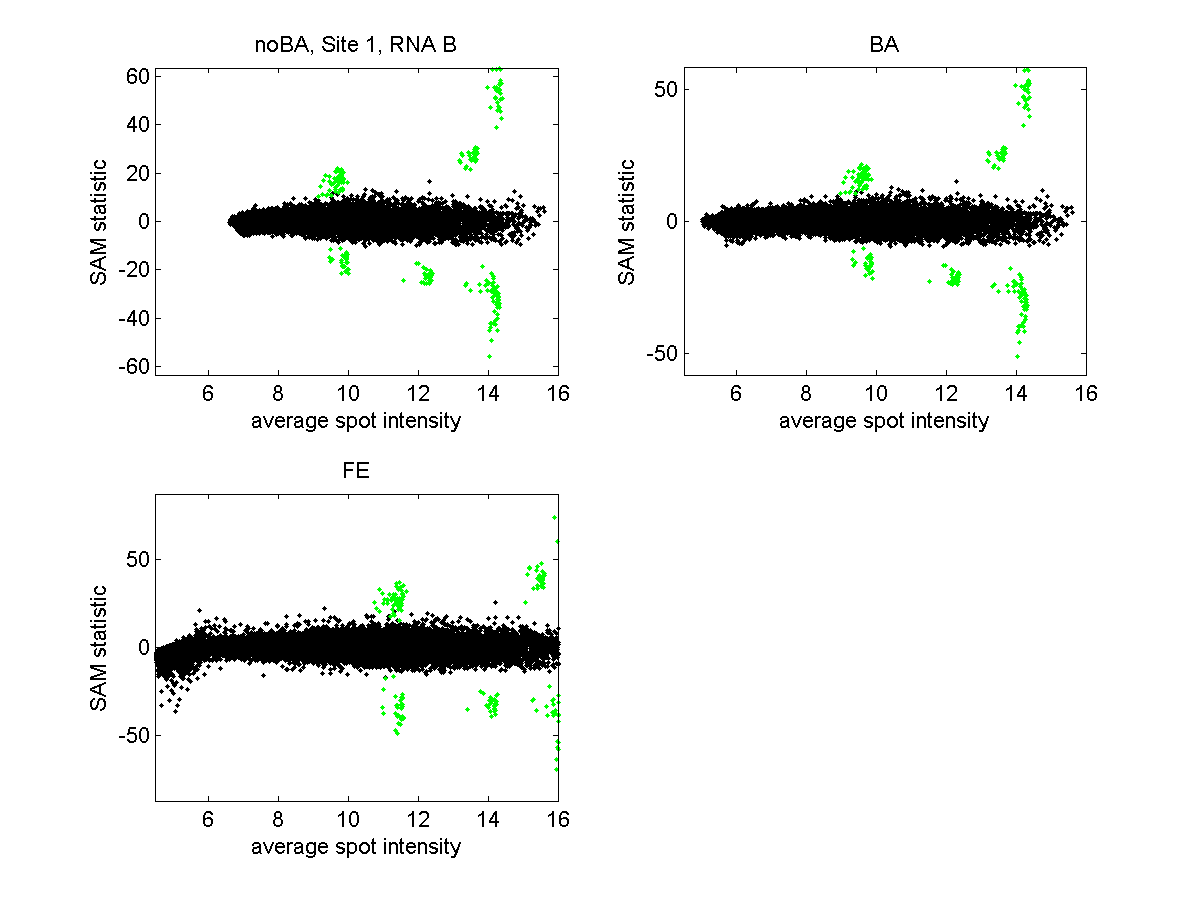


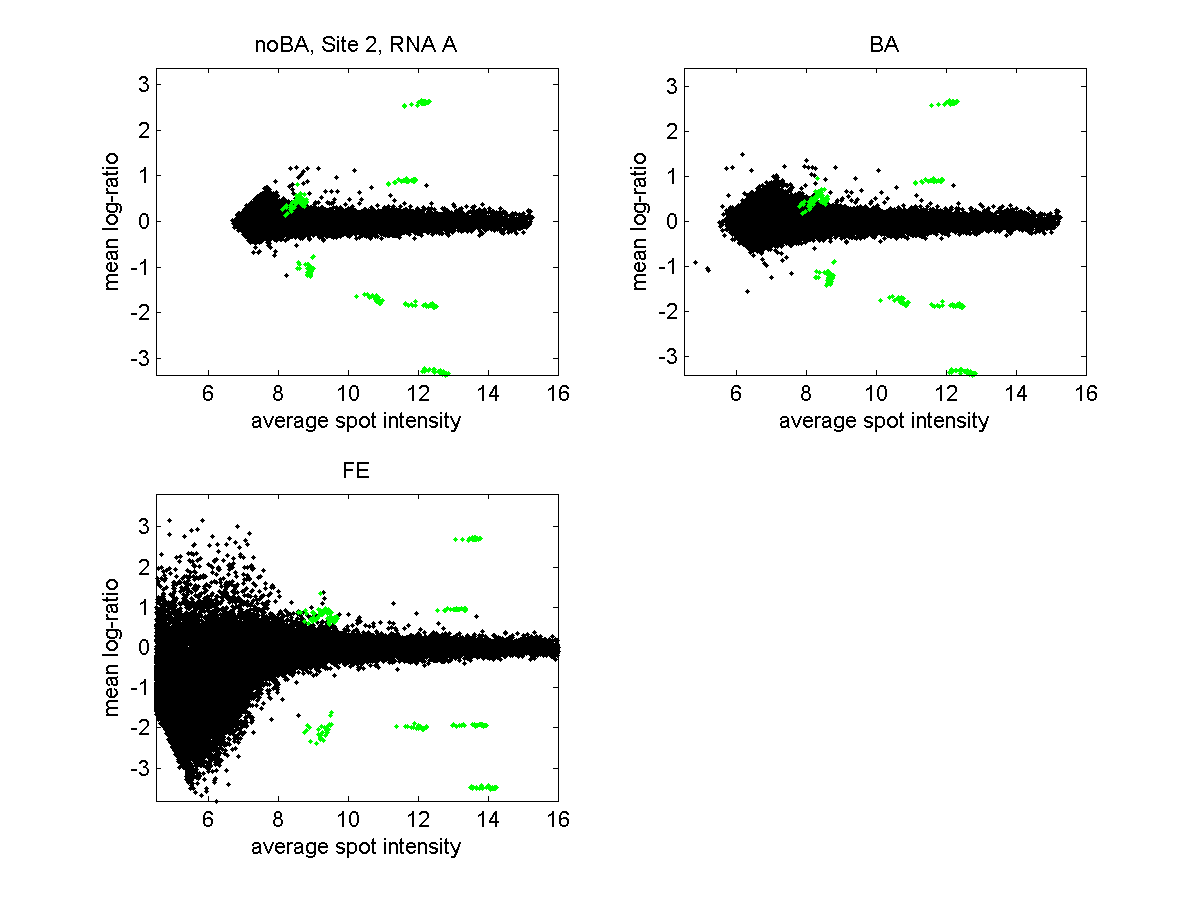

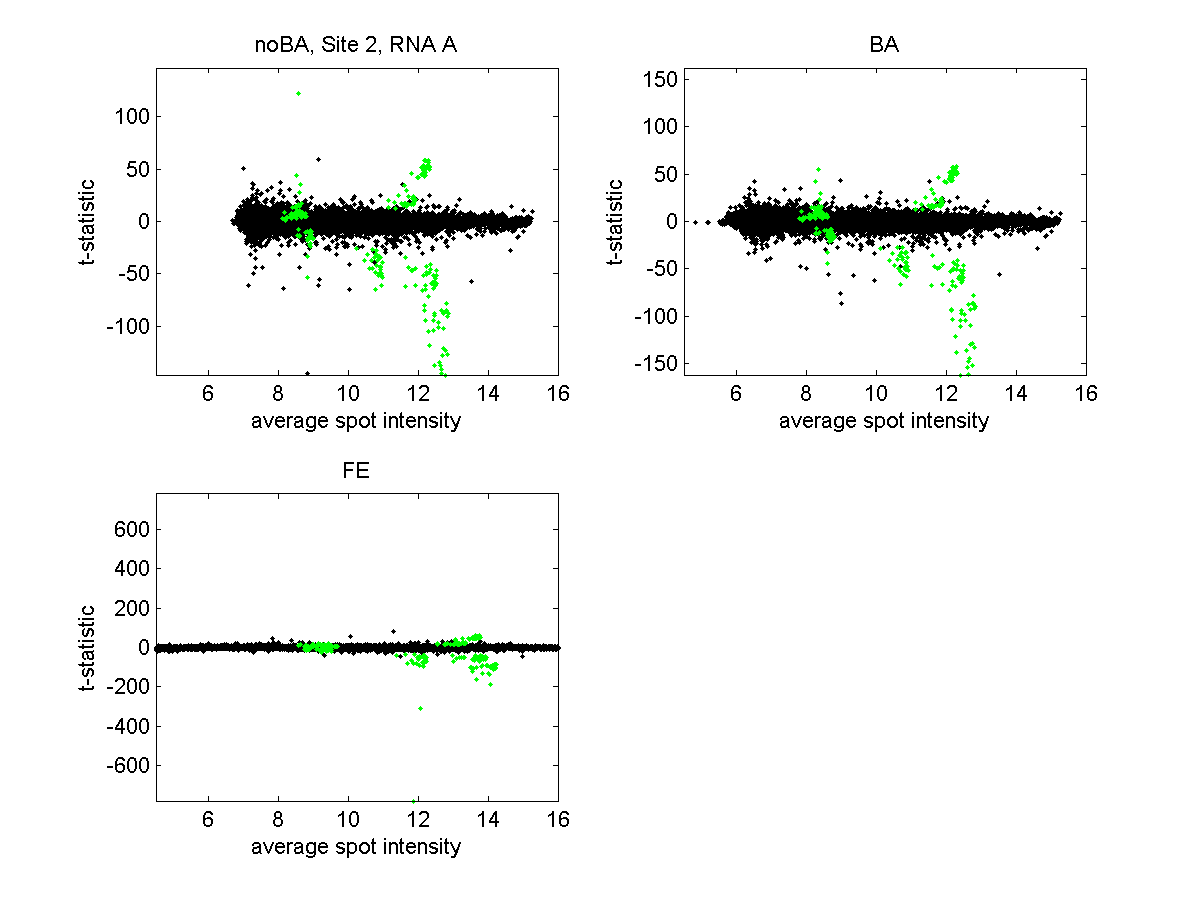

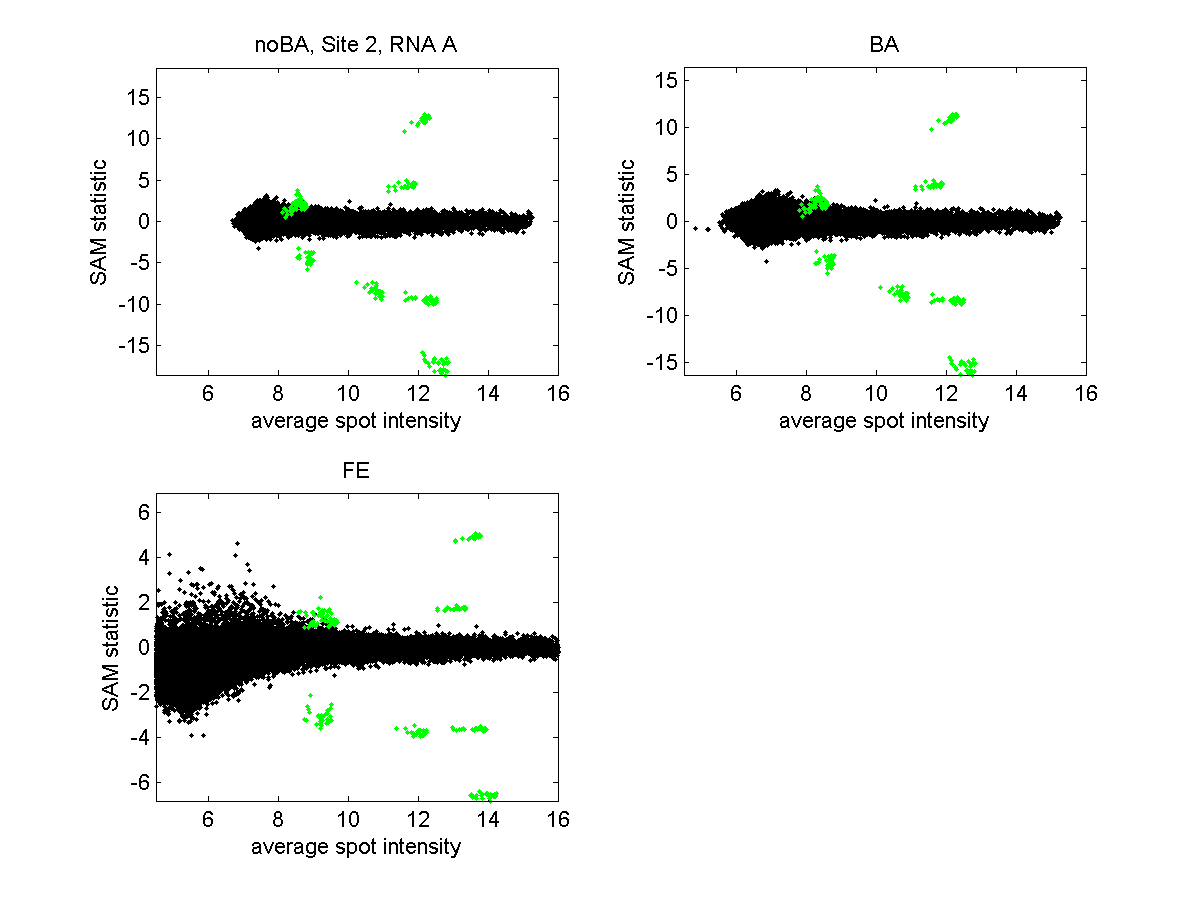


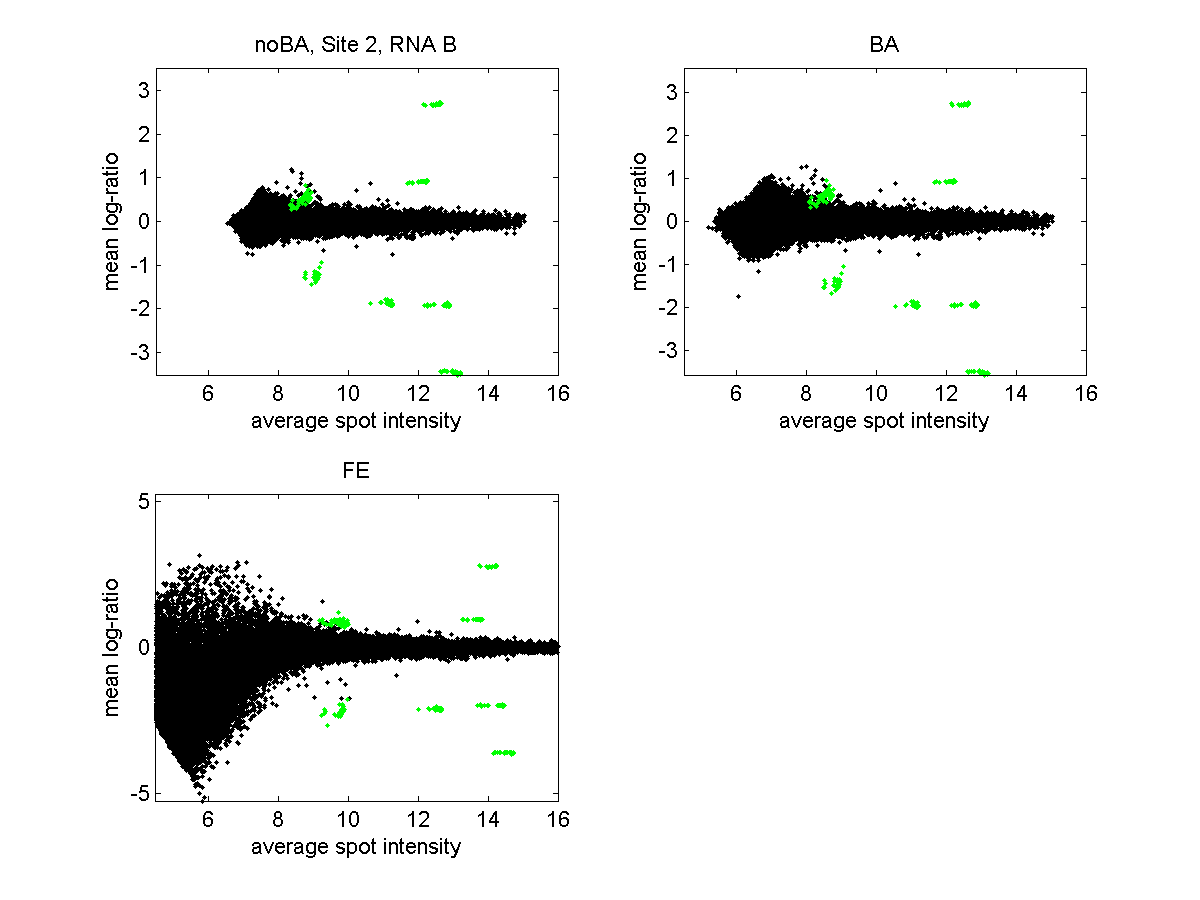

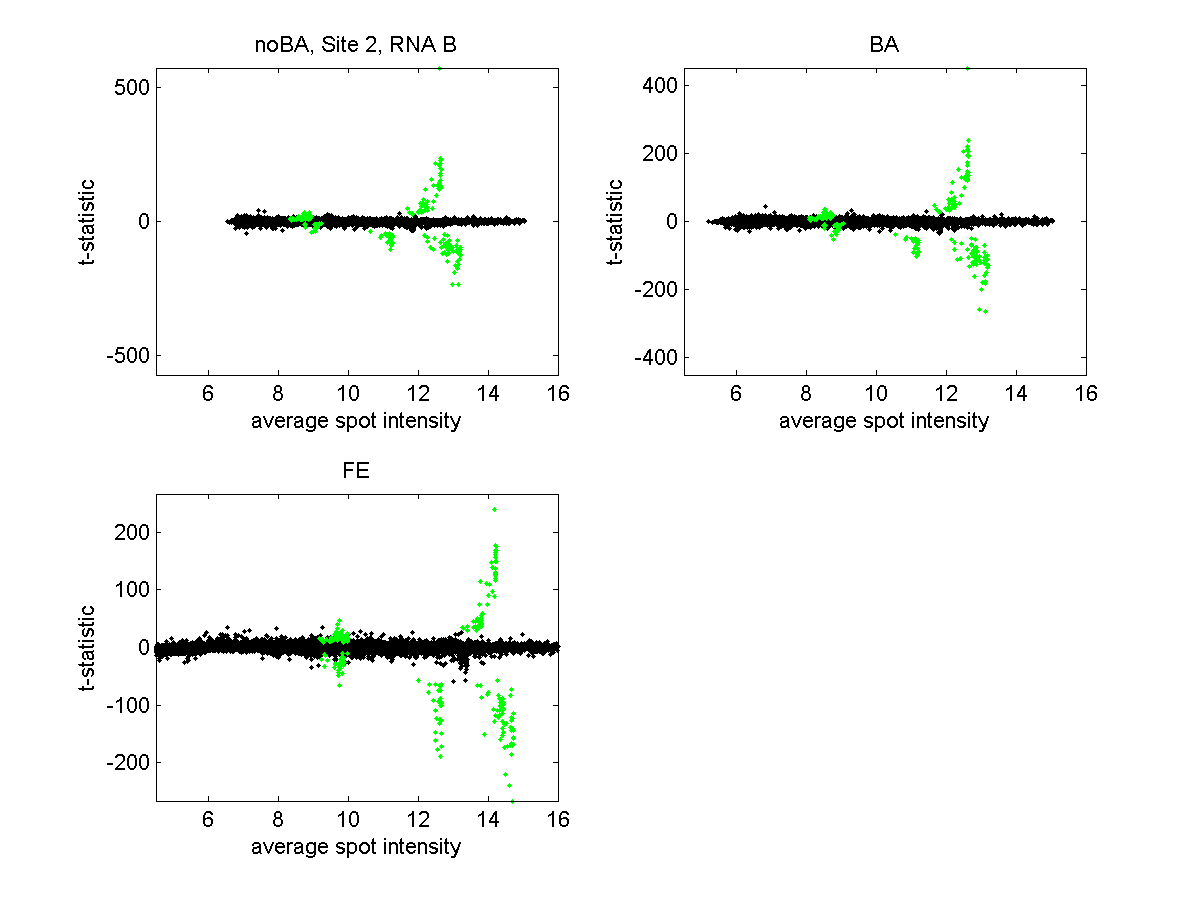


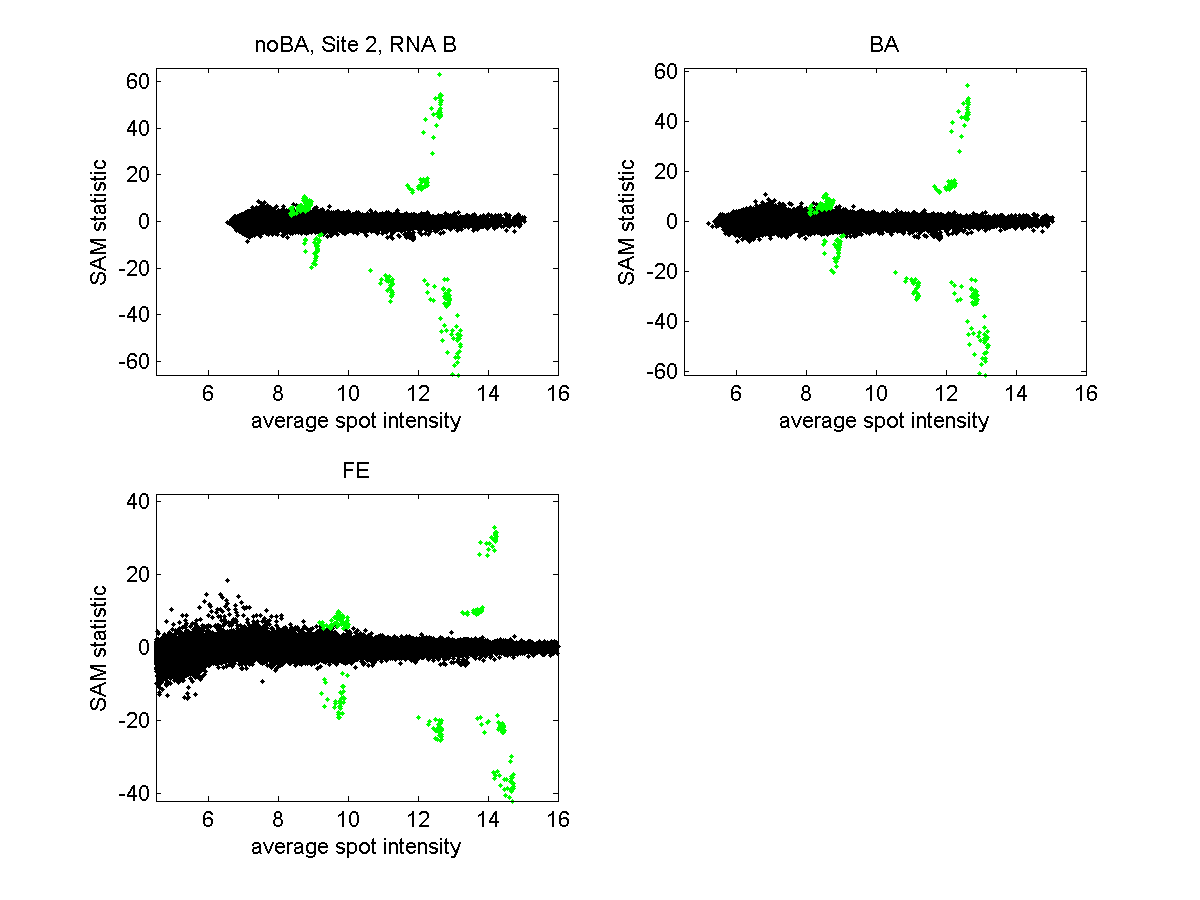


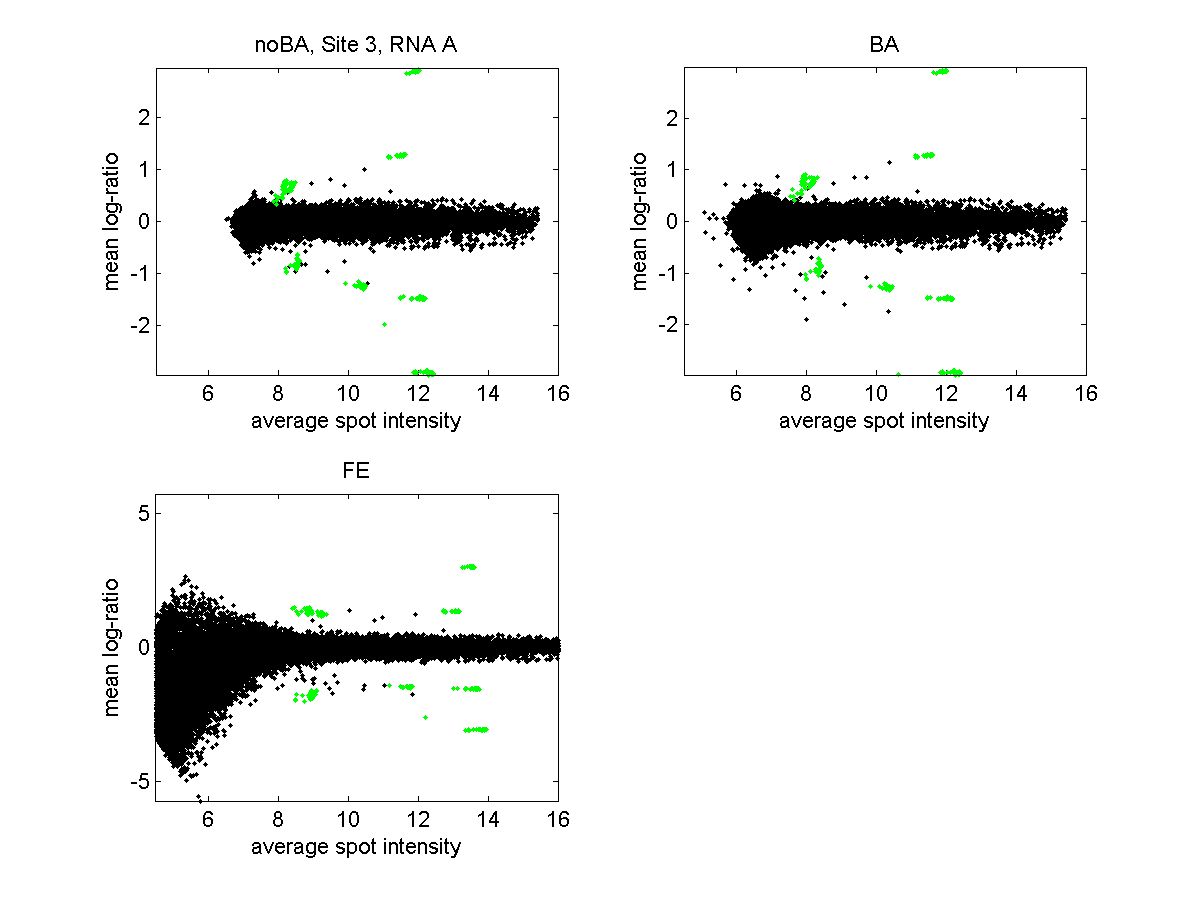

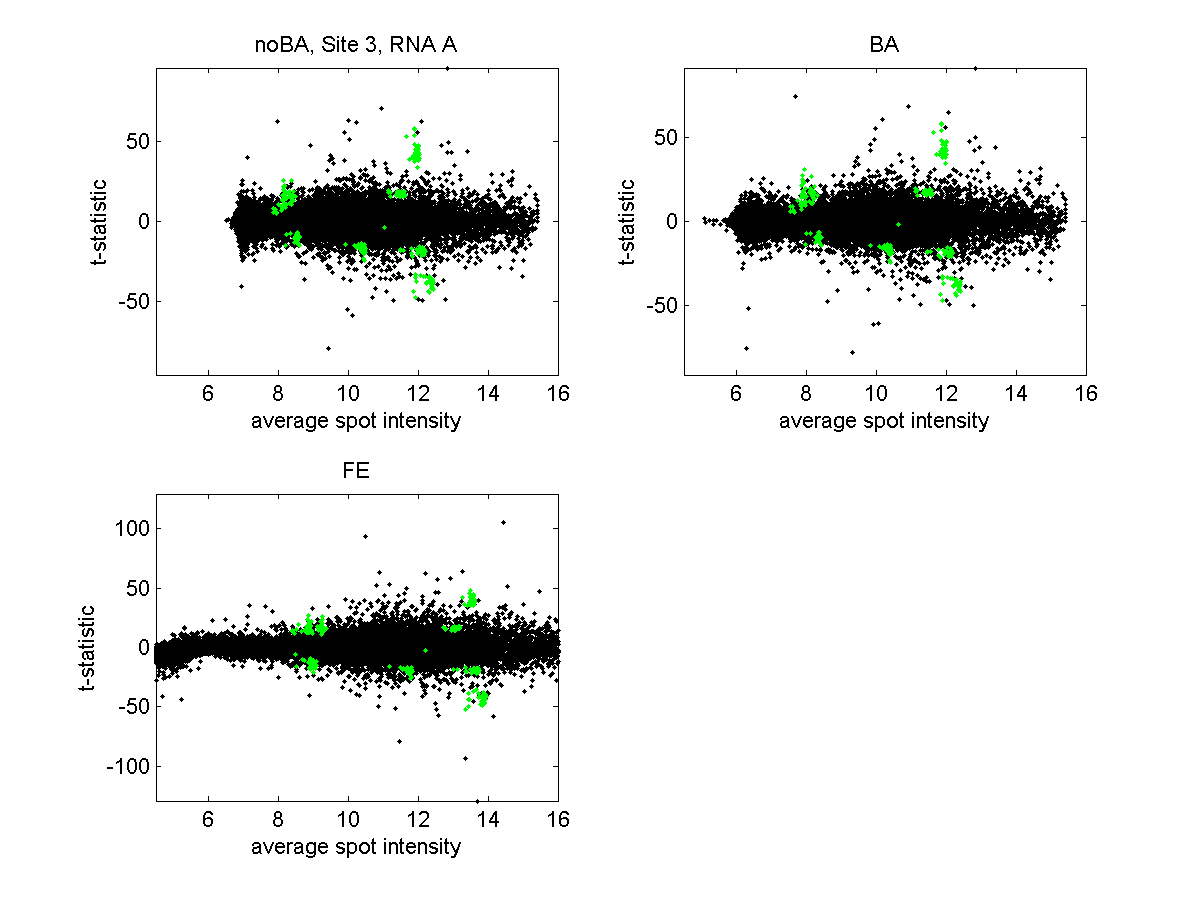

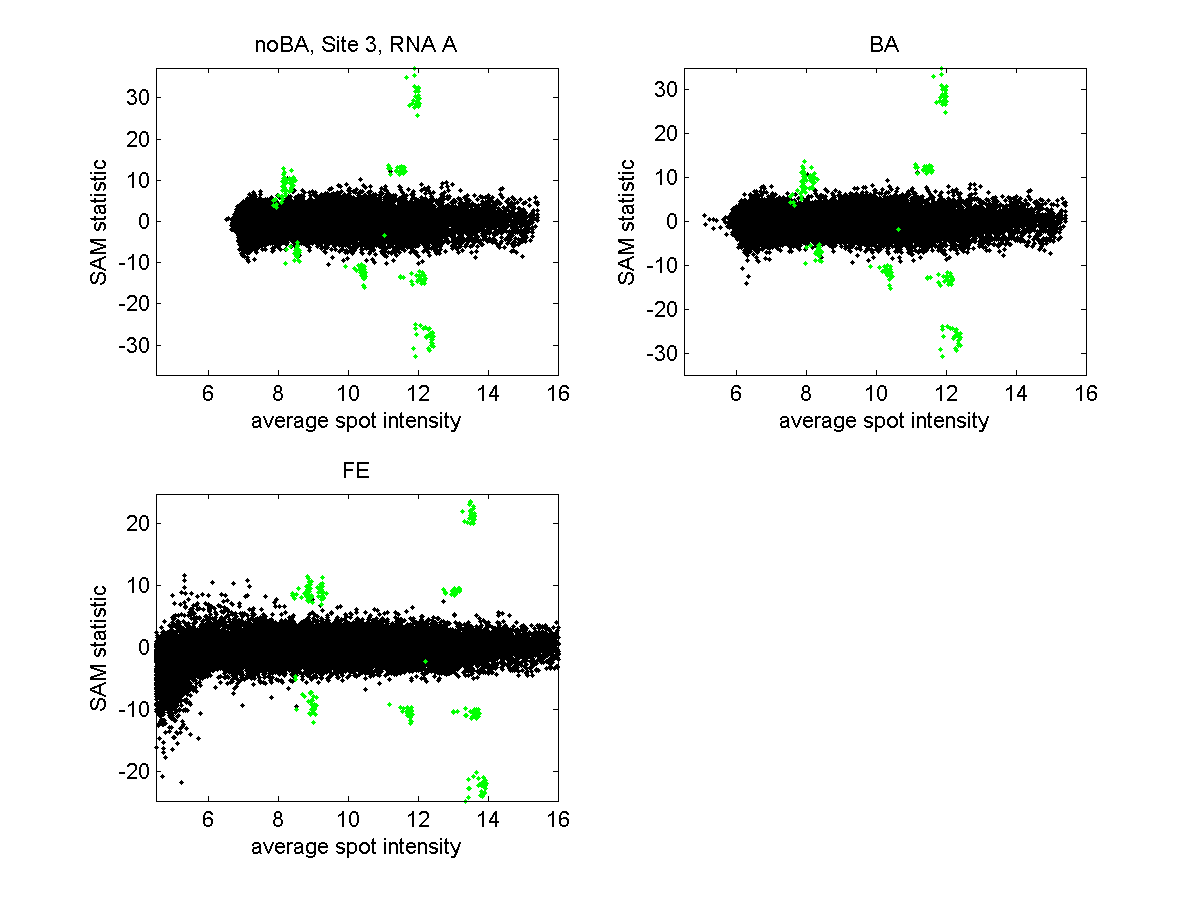


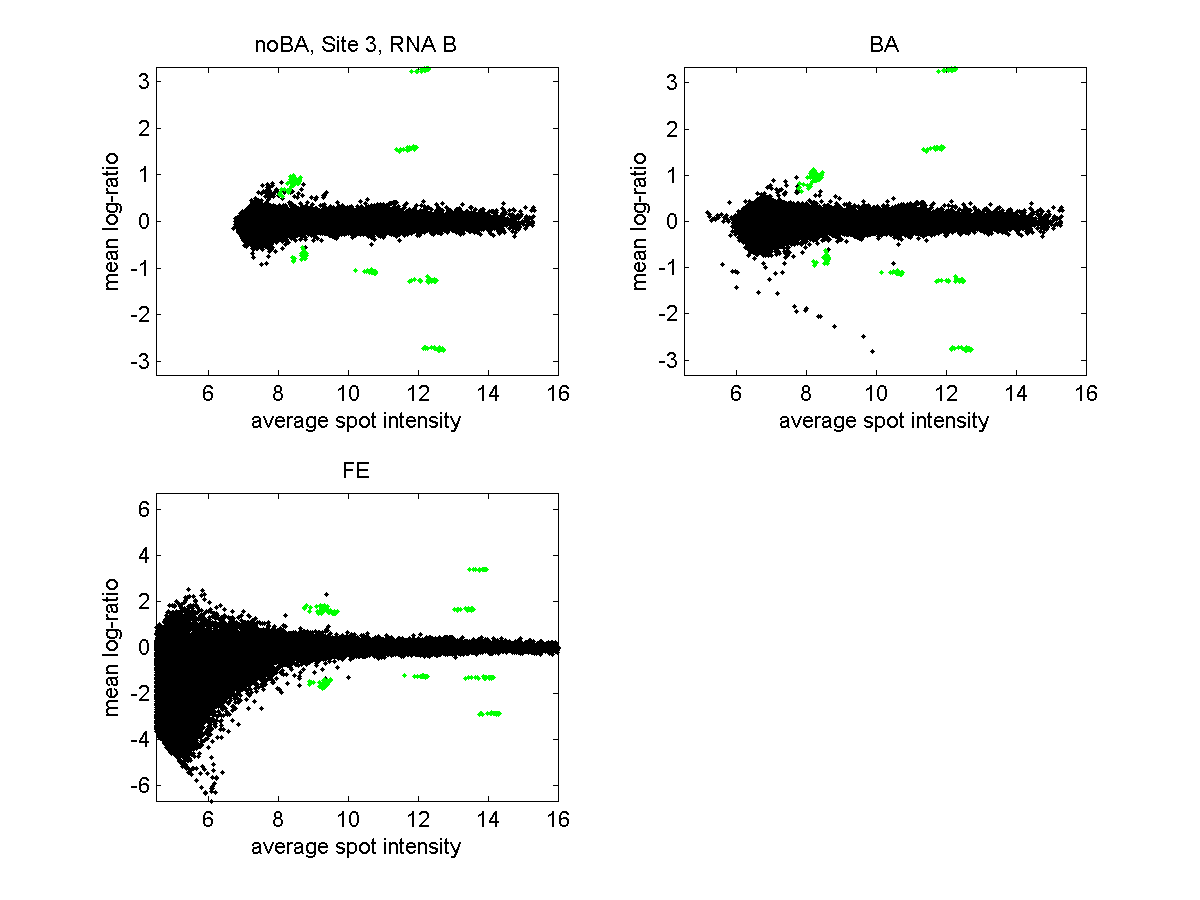

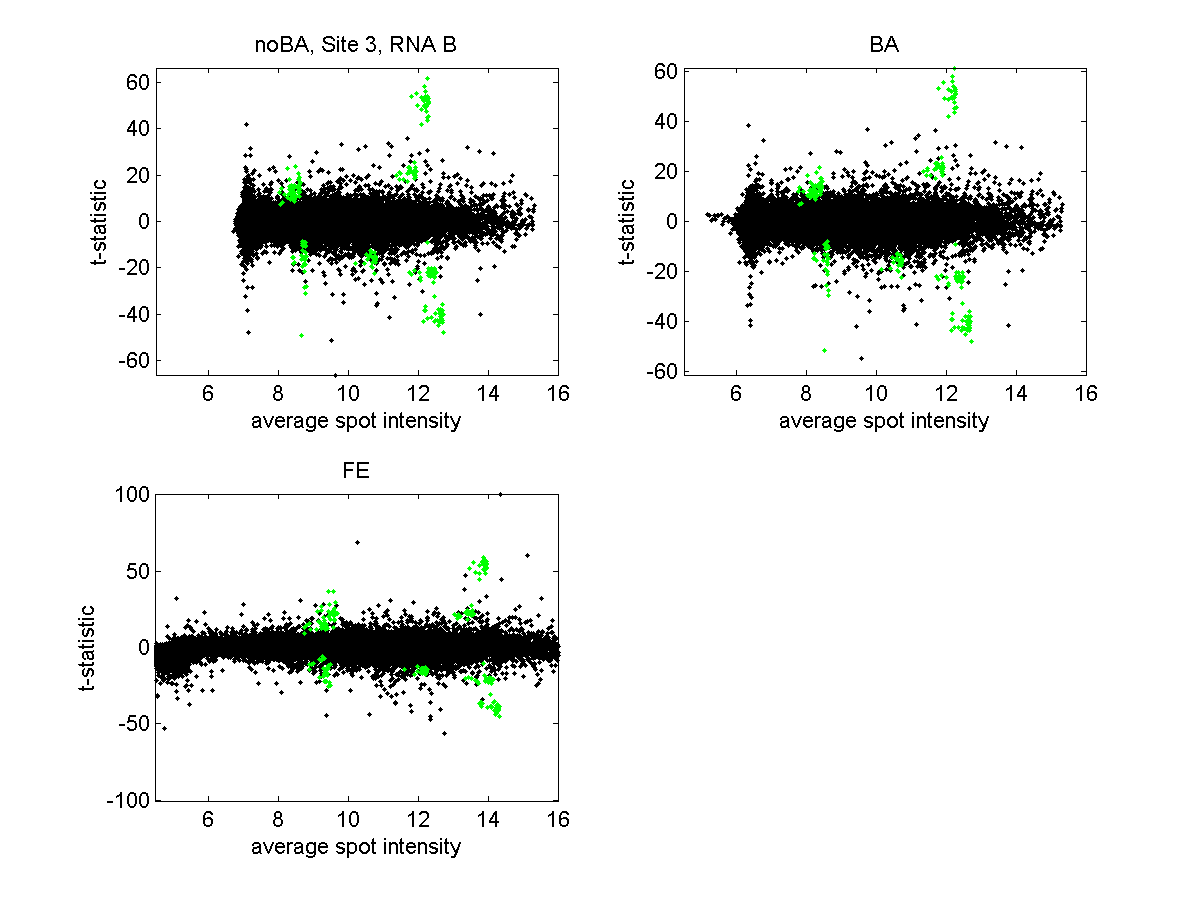

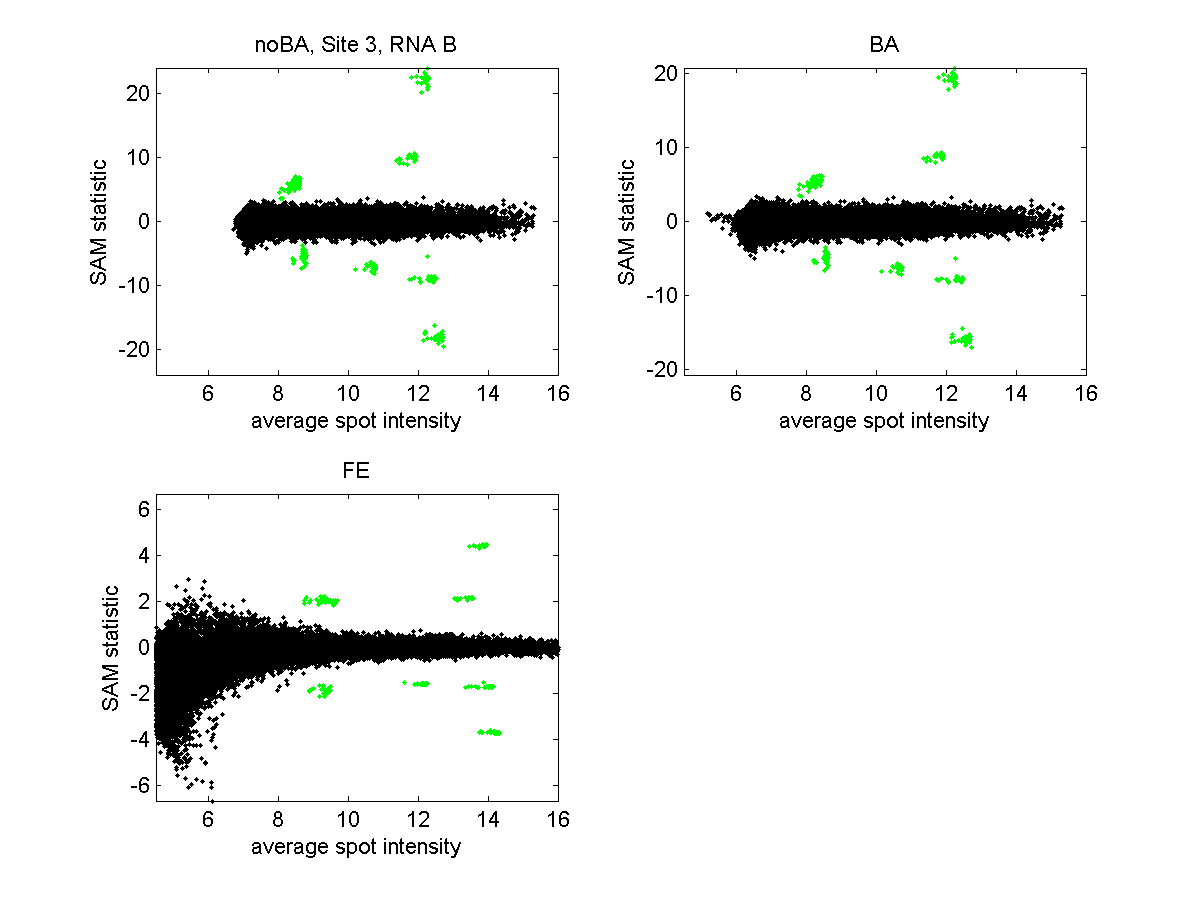

Supplement: Additional file 3 — Test statistic values plotted against average spot intensity. The plots show the behavior of test statistics as a function of signal intensity for three versions of the data. [file 1471-2105-8-371-S3.doc]
