# Supplementary material for: Extended analysis of benchmark datasets for Agilent two-color microarrays
Source: BMC Bioinformatics. 2007 Oct 3;8:371. doi: 10.1186/1471-2105-8-371 (PMC2174956; doi:10.1186/1471-2105-8-371)

### Supplement 4. Plots of Observed log-ratio (vertical axis) against Expected log-ratio (horizontal axis) for three versions (BA, noBA, FE) of every array.

The black line is the identity line for reference. Blue points are ERCs with true log-ratio = ±log_2_(10) ≈ ±3.32; green points are ERCs with true log-ratio = ±log_2_(3) ≈ ±1.59; red points are ERCs with true log-ratio=log_2_(1)=0.


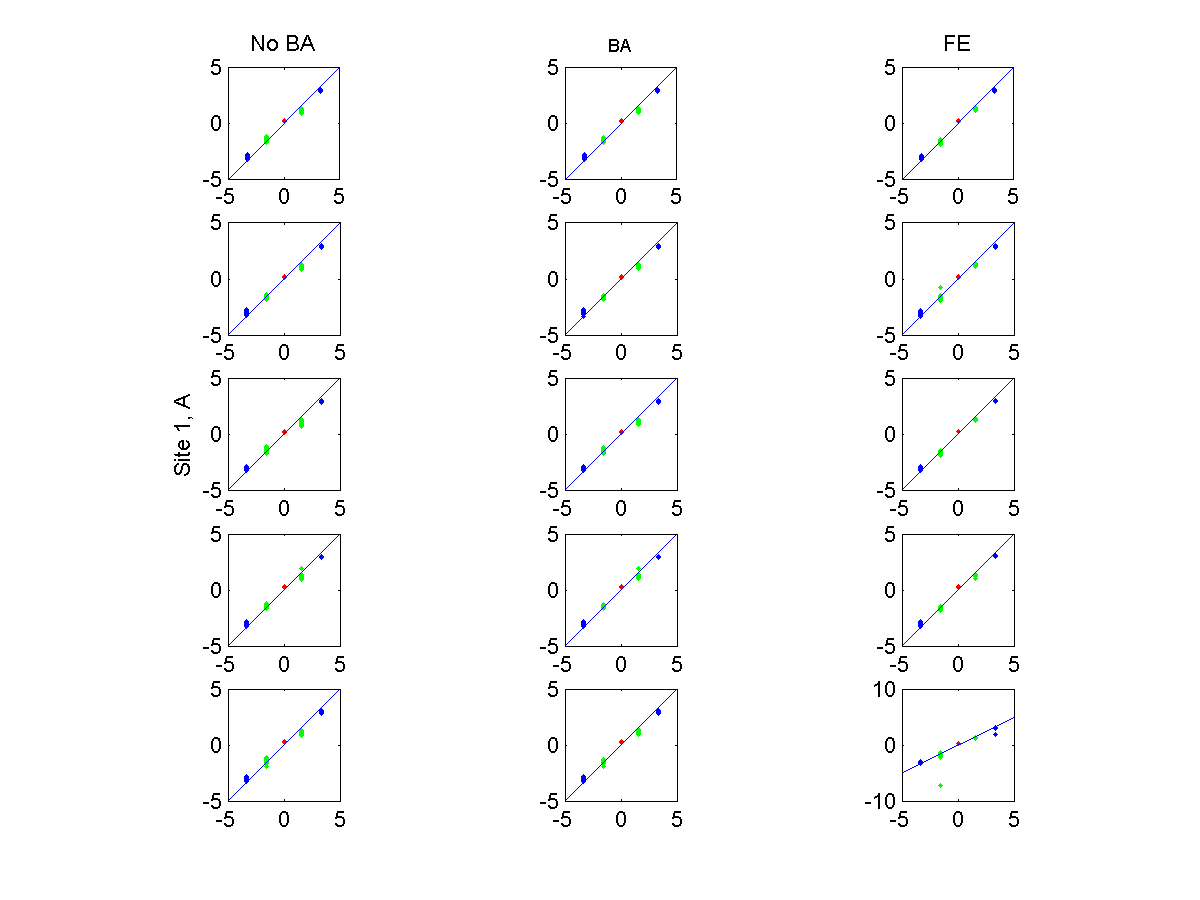

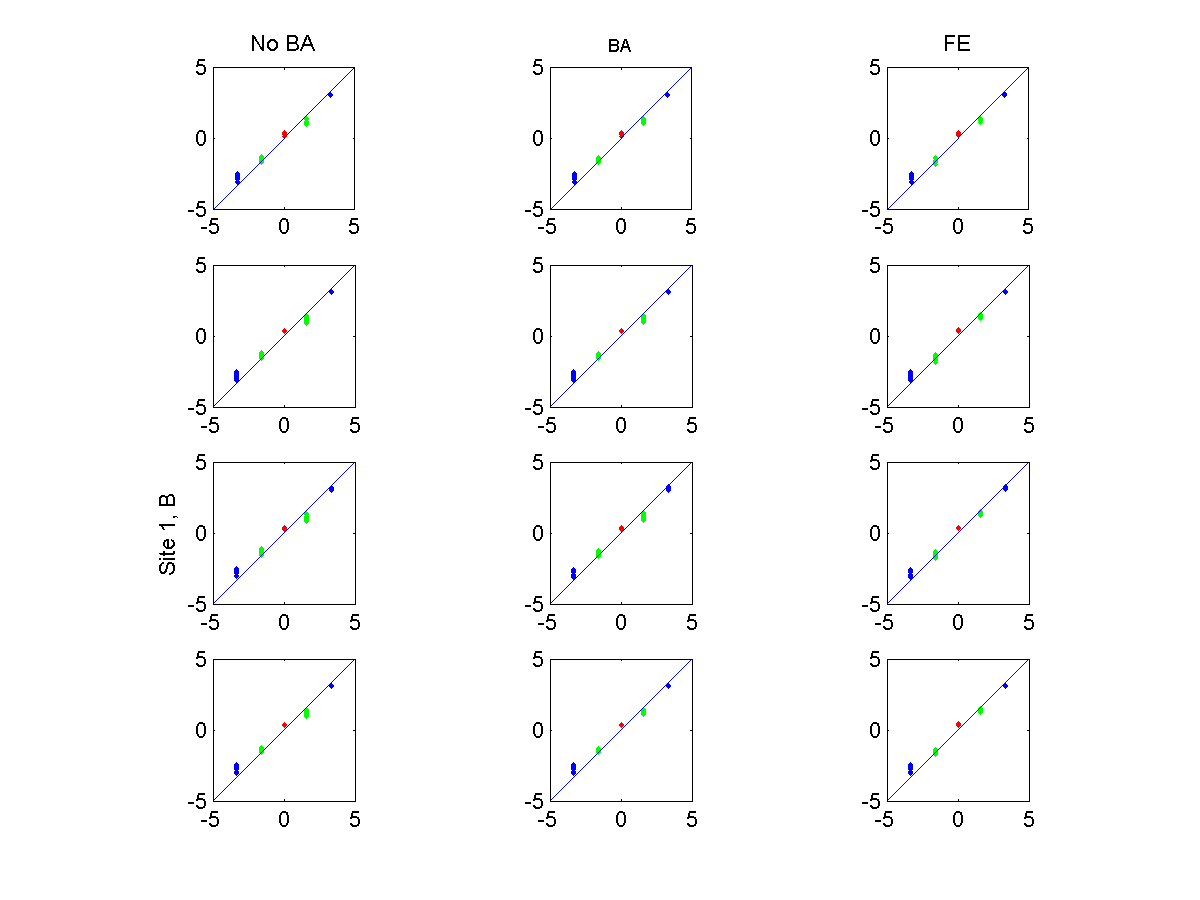

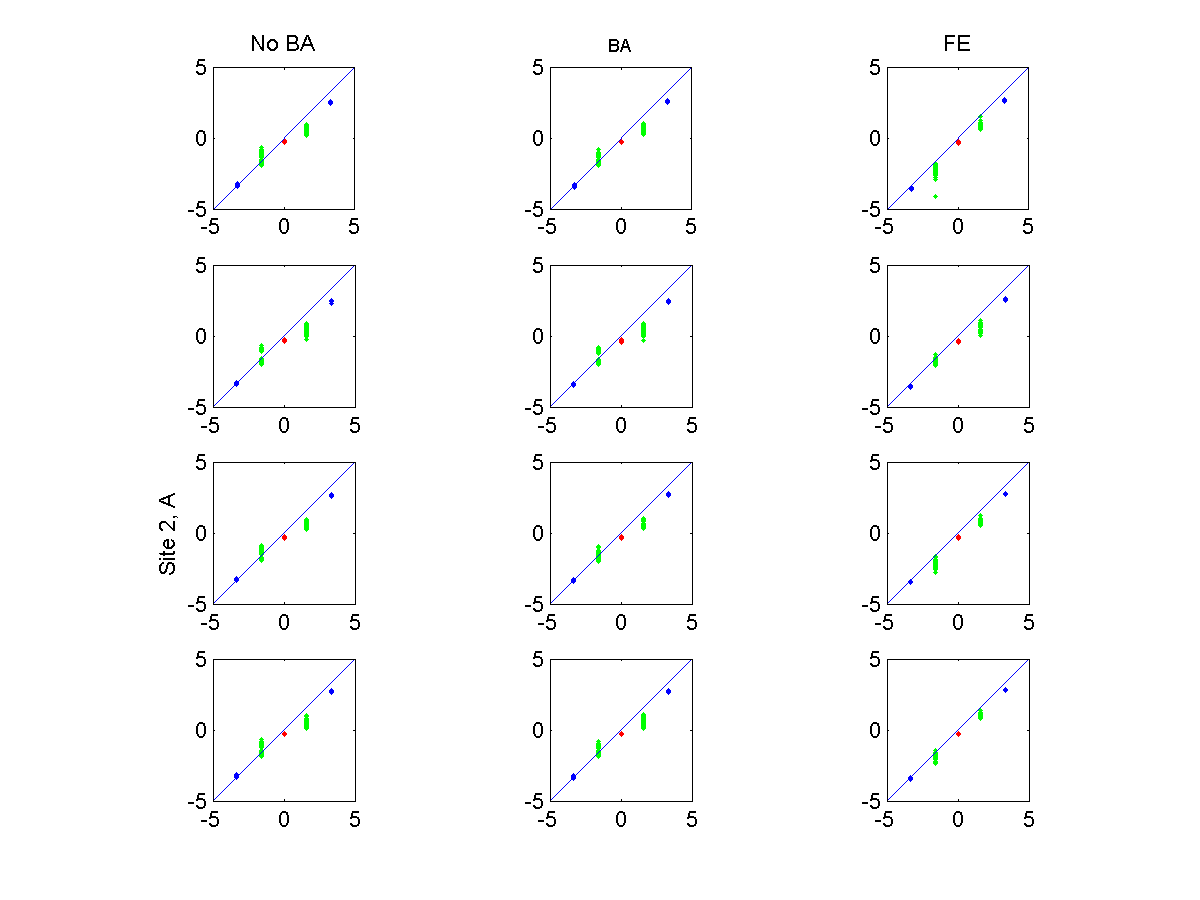

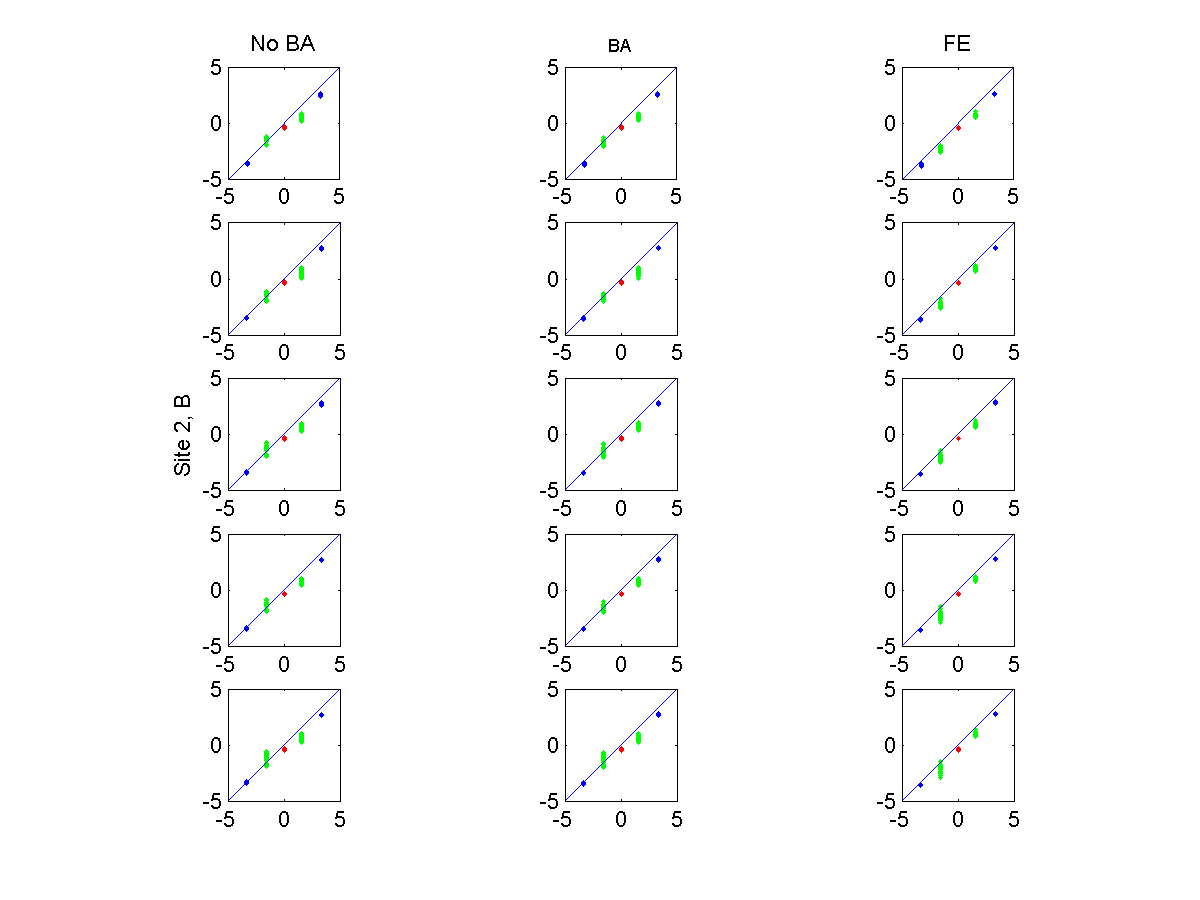

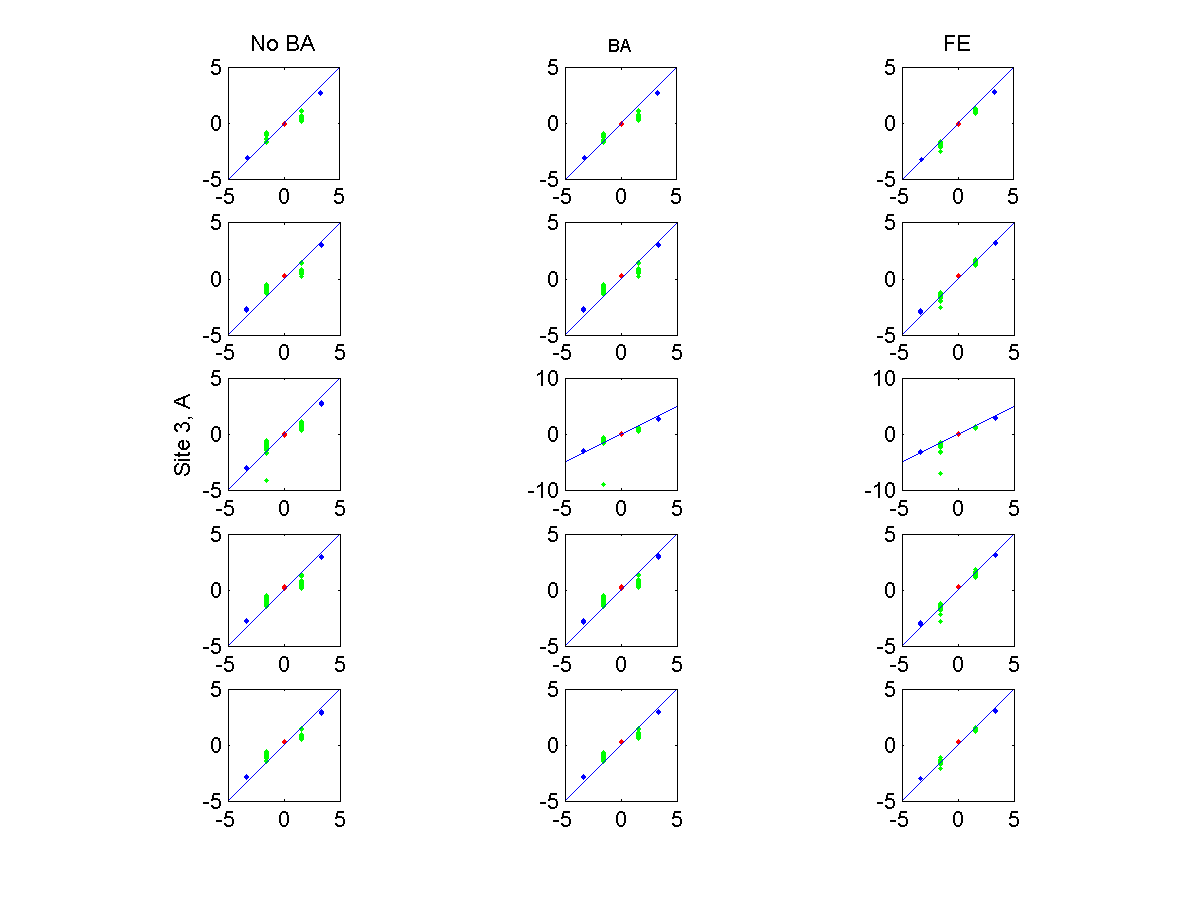

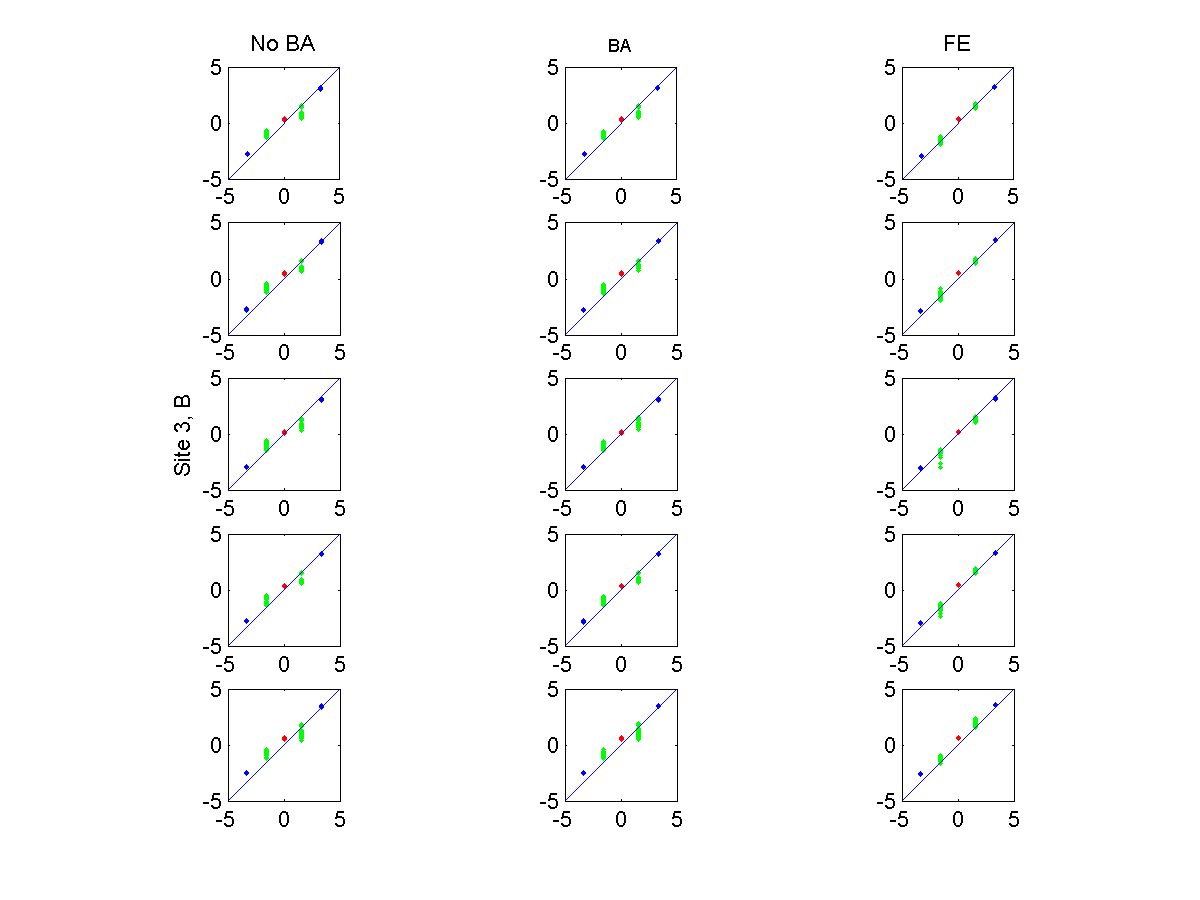

Supplement: Additional file 4 — Observed log-ratios compared to expected log-ratios. The plots show observed log-ratios compared to expected log-ratios for three versions of every array. [file 1471-2105-8-371-S4.doc]
